# Dysferlin promotes inflammatory macrophage responses and modulates Ca^2+^-dependent STAT1 signaling

**DOI:** 10.26508/lsa.202603669

**Published:** 2026-10-07

**Authors:** Kana Tominaga, Naoomi Tominaga

**Affiliations:** 1 Division of Neuropsychiatry, Department of Neuroscience, Yamaguchi University Graduate School of Medicine , Yamaguchi, Japan; 2 Division of Clinical Laboratory Sciences, Department of Nursing and Laboratory Science, Yamaguchi University Graduate School of Medicine , Yamaguchi, Japan; 3 Division of Advanced Cell Therapy, Yamaguchi University Research Institute for Cell Design Medical Science (RICeD) , Yamaguchi, Japan

## Abstract

Macrophage differentiation and activation shape tissue homeostasis and regeneration. Dysferlin (DYSF) is best known for its role in membrane repair in skeletal muscle, but its function in immune cells remains poorly understood. Here, we identify DYSF as a regulator of inflammatory macrophage–associated activation. DYSF expression increased during monocyte-to-macrophage differentiation. Gain- and loss-of-function analyses revealed that DYSF promoted inflammatory macrophage–associated responses, including cytokine production and migration. DYSF also enhanced Ca^2+^ influx and STAT1 Ser727–associated signaling, whereas *Dysf* knockdown or Ca^2+^ entry inhibition suppressed these responses. DYSF expression increased with aging in human CD14-positive cells and mouse monocyte/macrophage-lineage cells, and DYSF-expressing F4/80-positive macrophage-lineage cells accumulated in aged skeletal muscle. In a macrophage–myoblast co-culture system, DYSF-enriched inflammatory macrophages inhibited myogenic differentiation and myotube fusion, with this effect being partially mediated by soluble inflammatory factors, demonstrating an influence of macrophage DYSF on neighboring myogenic cells in vitro. Together, these findings support a role for DYSF in Ca^2+^-dependent STAT1-associated inflammatory responses in macrophages and in the suppression of myogenesis in cellular models.

## Abstract (precis)

This study links macrophage dysferlin to Ca²⁺-dependent STAT1-associated inflammatory responses and impaired myogenic differentiation in cellular models.

## Introduction

Dysferlin (DYSF) is a Ca^2+^-binding transmembrane protein characterized by multiple C2 domains that enable rapid responses to change in intracellular Ca^2+^ levels (Bansal & Campbell, 2004; Cai et al, 2009). DYSF has been extensively studied in skeletal muscle, where it mediates Ca^2+^-dependent membrane repair after mechanical injury. In addition to its role in sarcolemmal repair, DYSF localizes to T-tubules and has been implicated in the regulation of intracellular Ca^2+^ dynamics, suggesting broader functions in Ca^2+^-dependent cellular processes (Quinn et al, 2024). The absence of DYSF results in persistent Ca^2+^ influx and membrane injury, exacerbating fiber necrosis, activating proteases such as calpains, and leading to mitochondrial dysfunction and cell death (Bashir et al, 1998; Liu et al, 1998). Beyond skeletal muscle, DYSF is abundantly expressed in monocytes and macrophages (De Luna et al, 2007). Previous studies have shown that DYSF expression is up-regulated during monocyte-to-macrophage differentiation in vitro (De Morreé et al, 2013) and contributes to Ca^2+^-dependent processes such as vesicle trafficking, phagocytosis, and cell adhesion (Kesari et al, 2008; Zhang et al, 2022). *DYSF*-deficient monocytes exhibit altered inflammatory responses (Kesari et al, 2008; Nagaraju et al, 2008; Zhang et al, 2022) and increased motility (De Morreé et al, 2013), indicating that DYSF may influence macrophage functional programming. However, how DYSF regulates macrophage differentiation and activation states remains poorly understood.

Macrophages play central roles in tissue homeostasis, disease progression, and aging, and are notable for their capacity to mount excessive inflammatory responses in diverse tissues. Circulating monocytes infiltrate tissues and differentiate into macrophages that adopt distinct inflammatory or reparative programs, often described using the simplified M1- and M2-type framework (Tidball et al, 2014). Although this classification is useful for describing macrophage activation in vitro, macrophage phenotypes in vivo are highly plastic and do not always fit into a strict M1/M2 dichotomy. Therefore, inflammatory and reparative macrophage-associated states should be interpreted as dynamic activation programs rather than fixed cell identities. M1-type proinflammatory macrophages are characterized by the production of cytokines such as tumor necrosis factor-α (TNF-α) and interleukin-6 (IL-6), as well as activation of inducible nitric oxide synthase (iNOS) and signal transducer and activator of transcription 1 (STAT1) downstream of interferon gamma (IFN-γ) or lipopolysaccharide stimulation. In contrast, M2-type macrophages exhibiting reparative features are characterized by the expression of genes such as *IL-10* and *Arginase-1* (*Arg1*) and play an important role in skeletal muscle regeneration after injury (Ruffell et al, 2009). Among these signaling pathways, STAT1 is a central regulator of IFN-γ–associated inflammatory macrophage activation, and its transcriptional activity is controlled not only by canonical Tyr701 phosphorylation but also by additional regulatory events, including Ser727 phosphorylation and nuclear localization.

During aging, macrophage differentiation programs in skeletal muscle become dysregulated (Cui et al, 2019). Human studies report an increased abundance of macrophages with reparative characteristics, frequently localized within intermuscular adipose tissue, potentially driven by fibroadipogenic progenitors (FAPs) through chemokines such as C-C motif chemokine ligand 2 (CCL2) (Zhang et al, 2024). By contrast, mouse studies demonstrate an age-associated accumulation of inflammatory macrophages accompanied by chronic inflammation, oxidative stress, and activation of extracellular matrix pathways (Kawanishi & Machida, 2021). Despite these species-specific differences, aged skeletal muscle is widely recognized to exhibit a sustained proinflammatory milieu, potentially involving noncanonical macrophage activation states and systemic inflammatory cues from adipose tissue (Vogelpoel et al, 2014; De Carvalho et al, 2019; Cui et al, 2023). Functionally, prolonged inflammatory macrophage activity may impair muscle regeneration and promote atrophy, whereas macrophages with reparative features may contribute to fibrosis and adipose infiltration. Notably, both inflammatory and reparative macrophage populations are present in the skeletal muscle of *Dysf*-deficient Bla/J mice, suggesting an unresolved inflammatory environment during tissue repair (Cohen et al, 2015). However, whether DYSF expression within macrophages directly contributes to the establishment or maintenance of these macrophage programs remains unknown.

Here, we investigated the role of DYSF in macrophage differentiation and inflammatory macrophage-associated activation. Using human and murine macrophage models, we show that increased DYSF abundance promotes Ca^2+^ influx and enhances STAT1 Ser727–associated signaling, thereby promoting inflammatory macrophage–associated gene expression and cytokine production. Functionally, DYSF-enriched macrophages suppress myogenic differentiation and myotube fusion in macrophage–myoblast co-culture systems, at least in part through soluble inflammatory mediators such as TNF-α and IL-6. Notably, elevated DYSF expression is observed in monocyte/macrophage-lineage cells during aging, and DYSF-expressing F4/80-positive macrophage-lineage cells accumulate in aged skeletal muscle, suggesting that this regulatory axis may be engaged under age-associated conditions.

## Results

### DYSF is up-regulated during monocyte-to-macrophage differentiation

**Figure 1.**
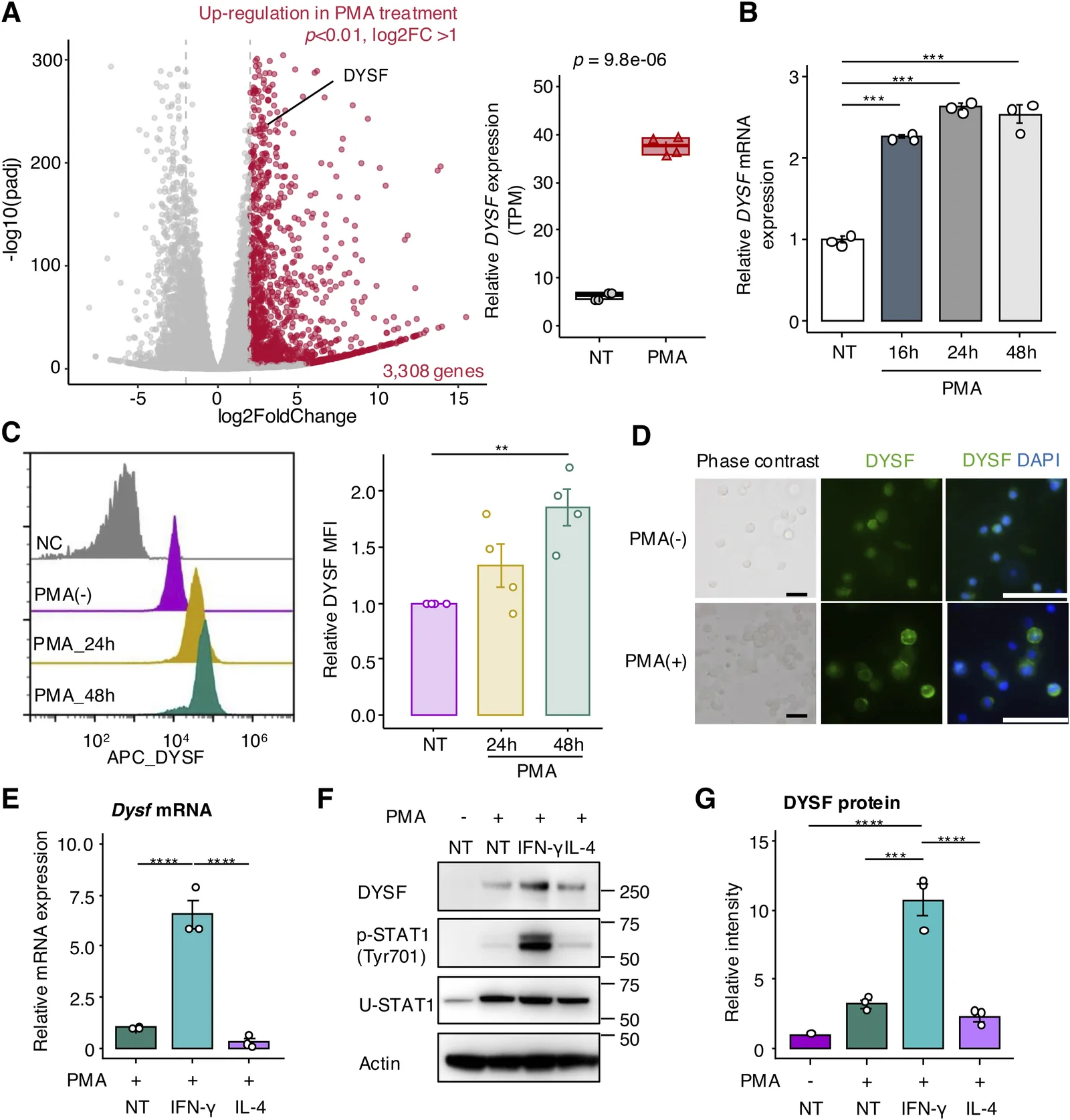
DYSF is up-regulated during monocyte-to-macrophage differentiation. **(A)** Volcano plot of RNA-seq data from GSE107566 showing differential gene expression in U937 cells treated with phorbol 12-myristate 13-acetate (PMA, 10 nM) compared with non-treated (NT) controls (left). Red dots indicate that 3,308 genes were significantly up-regulated in PMA-treated U937 cells. DYSF transcript levels are shown as log_2_-transformed transcripts per million (TPM) values (right). **(B)** Time-dependent induction of *DYSF* mRNA expression in U937 cells after PMA treatment, as determined by quantitative RT-PCR (qRT-PCR). Each dot represents one independent culture (n = 3). **(C)** Flow cytometry analysis of DYSF expression in U937 cells treated with PMA for 24 or 48 h using an anti-DYSF antibody (left). NC, negative control. Quantification of DYSF mean fluorescence intensity (MFI) is shown (right). Each dot represents one independent culture (n = 4). **(D)** Representative phase-contrast and immunofluorescence images of U937 cells treated with or without PMA and stained with an anti-DYSF antibody. Nuclei were counterstained with DAPI. Scale bars, 25 μm. **(E)** qRT-PCR analysis of endogenous *DYSF* mRNA expression in PMA-differentiated U937 macrophage–like cells treated with IFN-γ or IL-4 for 24 h. IFN-γ and IL-4 were used at 100 ng/ml. Each dot represents one independent culture (n = 3). **(F)** Immunoblot analysis of endogenous DYSF protein levels and STAT1 Tyr701 phosphorylation in untreated PMA-differentiated U937 macrophage–like cells and PMA-differentiated U937 macrophage–like cells treated with IFN-γ or IL-4 for 24 h. Phosphorylated STAT1 Tyr701 was examined as a marker of IFN-γ responsiveness. β-actin was used as a loading control. **(G)** Quantification of DYSF protein levels normalized to β-actin in (F). Each dot represents one independent culture (n = 3). Statistical analysis of DYSF transcript levels in (A, right) was performed using an unpaired two-tailed *t* test. Data in (B), (C, right), (E), and (G) were analyzed by one-way/two-way ANOVA followed by Tukeyʼs multiple comparisons test. Data are presented as mean ± SEM. Only biologically relevant comparisons are shown. **P < 0.01, ***P < 0.001, ****P < 0.0001.

To determine whether DYSF is expressed during monocyte-to-macrophage differentiation, we conducted differential gene expression analysis to identify transcriptional changes associated with monocyte differentiation. Phorbol 12-myristate 13-acetate (PMA)-induced U937 cells, a human monocyte cell line, is widely used as an in vitro model for studying human macrophage-like differentiation and function. Volcano analysis of public transcriptional data (GSE107566) (Haney et al, 2018) revealed that *DYSF* expression was up-regulated by PMA-induced differentiation (Fig 1A). We observed a time-dependent increase in *DYSF* mRNA expression in PMA-treated U937 cells compared with that in untreated cells (Fig 1B). These results were further validated by flow cytometry analysis and immunostaining in U937 cells treated with PMA, confirming the accumulation of DYSF protein after PMA treatment (Fig 1C and D). These findings indicate that DYSF is increased during monocyte-to-macrophage differentiation.

To further address whether DYSF expression is associated with macrophage activation stimuli, we examined endogenous DYSF expression in PMA-differentiated U937 macrophage–like cells treated with IFN-γ or IL-4. IFN-γ stimulation increased *DYSF* mRNA expression, whereas IL-4 did not markedly increase *DYSF* mRNA expression (Fig 1E). Immunoblot analysis further showed increased DYSF protein levels after IFN-γ stimulation, and STAT1 Tyr701 phosphorylation was confirmed as a marker of IFN-γ responsiveness (Fig 1F and G). These findings indicate that DYSF is increased during monocyte-to-macrophage differentiation and is further enhanced by inflammatory stimulation in human macrophage-like cells.

### DYSF promotes an inflammatory macrophage-like phenotype

**Figure S1.**
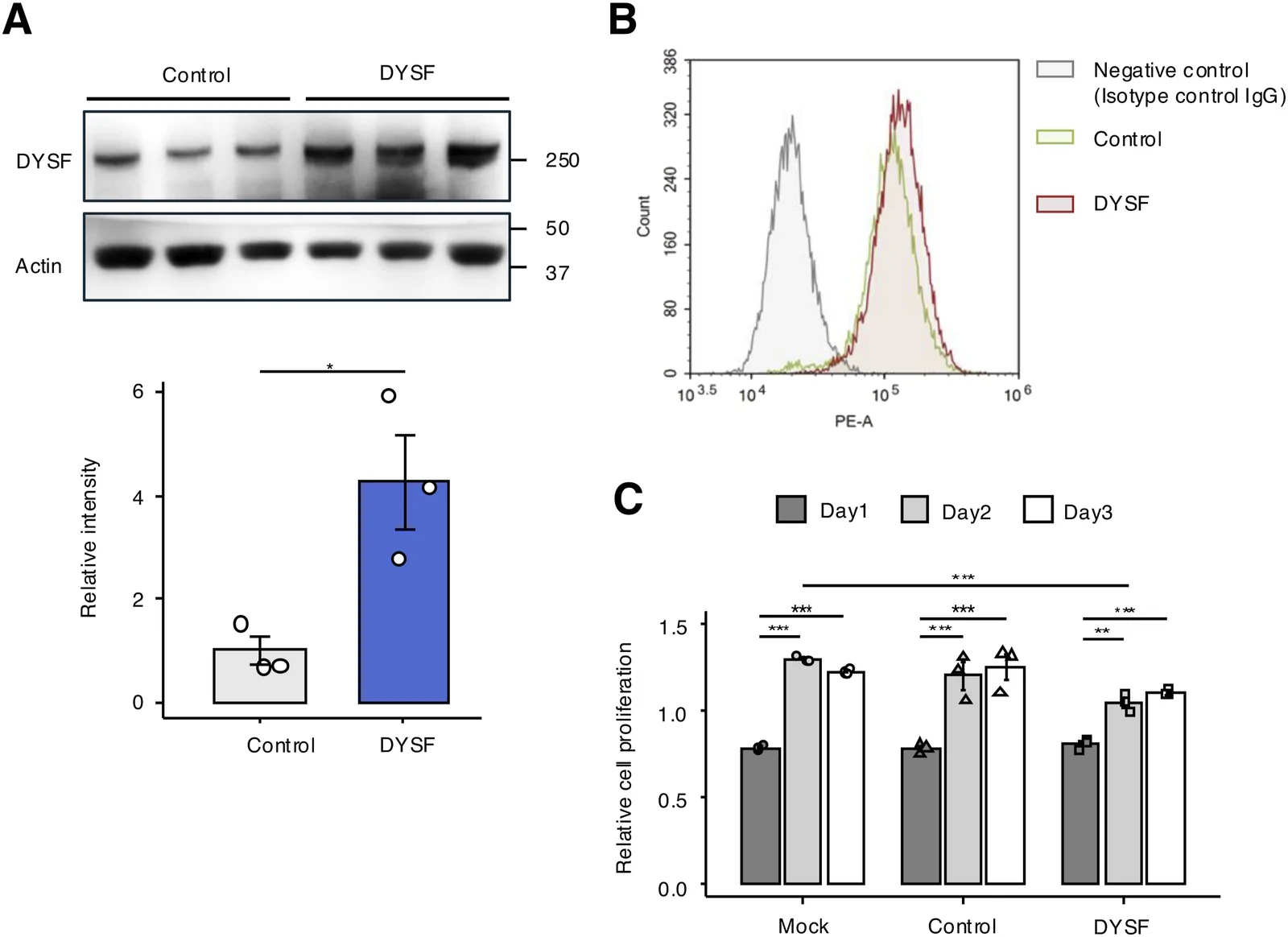
Validation of DYSF overexpression and proliferation analysis in J774A.1 macrophages. **(A)** Immunoblot analysis of DYSF protein expression in J774A.1 macrophages transiently transfected with a DYSF-3HA expression vector (DYSF) or pcDNA3.1 empty vector (Control). β-actin (Actin) was used as a loading control. DYSF protein abundance was quantified from the DYSF-immunoreactive band(s) in the expected high-molecular-weight range and normalized to β-actin. Each dot represents one independent culture (n = 3). **(B)** Flow cytometry analysis of DYSF expression in J774A.1 cells transfected with the DYSF expression vector or empty control vector. Cells stained with control IgG served as the negative control. **(C)** Cell proliferation assay of J774A.1 cells transfected with the DYSF expression vector compared with non-transfected cells (Mock) or empty control vector–transfected cells (Control). Each dot represents one independent culture (n = 3). Data are presented as mean ± SEM. Statistical analysis was performed using an unpaired two-tailed *t* test for two-group comparisons or one-way ANOVA followed by Tukeyʼs multiple comparisons test for multiple-group comparisons, as indicated. Only biologically relevant comparisons are shown. **P < 0.01, ***P < 0.001.

**Figure 2.**
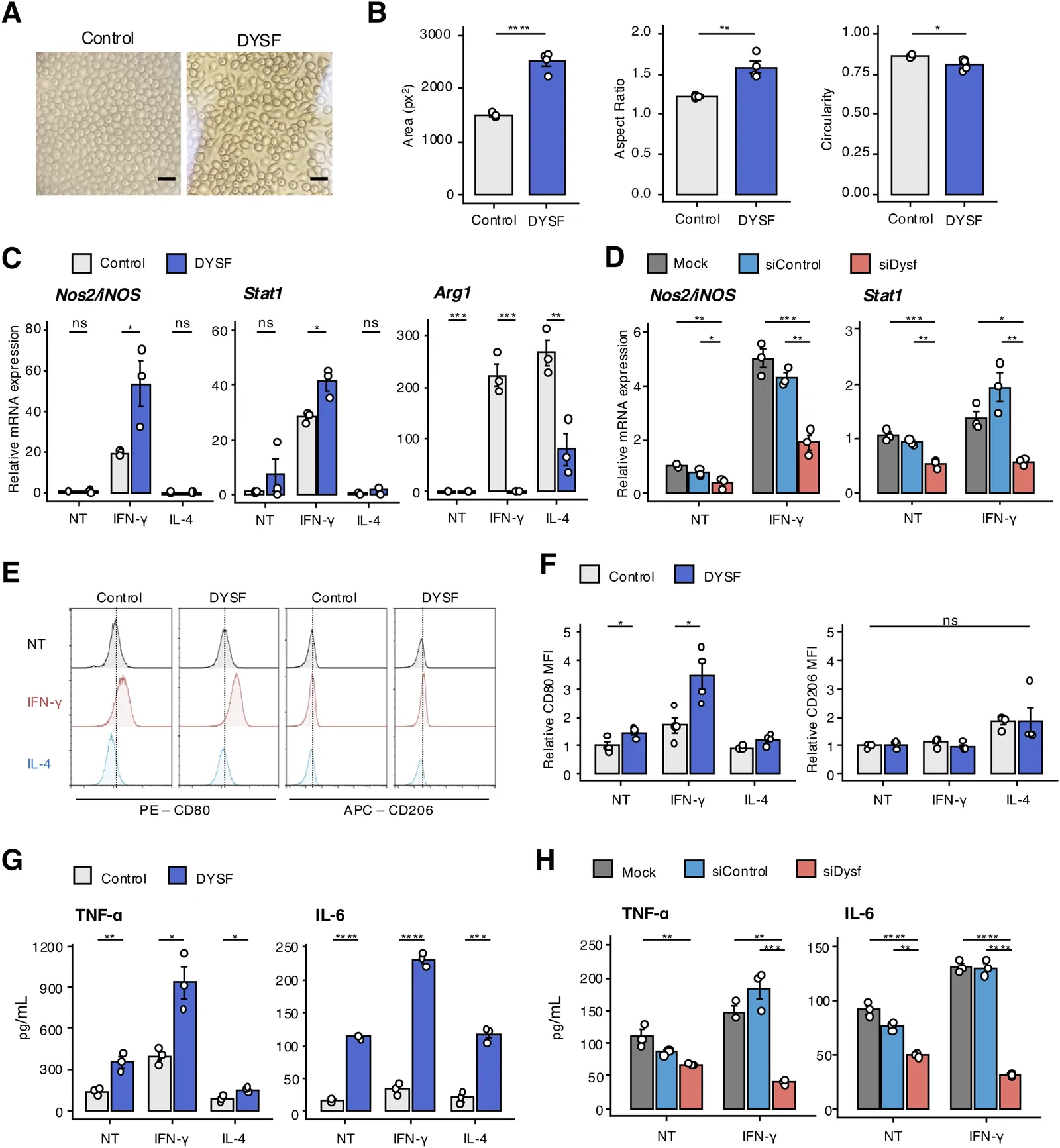
DYSF promotes an inflammatory macrophage–like phenotype in J774A.1 macrophages. **(A)** Representative phase-contrast images of J774A.1 cells transfected with DYSF expression vector (DYSF) or empty control vector (Control). Scale bar, 50 μm. **(B)** Quantification of cell morphology, including cell area, aspect ratio, and circularity. Thirty cells were analyzed per independent culture, and individual cell measurements were averaged to generate one value per culture. Each dot represents one independent culture (n = 3 biological replicates). Pre-averaged individual cell measurements are provided in the corresponding Source Data file. **(C)** qRT-PCR analysis of inflammatory macrophage-associated genes, including *NOS/iNOS* and *Stat1*, and alternative macrophage-associated genes including *Arg1* in J774A.1 cells transfected with DYSF expression vector or empty control vector under untreated or IFN-γ– or IL-4–stimulated conditions. IFN-γ and IL-4 were used at 100 ng/ml. Each dot represents one independent culture (n = 3). **(D)** qRT-PCR analysis of inflammatory genes in J774A.1 cells transfected with control siRNA or *Dysf* siRNA under IFN-γ–stimulated conditions. Mock-transfected cells were treated with transfection reagent alone without siRNA. IFN-γ was used at 100 ng/ml. Each dot represents one independent culture (n = 3). **(E)** Flow cytometry analysis of CD80 and CD206 expression in J774A.1 cells transfected with DYSF expression vector or empty control vector and treated with IFN-γ or IL-4 for 24 h. IFN-γ and IL-4 were used at 100 ng/ml. **(F)** Quantification of CD80 and CD206 mean fluorescence intensity (MFI) in (E). Each dot represents one independent culture (n = 4). **(G)** ELISA quantification of TNF-α and IL-6 secretion in conditioned media from J774A.1 cells transfected with DYSF expression vector or empty control vector under untreated or IFN-γ– or IL-4–stimulated conditions. IFN-γ and IL-4 were used at 100 ng/ml. Each dot represents one independent culture (n = 3). **(H)** ELISA quantification of TNF-α and IL-6 secretion in conditioned media from J774A.1 cells transfected with control siRNA, *Dysf* siRNA, or Mock control under untreated or IFN-γ-stimulated conditions. IFN-γ was used at 100 ng/ml. Each dot represents one independent culture (n = 3). Data are presented as mean ± SEM. Statistical analysis was performed using an unpaired two-tailed *t* test (B, C, F, G) or one-way/two-way ANOVA followed by Tukeyʼs multiple comparisons test (D, H), as indicated. For qRT-PCR and ELISA analyses, statistical tests were performed separately for each gene or cytokine. Only biologically relevant comparisons are shown. *P < 0.05, **P < 0.01, ***P < 0.001, ****P < 0.0001; ns, not significant. Source data are available for this figure.

**Figure S2.**
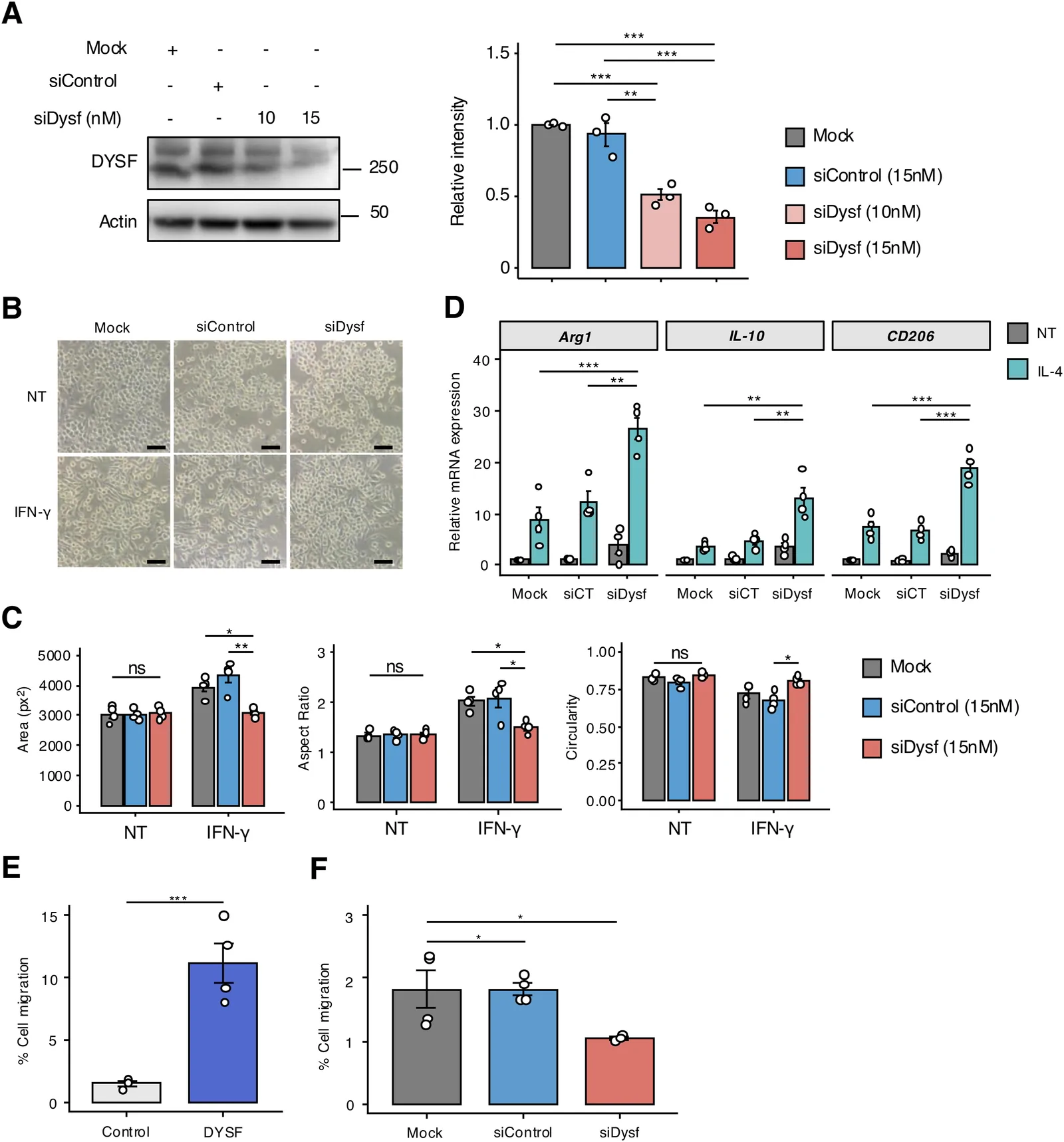
DYSF knockdown attenuates IFN-γ–induced macrophage activation and alters macrophage-associated responses. **(A)** Immunoblot analysis of Dysf protein expression in J774A.1 macrophages transiently transfected with *Dysf*-targeting siRNA (siDysf), non-targeting control siRNA (siControl), or mock control. Mock-transfected cells were treated with transfection reagent alone without siRNA. Densitometric quantification of Dysf protein levels normalized to β-actin (Actin) is shown on the right. Each dot represents one independent culture (n = 3). **(B)** Representative phase-contrast images of J774A.1 cells transfected with control siRNA or *Dysf* siRNA under IFN-γ–stimulated conditions. IFN-γ was used at 100 ng/ml. Scale bar, 50 μm. **(C)** Quantification of cell morphology parameters, including cell area, circularity, and aspect ratio in siDysf- and siControl-transfected J774A.1 cells under untreated or IFN-γ–stimulated conditions. IFN-γ was used at 100 ng/ml. Thirty cells were analyzed per independent culture, and individual cell measurements were averaged to generate one value per culture. Each dot represents one independent culture (n = 3 biological replicates). Pre-averaged individual cell measurements are provided in the corresponding Source Data file. **(D)** qRT-PCR analysis of alternative macrophage-associated genes, *Arg1*, *IL-10*, and *CD206*, in J774A.1 cells transfected with control siRNA, *Dysf* siRNA, or mock control under IL-4–stimulated conditions. Mock-transfected cells were treated with transfection reagent alone without siRNA. IL-4 was used at 100 ng/ml. Each dot represents one independent culture (n = 4). **(E)** Transwell migration assay of J774A.1 cells transfected with DYSF expression vector or empty control vector. Each dot represents one independent culture (n = 4). **(F)** Transwell migration assay of J774A.1 cells transfected with control siRNA or *Dysf* siRNA. Each dot represents one independent culture (n = 4). Data are presented as mean ± SEM. Statistical analysis was performed using an unpaired two-tailed *t* test for two-group comparisons (E) or one-way/two-way ANOVA followed by Tukeyʼs multiple comparisons test for multiple-group comparisons (A, D, C), as indicated. For qRT-PCR analyses, statistical tests were performed separately for each gene. Only biologically relevant comparisons are shown. *P < 0.05, **P < 0.01, ***P < 0.001; ns, not significant. Source data are available for this figure.

We next examined whether high DYSF expression in macrophages influenced their activation state. We transfected J774A.1 cells, a murine monocyte/macrophage cell line, with a DYSF expression plasmid (DYSF; DYSF-3HA) or a control empty vector (Control; pcDNA3.1), and confirmed DYSF overexpression by immunoblotting and flow cytometry (Fig S1A and B). We then measured cell proliferation, cell area, aspect ratio, and circularity to characterize DYSF-overexpressing cells. Compared with control cells, DYSF-overexpressing cells showed significantly reduced cell proliferation but exhibited morphological features associated with macrophage activation and differentiation (Fig S1C). Cell area and aspect ratio increased with differentiation, whereas circularity decreases (Moss et al, 2024). Consistent with these features, DYSF-overexpressing cells were larger, more elongated, and less circular than control cells (Fig 2A and B). We next performed knockdown experiments in J774A.1 cells using small interfering RNA (siRNA) targeting *Dysf* (Fig S2A). Under IFN-γ-stimulated conditions, *Dysf* knockdown reduced cell area and aspect ratio, whereas circularity was conversely increased (Fig S2B and C). These findings indicate that DYSF is associated with morphological remodeling of macrophage-like cells, although cell morphology alone cannot define macrophage differentiation or activation state.

To elucidate the role of DYSF in macrophage activation, we evaluated macrophage-associated gene expression in J774A.1 cells exposed to specific cytokine stimuli. J774A.1 macrophages were treated with IFN-γ or IL-4, and the expression of inflammatory macrophage-associated genes, including *Nos2/iNOS* and *Stat1*, and an alternative macrophage-associated gene, *Arg1*, was assessed. Compared with control cells, DYSF-overexpressing cells showed significantly elevated *Nos2/iNOS* and *Stat1* mRNA levels, indicating that DYSF promotes an inflammatory macrophage-like phenotype (Fig 2C). Conversely, *Arg1* mRNA expression was induced by IL-4 but was not enhanced by DYSF overexpression, suggesting that DYSF preferentially promotes inflammatory macrophage-associated gene expression rather than broadly enhancing alternative macrophage-associated gene expression (Fig 2C).

To further investigate the role of DYSF in inflammatory macrophage-associated responses, we examined the macrophage phenotypes of *DYSF* siRNA-transfected J774A.1 cells treated with IFN-γ. Compared with mock cells and control cells, *Dysf*-knockdown cells treated with IFN-γ exhibited markedly reduced *Nos2/iNOS* and *Stat1* mRNA expression (Fig 2D). In contrast, *Dysf* knockdown enhanced IL-4–induced alternative macrophage-associated gene expression, suggesting that DYSF may limit the acquisition of an IL-4–induced alternative macrophage-associated phenotype (Fig S2D). Together, these gain- and loss-of-function experiments indicate that DYSF modulates macrophage activation states by promoting inflammatory macrophage-associated responses.

To further elucidate the phenotype of macrophage activation phenotypes, we conducted flow cytometry analysis on the basis of cell surface marker expression. J774A.1 macrophages treated with IFN-γ exhibited elevated CD80 expression, whereas IL-4–treated cells showed increased CD206 expression. Compared with IFN-γ–stimulated control cells, DYSF-overexpressing cells stimulated with IFN-γ showed increased CD80 intensity, supporting a shift toward inflammatory macrophage–like phenotype (Fig 2E and F). Conversely, IL-4 treatment increased CD206 expression, but DYSF overexpression did not significantly enhance CD206 intensity compared with control cells. These findings indicate that DYSF preferentially enhances inflammatory macrophage–associated features.

Inflammatory macrophages activated by IFN-γ stimulation secrete various proinflammatory cytokines. To investigate whether DYSF influences proinflammatory macrophage cytokine secretion, we analyzed the secretion of TNF-α and IL-6 in conditioned media of cytokine-stimulated macrophages. Compared with control cells, DYSF-overexpressing cells exhibited higher protein concentrations of TNF-α and IL-6 under untreated and IFN-γ–stimulated conditions (Fig 2G). We subsequently assessed whether the expression of proinflammatory cytokines was altered by *Dysf* knockdown. The ELISA results indicated that *Dysf* knockdown reduced secretion of TNF-α and IL-6 in IFN-γ–stimulated J774A.1 cells compared with control siRNA-transfected cells (Fig 2H). These results suggest that DYSF regulates the production of proinflammatory factors in macrophages.

Inflammatory macrophages are recognized for their proinflammatory function and migrate to sites of infection or injury to initiate immune response (Tidball et al, 2014). To assess whether DYSF influences macrophage migration, we conducted Transwell assays. We cultured DYSF-overexpressing or control J774A.1 cells on Matrigel-coated membranes for 48 h and subsequently quantified the cells that migrated to the bottom wells across the membrane. DYSF overexpression increased macrophage migration compared with control cells (Fig S2E). Conversely, *Dysf* knockdown reduced J774A.1 cell migration compared with control siRNA-transfected cells (Fig S2F). These findings suggest that DYSF contributes to macrophage migration.

### DYSF regulates Ca^2+^ influx in macrophages

**Figure S3.**
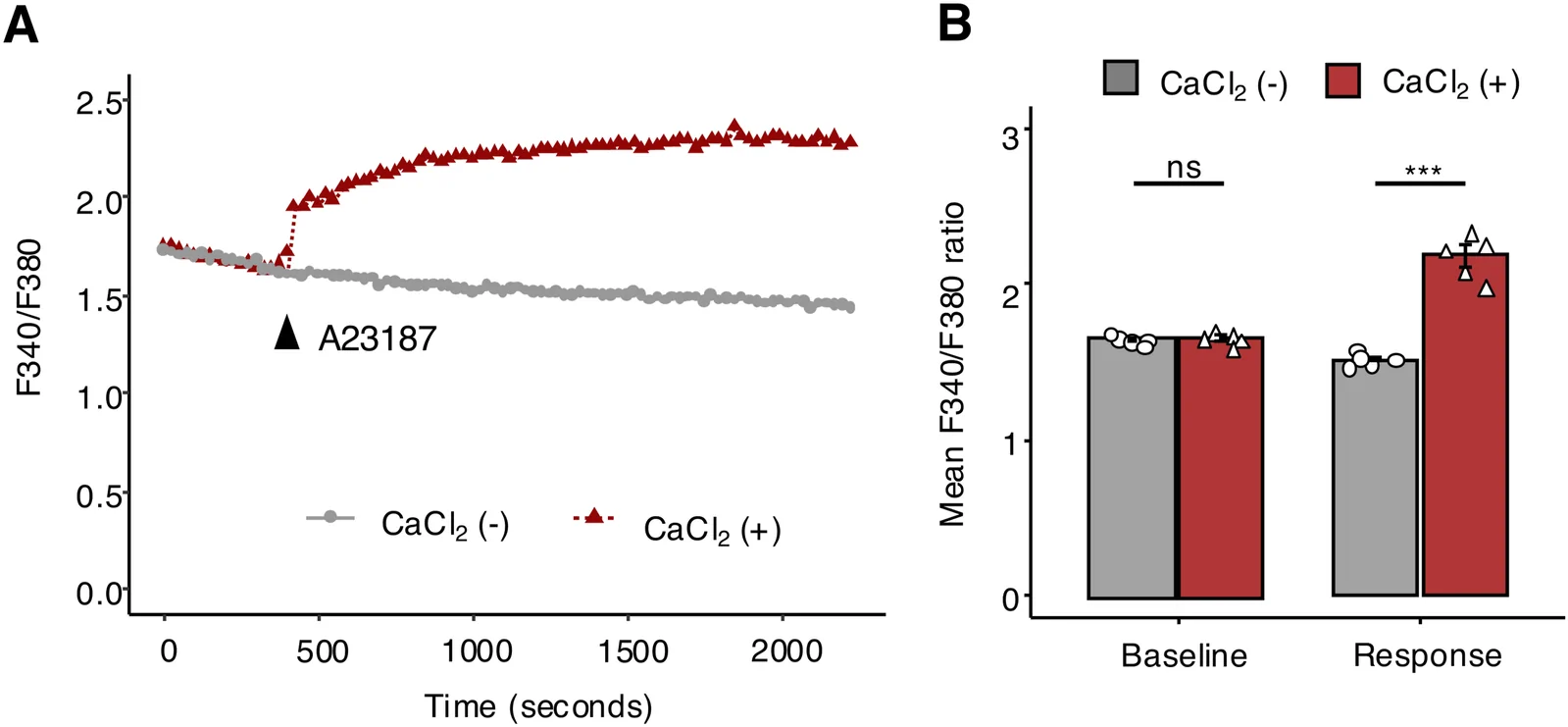
Effect of extracellular calcium on intracellular Ca^2+^ flux. **(A)** J774A.1 macrophages were loaded with 4 μg/ml Fura-2 AM for 1 h at 37°C in the presence or absence of extracellular Ca^2+^. During fluorescence measurement, the calcium ionophore A23187 (2 μM) was added to mobilize intracellular Ca^2+^ stores. Intracellular Ca^2+^ levels were monitored as changes in the Fura-2 fluorescence ratio using excitation wavelengths of 340 and 380 nm and emission measured at 510 nm. **(B)** Average of baseline and peak Ca^2+^ responses in (A) (n = 5). Data are presented as mean ± SD. Statistical analysis was performed using unpaired two-tailed *t* test. ***P < 0.001; ns, not significant.

**Figure 3.**
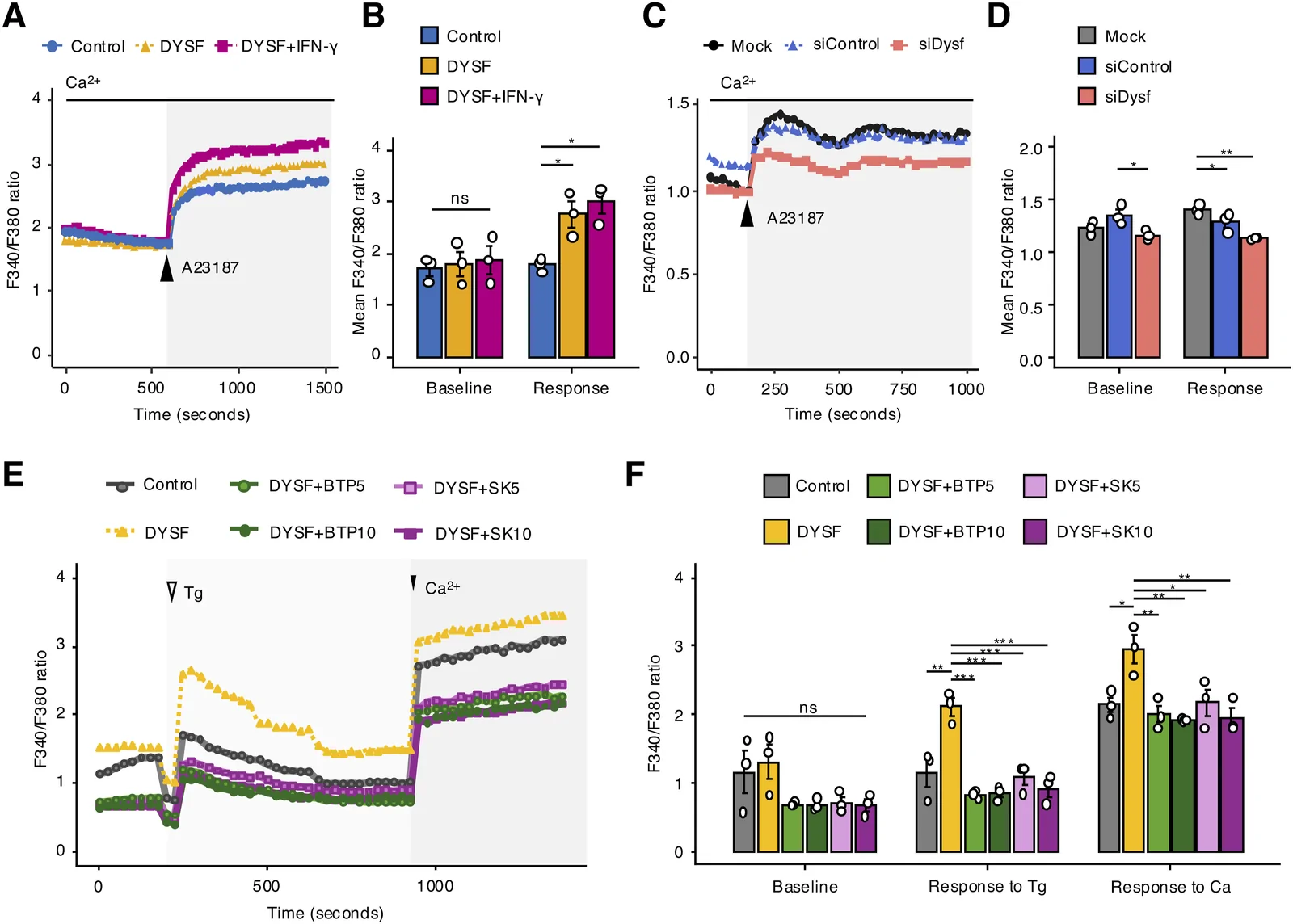
DYSF regulates Ca^2+^ influx in macrophages. **(A)** Representative intracellular Ca^2+^ responses to A23187 (2 μM) in J774A.1 cells transfected with DYSF expression vector (DYSF) or empty control vector (Control), with or without IFN-γ treatment (100 ng/ml, 24 h). Ca^2+^ levels were measured as F340/F380 ratios. Each trace shows the mean F340/F380 response from multiple wells in one representative independent experiment. **(B)** Quantification of baseline and peak Ca^2+^ responses under the conditions shown in (A). Values from multiple wells within the same independent experiment were averaged before statistical analysis. Each dot represents one independent experiment (n = 3). **(C)** Representative intracellular Ca^2+^ responses to A23187 (2 μM) in J774A.1 cells transfected with *Dysf* siRNA, control siRNA, or mock-treated cells. Ca^2+^ levels were measured as F340/F380 ratios. Each trace shows the mean F340/F380 response from multiple wells in one representative independent experiment. **(D)** Quantification of baseline and peak Ca^2+^ responses under the conditions shown in (C). Values from multiple wells within the same independent experiment were averaged before statistical analysis. Each dot represents one independent experiment (n = 3). **(E)** Representative store-operated Ca^2+^ entry (SOCE) in DYSF-transfected J774A.1 cells pretreated with SKF96365 (SKF, 5 or 10 μM) or BTP2 (BTP, 5 or 10 μM). Thapsigargin (Tg, 2 μM) was applied in Ca^2+^-free conditions followed by Ca^2+^ re-addition. BTP2 and SKF96365 were prepared in DMSO. For vehicle controls, cells were treated with the same final concentration of DMSO as that used in the inhibitor-treated groups. Each trace shows the mean F340/F380 response from multiple wells in one representative independent experiment. **(F)** Quantification of baseline Ca^2+^ levels, Tg-induced Ca^2+^ release (first peak), and SOCE (second peak) under the conditions shown in (E). Values from multiple wells within the same independent experiment were averaged before statistical analysis. Each dot represents one independent experiment (n = 3). Data are presented as mean ± SEM. Statistical analysis was performed using one-way/two-way ANOVA followed by Tukeyʼs multiple comparisons test (B, D, F) and using one-way ANOVA + Dunnettʼs multiple comparisons test (I). Only biologically relevant comparisons are shown. *P < 0.05, **P < 0.01, ***P < 0.001; ns, not significant.

To elucidate the molecular mechanism by which DYSF promotes an inflammatory macrophage-like phenotype, we next investigated upstream signaling events associated with DYSF function. Given the established role of DYSF in membrane repair and Ca^2+^ handling in myocytes, we hypothesized that DYSF may regulate intracellular Ca^2+^ dynamics during macrophage activation. To determine whether DYSF affects the regulation of Ca^2+^ concentrations within macrophages, we monitored intracellular Ca^2+^ levels in J774A.1 cells under conditions of both DYSF overexpression and knockdown. Addition of A23187, a calcium ionophore, increased the Fura-2 ratio in presence of Ca^2+^ levels, indicating an increase in intracellular Ca^2+^ levels (Fig S3A and B). Compared with control cells, DYSF-overexpressing cells exhibited a significant increase in intracellular Ca^2+^ levels after treatment with A23187 (Fig 3A and B). Furthermore, IFN-γ treatment of DYSF-overexpressing cells resulted in a further increase in intracellular Ca^2+^ levels, suggesting that DYSF enhances Ca^2+^ influx capacity under inflammatory stimulation. Conversely, when we monitored Ca^2+^ influx in J774A.1 cells with reduced Dysf expression, Ca^2+^ influx after A23187 treatment was significantly lower in *Dysf*-knockdown cells than in mock and control siRNA-transfected cells (Fig 3C and D). We also examined store-operated Ca^2+^ entry using thapsigargin, a SERCA pump inhibitor that depletes ER Ca^2+^ stores, together with the Ca^2+^ entry inhibitors BTP2, also known as YM-58483, and SKF96365. Thapsigargin treatment induced an initial increase in intracellular Ca^2+^ levels, reflecting ER Ca^2+^ release, followed by Ca^2+^ entry after extracellular Ca^2+^ re-addition in both DYSF-overexpressing and control J774A.1 cells (Fig 3E and F). In DYSF-overexpressing cells, BTP2 and SKF96365 reduced thapsigargin-induced Ca^2+^ release and subsequent Ca^2+^ entry. These results suggest that DYSF regulates intracellular Ca^2+^ handling and enhances store-operated Ca^2+^ entry in macrophages.

### DYSF enhances Ca^2+^-dependent STAT1 Ser727 phosphorylation and STAT1-responsive gene expression

Ca^2+^ influx can modulate STAT1 activity, including phosphorylation and subsequent nuclear translocation, whereas STAT1 itself can influence Ca^2+^ signaling pathways and related gene expression (Jayasree et al, 2002). IFN-γ–induced inflammatory macrophage activation is directly linked to STAT1 and is integral to the characteristics of this macrophage-associated phenotype (Chen et al, 2023). IFN-γ also increases the production of TNF-α and IL-6 by activating STAT1, which functions as a transcription factor. Therefore, to identify the molecules associated with DYSF-dependent macrophage activation, we investigated the endogenous protein levels and phosphorylation status of key molecules in the STAT pathway.

**Figure 4.**
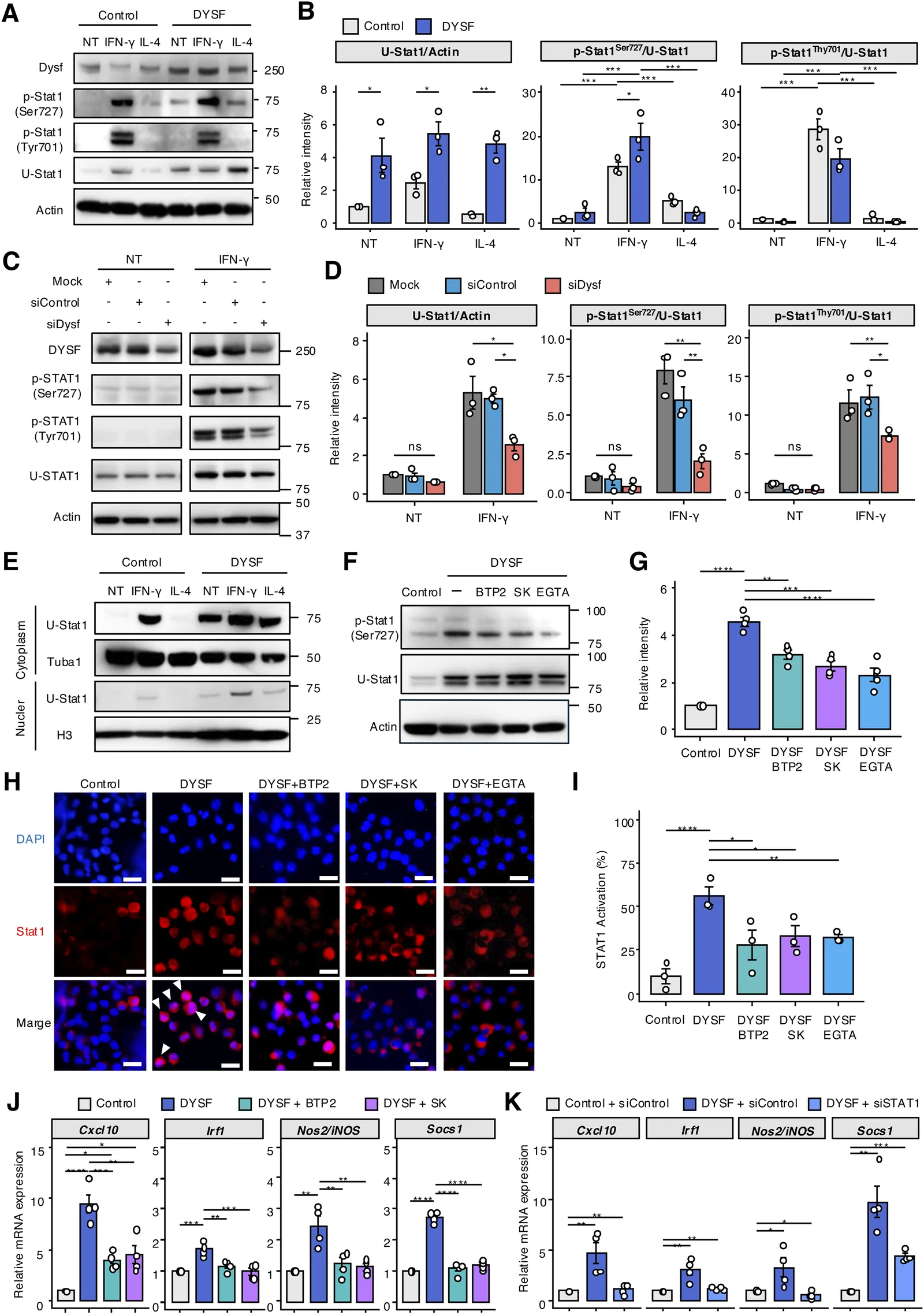
DYSF enhances Ca^2+^-dependent STAT1 Ser727 phosphorylation and STAT1-responsive gene expression in macrophages. **(A)** Immunoblot analysis of DYSF expression and STAT1 phosphorylation in J774A.1 cells transfected with DYSF expression vector (DYSF) or empty control vector (Control) and treated with or without 100 ng/ml IFN-γ or 100 ng/ml IL-4. β-actin (Actin) was used as a loading control. **(B)** Quantification of total STAT1 and STAT1 phosphorylation at Ser727 and Tyr701 in (A). Each dot represents one independent culture (n = 3). **(C)** Immunoblot analysis of DYSF expression and STAT1 phosphorylation in J774A.1 cells transfected with control siRNA or *Dysf* siRNA under IFN-γ–stimulated conditions. Mock-transfected cells were treated with transfection reagent alone without siRNA. IFN-γ was used at 100 ng/ml. β-actin (Actin) was used as a loading control. **(D)** Quantification of total STAT1 and STAT1 phosphorylation at Ser727 and Tyr701 in (C). Each dot represents one independent culture (n = 3). **(E)** Immunoblot analysis of nuclear and cytoplasmic STAT1 in J774A.1 cells transfected with DYSF or control vector. Histone H3 and α-tubulin (Tuba1) were used as nuclear and cytoplasmic loading controls, respectively. **(F)** Immunoblot analysis of STAT1 phosphorylation in DYSF-overexpressing J774A.1 cells treated with 10 μM BTP2, 10 μM SKF96365, or Ca^2+^-free/1 mM EGTA conditions. DMSO was used as the vehicle control for BTP2 and SKF96365 treatment. β-actin was used as a loading control. **(G)** Quantification of total STAT1 and STAT1 phosphorylation at Ser727 in (F). Each dot represents one independent culture (n = 4). **(H)** Representative immunofluorescence images showing STAT1 localization (red) and nuclei (DAPI, blue) in DYSF-overexpressing J774A.1 cells treated with 10 μM BTP2, 10 μM SKF96365, or Ca^2+^-free/1 mM EGTA conditions. Scale bar, 25 μm. **(I)** Quantification of nuclear STAT1 localization in (H). STAT1 nuclear fluorescence was quantified from 10 microscopic fields per independent culture. Field-level measurements were averaged to generate one value per independent culture. Each dot represents one biological replicate (n = 3 independent experiments). Pre-averaged measurements are provided in the corresponding Source Data file. **(J)** qRT-PCR analysis of STAT1-responsive genes, including *Cxcl10*, *Irf1*, *Nos2/iNOS*, and *Socs1*, in J774A.1 cells transfected with control vector or DYSF expression vector and treated with vehicle, 10 μM BTP2, or 10 μM SKF96365, as indicated. Each dot represents one independent culture (n = 4). **(K)** qRT-PCR analysis of STAT1-responsive genes in J774A.1 cells transfected with control vector plus control siRNA, DYSF expression vector plus control siRNA, or DYSF expression vector plus *Stat1* siRNA. Each dot represents one independent culture (n = 4). Data are presented as mean ± SEM. Each dot represents one independent experiment or independent culture, unless otherwise indicated. Statistical analysis was performed using one-way ANOVA followed by Tukeyʼs multiple comparisons test (B, D, I, J, K), unless otherwise indicated. For qRT-PCR analyses, statistical tests were performed separately for each gene. Only biologically relevant comparisons are shown. *P < 0.05, **P < 0.01, ***P < 0.001, ****P < 0.0001; ns, not significant. Source data are available for this figure.

Immunoblot analysis revealed that DYSF overexpression increased the levels of unphosphorylated STAT1 (U-STAT1) and the phosphorylation of STAT1 at Ser727, whereas phosphorylation of STAT1 at Tyr701 was not markedly enhanced by DYSF overexpression compared with control cells (Fig 4A and B). To further explore the involvement of DYSF in the STAT1 signaling pathway, we assessed U-STAT1 and STAT1 phosphorylation in *DYSF* siRNA-transfected J774A.1 cells. Compared with mock and control siRNA-transfected cells, *Dysf*-knockdown cells exhibited reduced U-STAT1 expression and decreased STAT1 Ser727 and Tyr701 phosphorylation under IFN-γ–stimulated conditions (Fig 4C and D). These results suggest that DYSF preferentially modulates a Ser727-associated regulatory component of STAT1 signaling rather than simply enhancing canonical Tyr701-dependent STAT1 activation.

**Figure S4.**
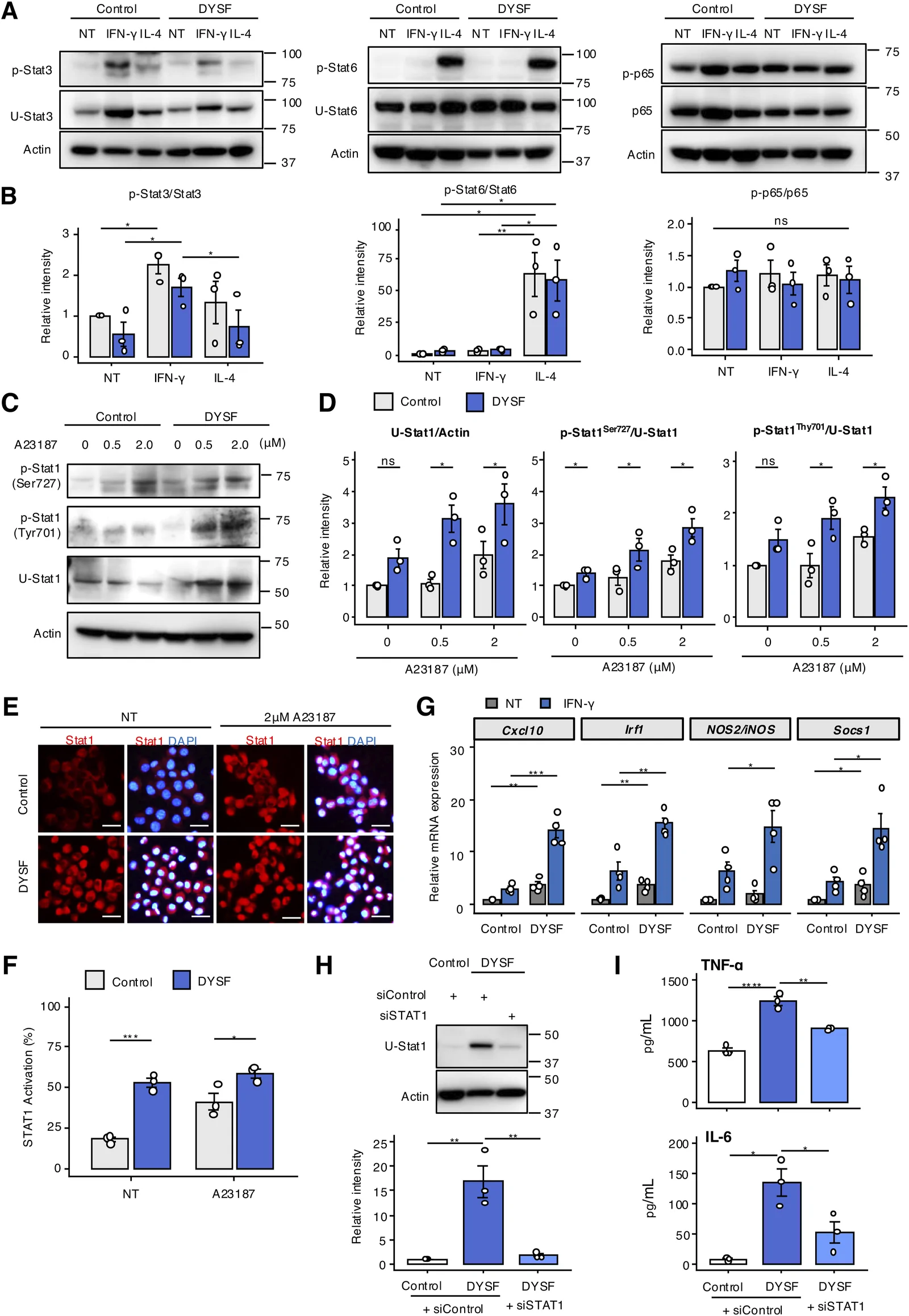
Additional analyses of STAT signaling and A23187-induced STAT1 activation in DYSF-overexpressing macrophages. **(A)** Immunoblot analysis of STAT3, STAT6, and NF-κB p65 phosphorylation in J774A.1 cells transfected with DYSF expression vector (DYSF) or empty control vector (Control) and treated with or without IFN-γ or IL-4. IFN-γ and IL-4 were used at 100 ng/ml. β-actin (Actin) was used as a loading control. **(B)** Quantification of total and phosphorylated STAT3, STAT6, and NF-κB p65 protein levels in (A). Each dot represents one independent culture (n = 3). **(C)** Immunoblot analysis of STAT1 phosphorylation in J774A.1 cells transfected with DYSF or empty vector and treated with A23187 at the indicated concentrations (0, 0.5, or 2 μM). β-actin (Actin) was used as a loading control. **(D)** Quantification of total STAT1 and STAT1 phosphorylation at Ser727 and Tyr701 in (C). Each dot represents one independent culture (n = 3). **(E)** Representative immunofluorescence images showing STAT1 localization (red) and nuclei (DAPI, blue) in J774A.1 cells transfected with DYSF or empty control vector and treated with or without A23187 (2 μM). Scale bar, 25 μm. **(F)** Quantification of nuclear STAT1 localization in (C). STAT1 nuclear fluorescence was quantified from five microscopic fields per independent culture. Field-level measurements were averaged to generate one value per independent culture. Each dot represents one biological replicate (n = 3 independent experiments). Pre-averaged measurements are provided in the corresponding Source Data file. **(G)** Immunoblot analysis of STAT1 protein expression in J774A.1 macrophages transiently transfected with *STAT1*-targeting siRNA (siSTAT1), non-targeting control siRNA (siControl). Densitometric quantification of STAT1 protein levels normalized to β-actin (Actin) is shown on the bottom. Each dot represents one independent culture (n = 3). **(H)** qRT-PCR analysis of STAT1-responsive genes, *Cxcl10, Irf1*, *Nos2/iNOS*, and *Socs1* in J774A.1 cells transfected with DYSF expression vector or control vector treated with or without 100 ng/ml IFN-γ. Each dot represents one independent culture (n = 4). **(I)** ELISA analysis of TNF-α and IL-6 levels in conditioned media from DYSF-overexpressing J774A.1 macrophages transfected with control siRNA or *Stat1* siRNA and control. Each dot represents one independent culture. Data are presented as mean ± SEM. Statistical analysis was performed using a one-way/two-way ANOVA followed by Tukeyʼs multiple comparisons test for multiple-group comparisons, as indicated. For qRT-PCR analyses, statistical tests were performed separately for each gene. *P < 0.05, **P < 0.01, ***P < 0.001, ****P < 0.0001; ns, not significant. Source data are available for this figure.

To address whether DYSF broadly affects other macrophage-associated signaling pathways, we also examined STAT3, STAT6, and NF-κB p65 phosphorylation. Under these conditions, DYSF overexpression did not markedly alter the phosphorylation levels of STAT3, STAT6, or NF-κB p65 compared with control cells, with or without IFN-γ or IL-4 stimulation (Fig S4A and B). These results further support the preferential association of DYSF with STAT1 Ser727–associated signaling in this experimental setting.

To investigate the nuclear translocation of STAT1 in DYSF-overexpressing and control cells, we assessed the amount of U-STAT1 by separating the nuclear and cytoplasmic fractions. In DYSF-overexpressing cells, STAT1 was detected in the cytoplasmic extracts regardless of cytokine stimulation when probed with a specific antibody against U-STAT1 (Fig 4E). Moreover, STAT1 was prominently detected in the nuclear fraction of DYSF-overexpressing cells upon IFN-γ stimulation, suggesting that DYSF contributes to IFN-γ–associated STAT1 nuclear accumulation. Conversely, cytoplasmic STAT1 expression was increased in IFN-γ–stimulated control cells. These findings suggest that DYSF enhances not only STAT1 Ser727 phosphorylation but also nuclear accumulation of U-STAT1.

To assess the influence of intracellular Ca^2+^ on STAT1 signaling in macrophages, we treated DYSF-overexpressing or control J774A.1 macrophages with A23187. The abundance of U-STAT1 and phosphorylated STAT1 in DYSF-overexpressing cells increased with A23187 treatment, whereas that in control cells was minimally affected (Fig S4C and D). These results suggest that intracellular Ca^2+^ affects STAT1 protein levels in a manner dependent on DYSF expression. We also assessed the localization of U-STAT1 in DYSF-expressing cells treated with A23187. Immunofluorescence images revealed that A23187 treatment increased nuclear localization of STAT1 in DYSF-overexpressing macrophages (Fig S4E and F), indicating that increased intracellular Ca^2+^ can promote STAT1 nuclear localization in the presence of high DYSF expression.

Next, to determine whether DYSF-induced STAT1 Ser727 phosphorylation depends on Ca^2+^ influx, we treated DYSF-overexpressing J774A.1 cells with the Ca^2+^ entry inhibitors BTP2 and SKF96365 or with Ca^2+^-free/EGTA conditions. BTP2, SKF96365, and Ca^2+^-free/EGTA conditions reduced DYSF-associated STAT1 Ser727 phosphorylation (Fig 4F and G). Consistently, immunofluorescence staining showed that these Ca^2+^-blocking conditions reduced nuclear STAT1 localization in DYSF-overexpressing cells (Fig 4H and I). These results suggest that DYSF-associated STAT1 Ser727 phosphorylation and nuclear localization are dependent, at least in part, on Ca^2+^ influx.

Finally, to determine whether the DYSF–Ca^2+^–STAT1 axis affects downstream inflammatory gene expression, we examined STAT1-responsive genes by qRT-PCR. DYSF overexpression increased the expression of STAT1-responsive genes, including *Cxcl10, Irf1, Nos2/iNOS,* and *Socs1*, with or without IFN-γ stimulation (Fig S4G). Treatment with BTP2 or SKF96365 attenuated the induction of these genes in DYSF-overexpressing cells (Fig 4J). We also confirmed efficient *Stat1* knockdown by immunoblotting (Fig S4H). *Stat1* knockdown reduced DYSF-induced expression of STAT1-responsive genes (Fig 4K). To further assess whether STAT1 contributes to DYSF-induced cytokine output, we measured TNF-α and IL-6 levels in conditioned media after *Stat1* knockdown. *Stat1* knockdown attenuated TNF-α and IL-6 secretion from DYSF-overexpressing macrophages (Fig S4I). These results suggest that DYSF promotes inflammatory macrophage-associated gene expression through Ca^2+^-dependent STAT1 Ser727–associated signaling.

### DYSF accumulates in aged macrophage-lineage cells within skeletal muscle tissue

**Figure S5.**
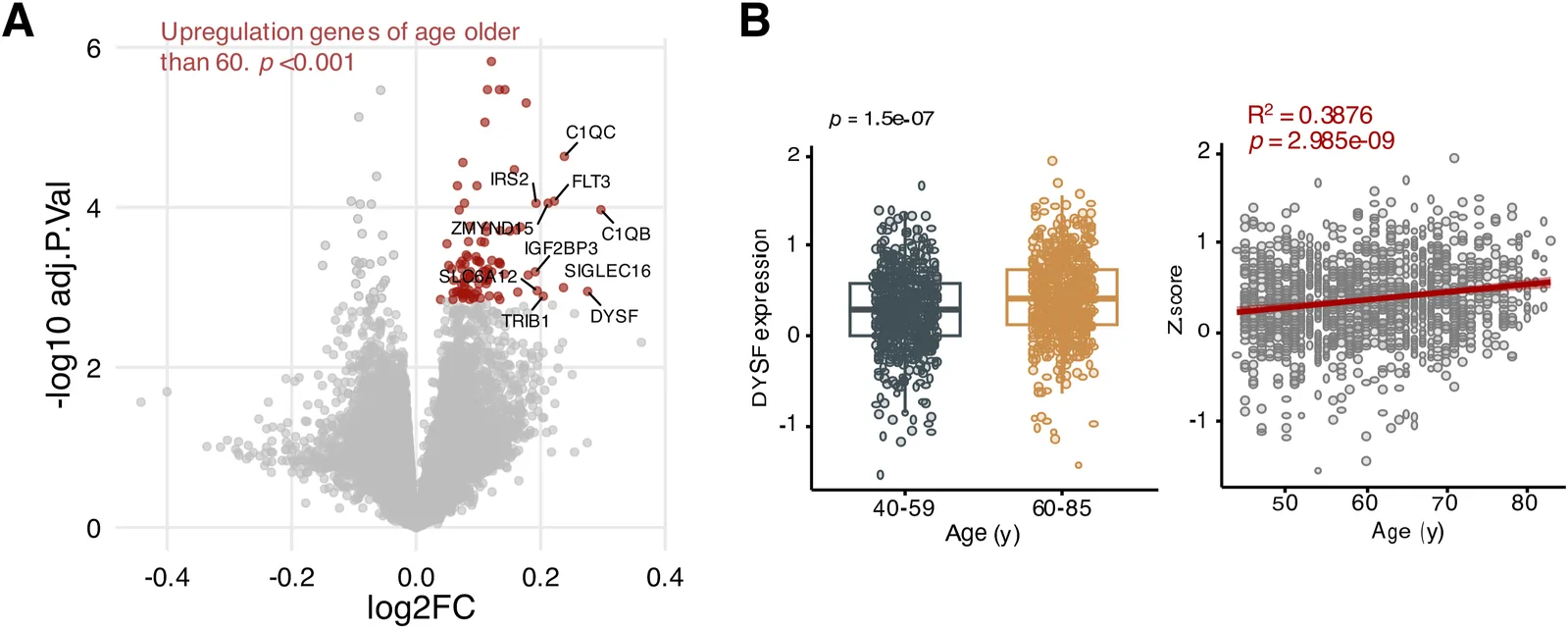
Age-associated increase of DYSF expression in CD14-positive cells from human peripheral blood. **(A)** Volcano plot of transcriptional microarray data from human peripheral CD14^+^ cells (GSE56045; n = 1,202; age range 44–83 yr). **(B)** Left, *DYSF* gene expression in adult (44–59 yr, n = 593) and older (60–83 yr, n = 609) individuals. Right, scatter plot showing the relationship between *DYSF* gene expression and age in human peripheral CD14^+^ monocytes. Each dot represents one individual. The red line indicates linear regression. R^2^ = 0.3876; *P* = 2.985 × 10^−9^.

To investigate DYSF expression in monocyte/macrophage-lineage cells in vivo, we focused on macrophage-lineage cells in steady-state skeletal muscle and investigated their behavior in skeletal muscle in both aged and young mice. We first analyzed gene expression data (GSE56045) (Reynolds et al, 2014) from CD14-positive cells isolated from human peripheral blood to explore the relationship between DYSF expression levels and aging (Fig S5A). Our analysis revealed that *Dysf* mRNA expression levels increased in an age-dependent manner (*P*-value = 2.985 × 10^−9^, R = 0.388) (Fig S5B).

**Figure 5.**
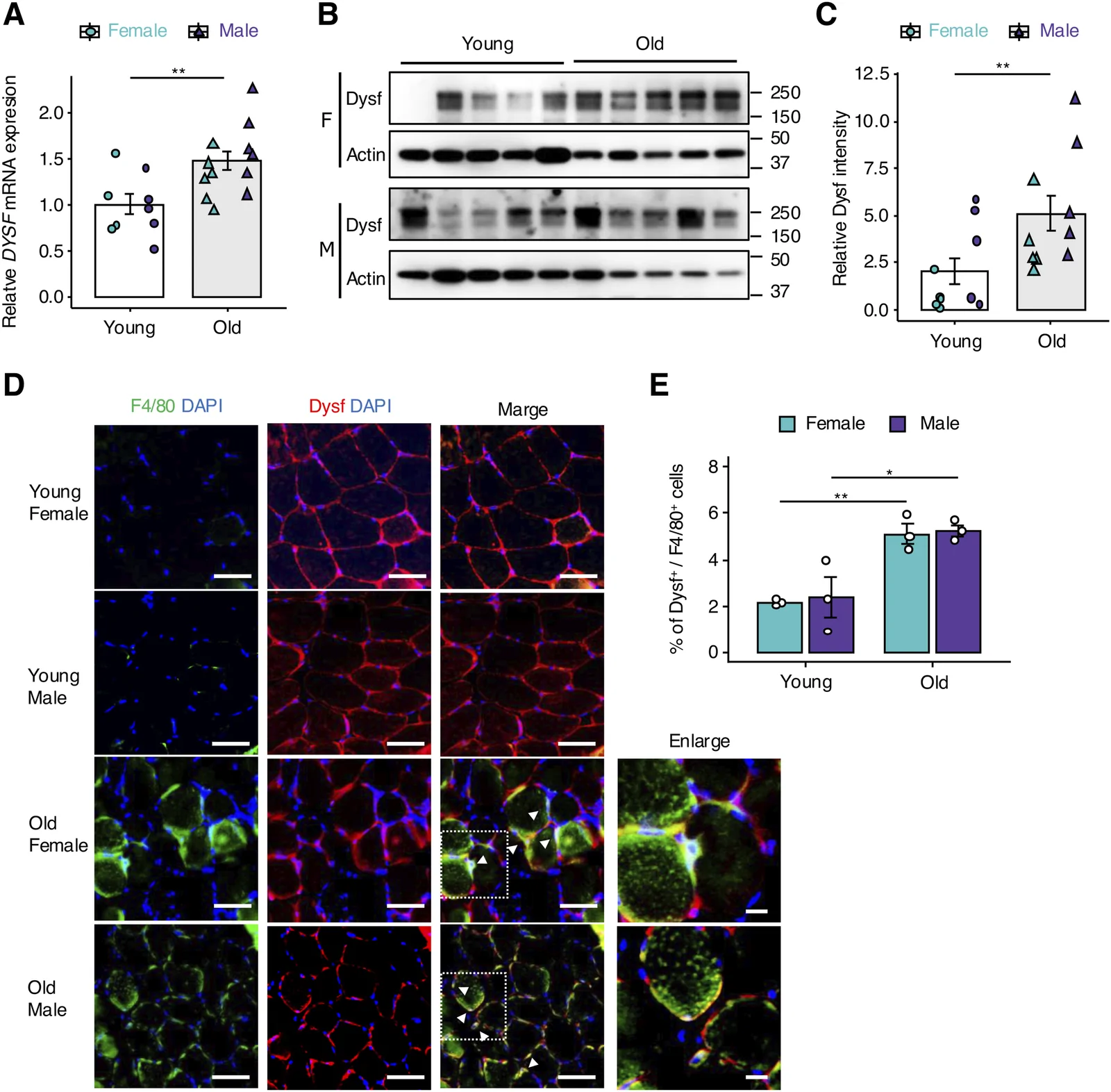
Dysf-expressing F4/80-positive macrophages increase in aged skeletal muscle. **(A)** qRT-PCR analysis of *Dysf* mRNA expression in monocytes from young (6–9 wk) and aged (18–30 mo) mice. Group sizes: young female (n = 5), aged female (n = 5), young male (n = 5), aged male (n = 5). **(B)** Immunoblot analysis of Dysf protein levels in (A). β-actin (Actin) was used as a loading control; F, female; M, male. **(C)** Quantification of Dysf protein abundance normalized to β-actin in (B). Group sizes: young female (n = 5), aged female (n = 5), young male (n = 5), and aged male (n = 5). **(D)** Representative immunofluorescence images of frozen soleus muscle sections from young (6–8 wk) and aged mice (18–30 mo) stained for F4/80 and Dysf; nuclei were counterstained with DAPI. White arrows show DYSF/F4/80-doble positive cells. Scale bar, 50 μm. Detailed view, 20 μm. **(E)** Quantification of the percentage of F4/80-positive and Dysf-positive from five microscopic fields per mouse in young (8–9 wk; n = 3) and aged (18–30 mo; n = 3) mice. Field-level measurements from the same mouse were averaged before statistical analysis, and each mouse was treated as one biological replicate. Each dot represents one mouse. Pre-averaged field-level measurements are provided in the corresponding Source Data file. Data are presented as mean ± SEM. Statistical analysis was performed using an unpaired two-tailed *t* test (A, C) or one-way/two-way ANOVA followed by Tukeyʼs multiple comparisons test (E), as indicated. Only biologically relevant comparisons are shown. *P < 0.05, **P < 0.01. Source data are available for this figure.

To validate these findings, we isolated monocytes from the bone marrow of young (6–8 wk old, 5 females/5 males) and aged (18–30 mo old, 5 females/5 males) mice and cultured monocyte-derived CD11b-positive macrophage-lineage cells. We observed that compared with that in young mice, *Dysf* mRNA expression in CD11b-positive cells was up-regulated in both male and female aged mice (Fig 5A). Furthermore, DYSF protein levels were significantly increased in CD11b-positive cells from aged mice in both males and females (Fig 5B and C), suggesting that aging is associated with increased DYSF expression in monocyte/macrophage-lineage cells. Two-way ANOVA did not reveal a significant age × sex interaction, indicating that the age-associated increase in DYSF expression was observed in both sexes, although larger cohorts will be required to evaluate subtle sex-dependent differences.

**Figure S6.**
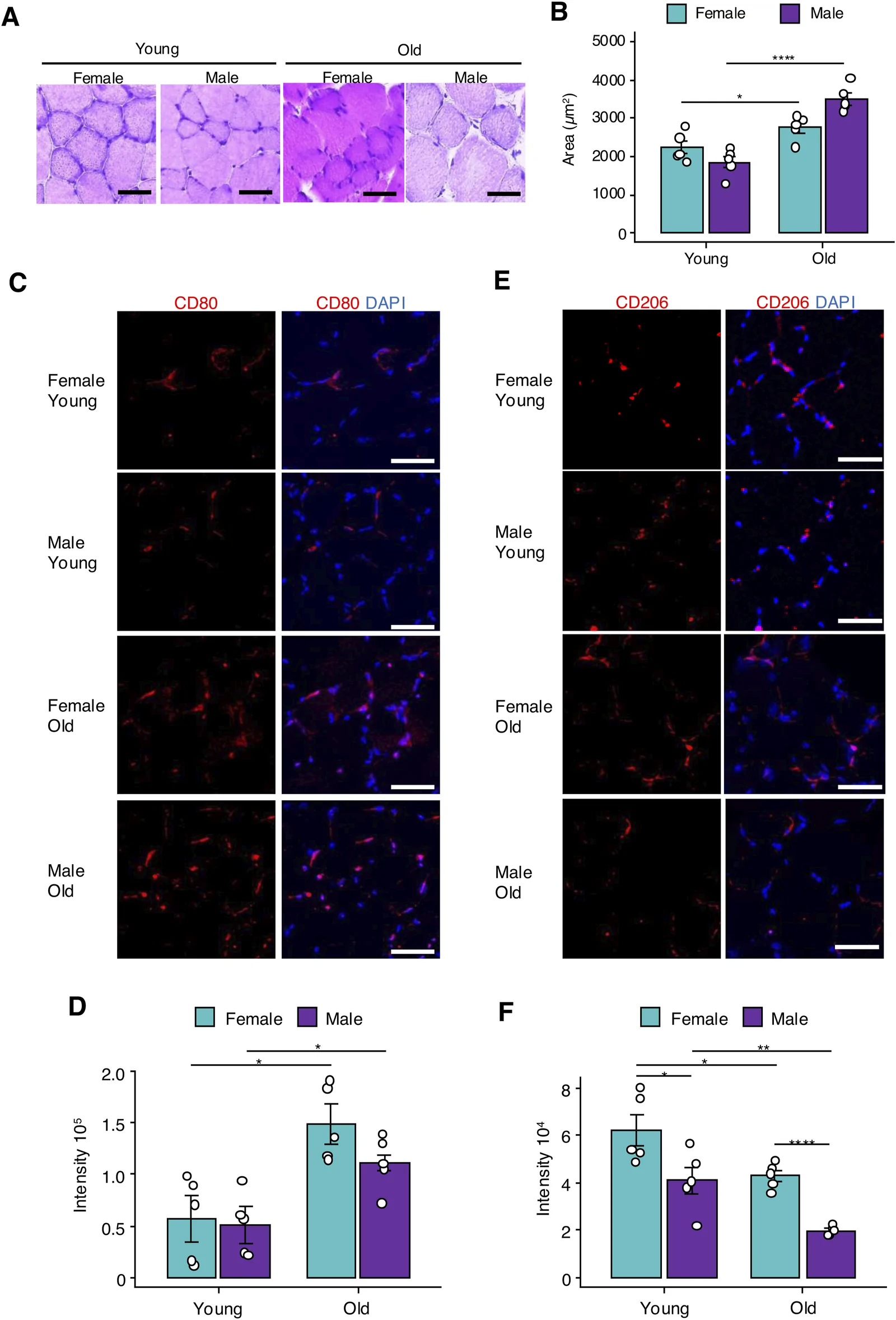
Proinflammatory macrophage infiltration in aged skeletal muscle. **(A)** Hematoxylin and eosin–stained transverse sections of soleus muscle showing muscle morphology. Scale bar, 25 μm. **(B)** Myofiber CSA was quantified from two microscopic fields in each muscle section, with a total of 10 myofibers analyzed per mouse. Values from all analyzed myofibers within the same mouse were averaged before statistical analysis, and each mouse was treated as one biological replicate. Each dot represents one mouse (n = 5 mice per group). Pre-averaged individual myofiber measurements are provided in the corresponding Source Data file. **(C)** Representative images of frozen soleus muscle sections stained with an anti-CD80 antibody to identify M1-type macrophages. Nuclei were counterstained with DAPI. Scale bars, 25 μm. **(D)** Quantification of CD80-positive signal intensity from four microscopic fields per mouse in young (8–9 wk; n = 5) and aged (18–30 mo; n = 5) mice. Field-level measurements from the same mouse were averaged before statistical analysis, and each mouse was treated as one biological replicate. Each dot represents one mouse. Pre-averaged field-level measurements are provided in the corresponding Source Data file. **(E)** Representative images of frozen soleus muscle sections stained with an anti-CD206 antibody to identify M2-type macrophages. Nuclei were counterstained with DAPI. Scale bars, 25 μm. **(F)** Quantification of CD206-positive signal intensity from four microscopic fields per mouse in young (8–9 wk; n = 5) and aged (18–30 mo; n = 5) mice. Field-level measurements from the same mouse were averaged before statistical analysis, and each mouse was treated as one biological replicate. Each dot represents one mouse. Pre-averaged field-level measurements are provided in the corresponding Source Data file. Data are presented as mean ± SEM. Statistical analysis was performed using a one-way/two-way ANOVA followed by Tukeyʼs multiple comparisons test for multiple-group comparisons, as indicated. Only biologically relevant comparisons are shown. *P < 0.05, **P < 0.01, ****P < 0.0001. Source data are available for this figure.

Next, histological analyses were performed to assess age-associated changes in skeletal muscle tissue (Fig S6A). Myofiber cross-sectional area (CSA) was increased in aged skeletal muscle in both male and female mice, with no clear sex-specific difference (Fig S6B). Although an apparent increase in CSA was observed with aging, this finding should be interpreted cautiously because aged skeletal muscle also showed increased interstitial spaces, intramuscular fat, and connective tissue-like changes. These histological observations indicate that aged skeletal muscle exhibits structural alterations consistent with age-associated tissue remodeling.

We then assessed inflammatory and alternative macrophage-associated markers in skeletal muscle sections by immunofluorescence. Immunostaining for CD80 revealed increased accumulation of CD80-positive cells in aged skeletal muscle (Fig S6C and D). Conversely, only minor differences in CD206-positive cells were observed between young and aged skeletal muscle (Fig S6E and F). Because CD80 and CD206 are not specific to macrophages in skeletal muscle sections, these data were interpreted as tissue-level marker immunoreactivity rather than definitive identification of macrophage subsets.

**Figure S7.**
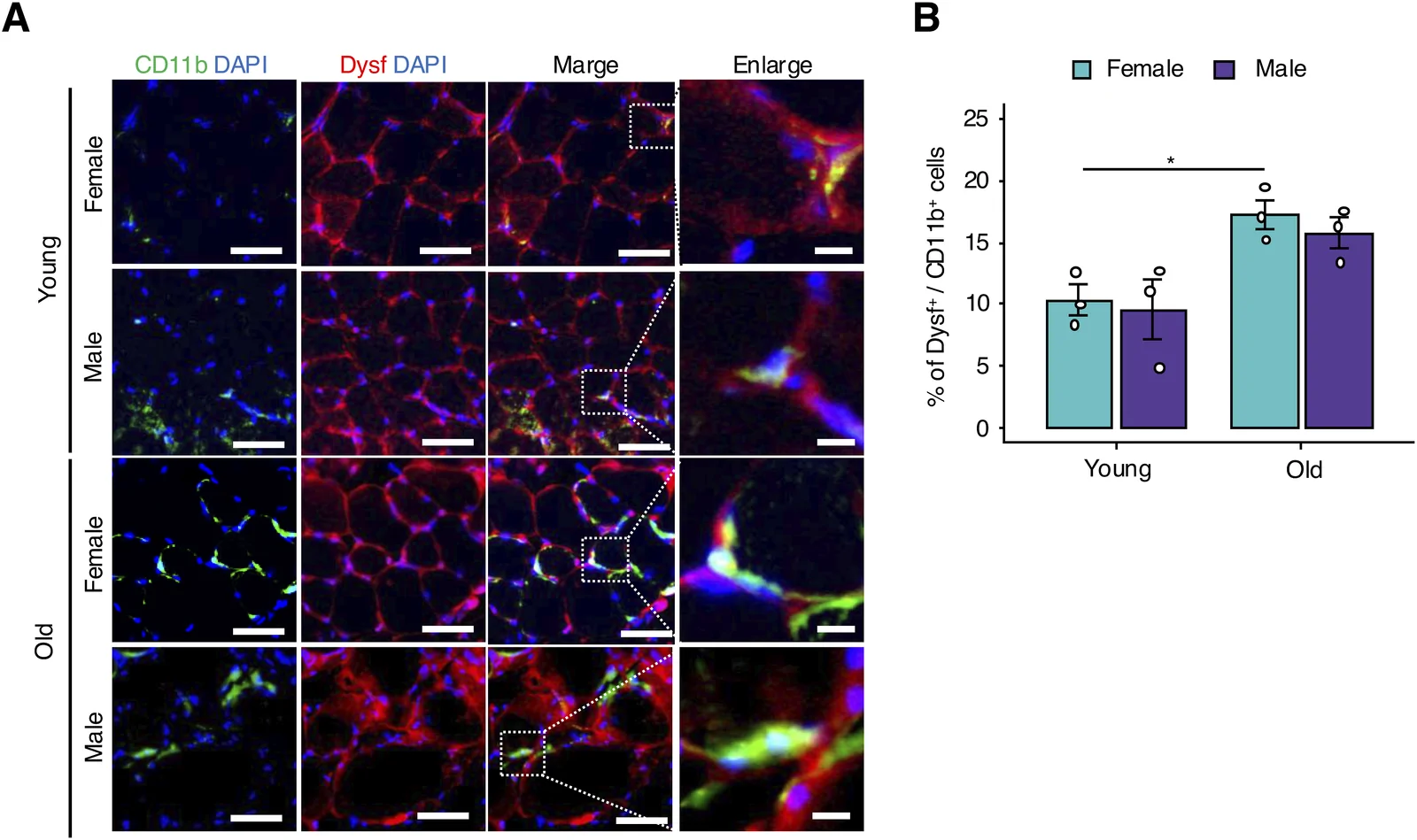
CD11b^+^DYSF^+^ myeloid cells increase in aged skeletal muscle. **(A)** Representative immunofluorescence images of frozen soleus muscle sections from young (8 wk) and old (30 mo) mice stained for CD11b and Dysf; nuclei were counterstained with DAPI. Scale bar, 50 μm. Detailed view, 20 μm. **(B)** Quantification of the percentage of CD11b-positive and Dysf-positive from four microscopic fields per mouse in young (8–9 wk; n = 5) and aged (18–30 mo; n = 5) mice. Field-level measurements from the same mouse were averaged before statistical analysis, and each mouse was treated as one biological replicate. Each dot represents one mouse. Pre-averaged field-level measurements are provided in the corresponding Source Data file. Data are presented as mean ± SEM. Statistical significance was determined by one-way ANOVA with Tukeyʼs post hoc test. Only biologically relevant comparisons are shown. *P < 0.05. Source data are available for this figure.

To more directly identify Dysf-expressing macrophage-lineage cells in skeletal muscle, we performed co-immunostaining for F4/80 and Dysf. F4/80-positive cells were rarely detected in young skeletal muscle but were increased in aged skeletal muscle. Importantly, F4/80^+^/Dysf^+^ cells were more frequently observed in aged mice than in young mice (Fig 5D and E). No clear difference was observed between males and females within either age-group. Consistent with this finding, co-immunostaining for CD11b and Dysf also showed increased CD11b^+^/Dysf^+^ cells in aged skeletal muscle (Fig S7A and B). These findings indicate that Dysf-expressing macrophage-lineage cells accumulate in aged skeletal muscle and support the association between aging, macrophage-lineage cell accumulation, and increased Dysf expression in skeletal muscle tissue.

### DYSF-overexpressing macrophages suppress myotube fusion through soluble inflammatory mediators

**Figure 6.**
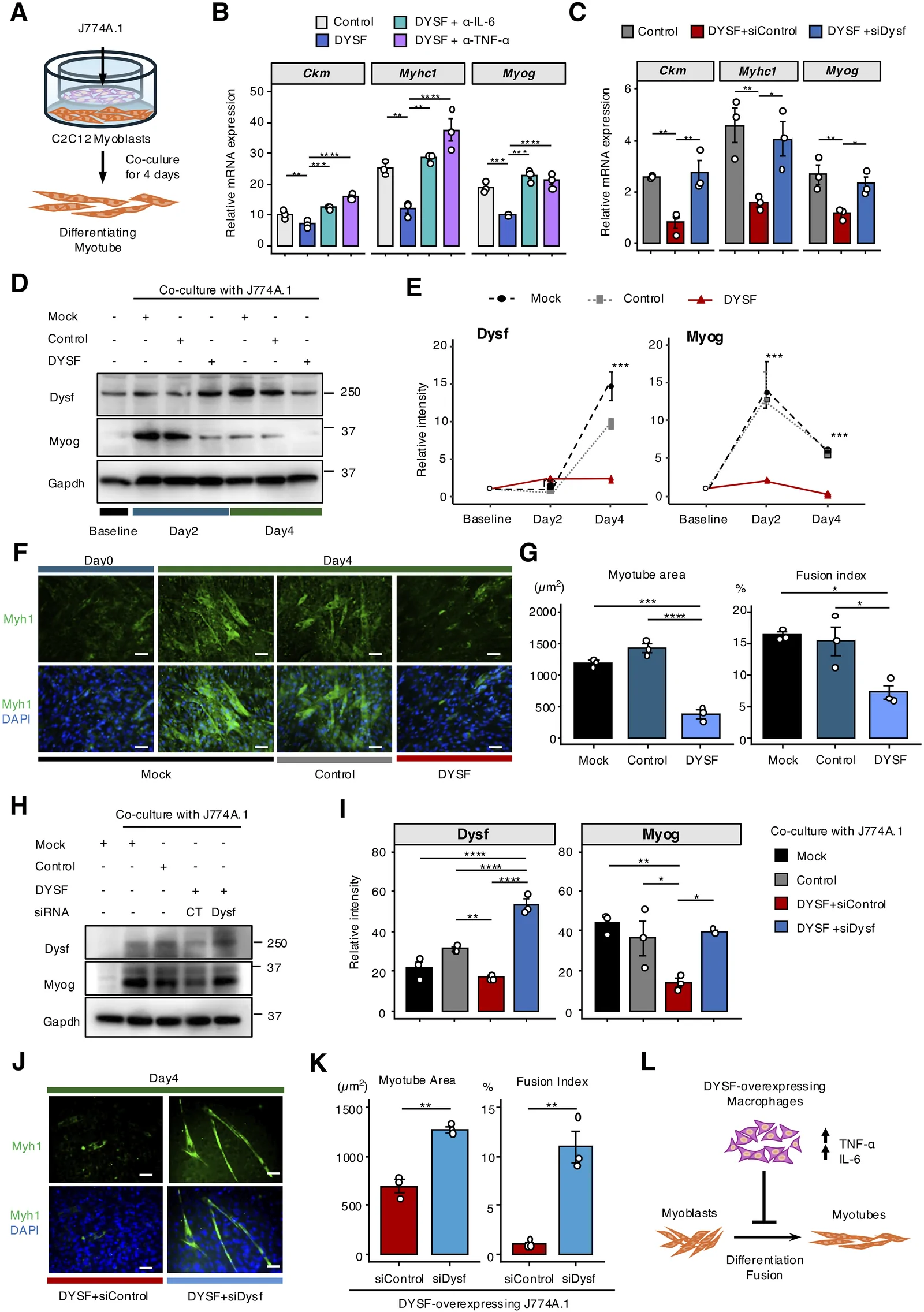
DYSF-overexpressing macrophages suppress myotube fusion. **(A)** Schematic of the macrophage–myoblast co-culture system. **(B)** qRT-PCR analysis of myogenic differentiation markers, *Ckm, Myhc, and Myog*, in C2C12 myotubes after co-culture with J774A.1 macrophages transfected with DYSF expression vector (DYSF) or empty control vector (Control) and treated with or without neutralizing antibodies against TNF-α (α-TNF-α, 1 μg/ml) or IL-6 (α-IL-6, 1 μg/ml). Each dot represents one independent co-culture (n = 3). **(C)** qRT-PCR analysis of myogenic differentiation markers in C2C12 myotubes after co-culture with J774A.1 macrophages transfected with DYSF expression vector plus *Dysf* siRNA or control siRNA. Each dot represents one independent co-culture (n = 3). **(D)** Immunoblot analysis of differentiated C2C12 lysates after co-culture with J774A.1 macrophages transfected with DYSF expression vector or empty control vector probed for Dysf and myogenin (Myog). GAPDH was used as a loading control. **(E)** Quantification of Dysf and Myog protein levels in C2C12 cells after co-culture in (D). Each dot represents one independent co-culture (n = 3). **(F)** Representative immunofluorescence images of Myh1 in C2C12 cells after co-culture with J774A.1 macrophages transfected with DYSF expression vector or empty control vector. Scale bar, 50 μm. **(G)** (Left) Quantification of Myh1^+^ myotube area from five microscopic fields per independent co-culture 4 d after differentiation in (F). (Right) Fusion index of Myh1^+^ myotubes, calculated as the percentage of nuclei within multinucleated myotubes (>3 nuclei). For both analyses, field-level measurements were averaged within each independent co-culture before statistical analysis. Each dot represents one independent co-culture (n = 3 biological replicates). Pre-averaged field-level measurements are provided in the corresponding Source Data file. **(H)** Immunoblot analysis of differentiated C2C12 lysates after co-culture with J774A.1 macrophages transfected with DYSF expression vector plus *Dysf* siRNA or control siRNA, probed for DYSF and Myog. GAPDH was used as a loading control. CT, Control. **(I)** Quantification of Dysf and Myog protein levels in C2C12 cells after 4 d of co-culture in (H). Each dot represents one independent co-culture (n = 3). **(J)** Representative immunofluorescence images of Myh1 in C2C12 cells after 4 d of co-culture with J774A.1 macrophages transfected with DYSF expression vector plus *Dysf* siRNA or control siRNA. Scale bar, 50 μm. **(K)** (Left) Quantification of Myh1^+^ myotube area from five microscopic fields per independent co-culture 4 d after differentiation in (F). (Right) Fusion index of Myh1^+^ myotubes, calculated as the percentage of nuclei within multinucleated myotubes (>3 nuclei). For both analyses, field-level measurements were averaged within each independent co-culture before statistical analysis. Each dot represents one independent co-culture (n = 3 biological replicates). Pre-averaged field-level measurements are provided in the corresponding Source Data file. **(L)** Proposed model illustrating Ca^2+^-dependent STAT1 signaling in DYSF-overexpressing macrophages and its inhibitory effect on myoblast differentiation through soluble inflammatory mediators. Data are presented as mean ± SEM. Each dot represents one independent experiment or independent co-culture, unless otherwise indicated. Statistical analysis was performed using one-way/two-way ANOVA followed by Tukeyʼs multiple comparisons test (A, C, E, G, I) or an unpaired two-tailed *t* test (K), as indicated. *P < 0.05, **P < 0.01, ***P < 0.001, ****P < 0.0001. Source data are available for this figure.

Macrophages are integral to skeletal muscle repair and regeneration, a process vital for muscle regeneration and repair (Kharraz et al, 2013). Inflammatory macrophages are involved in regulating inflammation, phagocytosis, and the recruitment and activation of muscle stem cells, also known as satellite cells. To elucidate the impact of DYSF-regulated inflammatory macrophage–like activation on muscle fiber formation, we co-cultured mouse monocyte/macrophage J774A.1 cells with mouse myoblast C2C12 using a Transwell chamber insert system (Fig 6A). DYSF-overexpressing or control vector–transfected macrophages were cultured in the upper well of a Transwell chamber insert, whereas C2C12 cells were seeded in the lower well. For myoblast differentiation and fusion, cells were cultured in medium containing 2% horse serum, and myoblast differentiation into myotubes was monitored over time. Because macrophages and myoblasts were physically separated by the insert, this system allowed us to assess the effects of soluble factors released from macrophages on myogenic differentiation.

We next used qRT-PCR to measure the mRNA expression levels of *creatine kinase (Ckm), myosin heavy chain (Myhc)*, and *Myog* to assess myotube formation after co-culture. We found that these genes were up-regulated after differentiation in control co-cultures, whereas their expression was inhibited in C2C12 cells co-cultured with DYSF-overexpressing macrophages (Fig 6B). Notably, neutralization of TNF-α or IL-6 restored the expression of all three genes in the co-cultured C2C12 cells. These findings suggest that proinflammatory cytokines released from DYSF-overexpressing macrophages contribute to the suppression of myogenic differentiation.

To further determine whether endogenous Dysf in macrophages contributes to the suppression of myogenic differentiation, we performed co-culture experiments using J774A.1 cells transfected with *Dysf* siRNA or control siRNA. Compared with control siRNA–transfected macrophages, *Dysf-*knockdown macrophages showed a reduced inhibitory effect on C2C12 differentiation, as indicated by restored expression of myogenic differentiation markers (Fig 6C). These loss-of-function data support the involvement of macrophage DYSF in the regulation of myogenic differentiation.

Myofiber formation was assessed by immunoblot analysis and immunohistochemistry using muscle differentiation markers, Myh1, Dysf, and Myog. In the control co-culture, differentiation induction led to increased expression of Dysf and Myog, whereas the expression of these proteins was suppressed in C2C12 cells co-cultured with DYSF-overexpressing macrophages (Fig 6D and E). Furthermore, immunofluorescence staining for Myh1 demonstrated that differentiation induction increased both expression and enhanced myotube formation in the control co-culture, whereas fusion was suppressed in the DYSF-overexpressing macrophage co-culture, and quantification confirmed reduced Myh1-positive myotube area (Fig 6F) and a lower fusion index (Fig 6G). Conversely, immunoblot analysis showed that co-culture with DYSF-overexpressing macrophages transfected with *Dysf* siRNA restored DYSF and Myog protein expression in differentiated C2C12 cells compared with co-culture with DYSF-overexpressing macrophages transfected with control siRNA (Fig 6H and I). Consistently, immunofluorescence analysis showed that *Dysf* knockdown in DYSF-overexpressing macrophages restored Myh1-positive myotube formation (Fig 6J). Quantification of Myh1-positive myotube area and the fusion index further showed that *Dysf* knockdown in DYSF-overexpressing macrophages improved myotube formation and fusion (Fig 6K). Together, these gain- and loss-of-function data indicate that macrophage DYSF contributes to the suppression of myogenic differentiation and myotube fusion, at least in part through soluble inflammatory mediators such as TNF-α and IL-6 (Fig 6L).

## Discussion

In this study, we identify DYSF as a previously unrecognized regulator of inflammatory macrophage–like activation associated with Ca^2+^-dependent STAT1 signaling. In macrophage–myoblast co-culture systems, DYSF-enriched inflammatory macrophages suppressed myogenic differentiation, linking macrophage programming to functional outcomes in neighboring muscle cells. Notably, elevated DYSF expression was observed in human CD14-positive cells from aged donors and mouse monocyte/macrophage-lineage cells from aged mice. DYSF-expressing F4/80-positive macrophage-lineage cells accumulated in aged skeletal muscle in both males and females. However, these aged-mouse findings are associative and do not establish that macrophage DYSF causally contributes to age-associated muscle alterations in vivo. Together, our gain- and loss-of-function analyses support a model in which macrophage DYSF enhances inflammatory macrophage-associated responses and contributes, at least in part, to the suppression of myotube formation through soluble inflammatory mediators in co-culture systems.

Our findings support a role for DYSF in regulating macrophage activation in association with Ca^2+^ signaling. The influx of Ca^2+^ is crucial for macrophage activation, as elevated intracellular Ca^2+^ levels regulate cytokine production and support associated inflammatory signaling pathways. The transition between inflammatory and reparative macrophage states is intricate and cannot be fully explained by a simple M1/M2 dichotomy. In this study, IFN-γ and IL-4 were used as defined in vitro stimuli to examine DYSF-dependent inflammatory and alternative macrophage-associated responses, respectively. These conditions do not fully recapitulate the dynamic macrophage states observed during muscle regeneration in vivo*.* Therefore, we refer to DYSF-associated macrophage responses as inflammatory macrophage–like activation or inflammatory macrophage-associated features, rather than defining them strictly as fixed M1 or M2 macrophage states.

The balance among STAT1-, STAT3-, and STAT6-associated signaling pathways plays an important role in regulating macrophage activation programs (Sica & Mantovani, 2012). Inflammatory macrophage-associated activation is generally linked to STAT1 and NF-κB signaling, whereas STAT3 and STAT6 are more closely associated with anti-inflammatory or alternative macrophage-associated responses. In our experimental setting, DYSF preferentially enhanced STAT1 Ser727–associated signaling, whereas STAT3, STAT6, and NF-κB p65 phosphorylation were not markedly altered. These findings suggest that DYSF is preferentially associated with STAT1 Ser727–related inflammatory signaling under the conditions tested.

In this study, we demonstrated that DYSF positively influences STAT1 abundance, Ser727 phosphorylation, and nuclear localization in macrophages. STAT1 is primarily activated by interferons, such as IFN-α/β and IFN-γ, as well as by IL-6 (Tolomeo et al, 2022). Upon activation, JAK kinases phosphorylate STAT1, which then dimerizes, translocates to the nucleus, and regulates transcription. Our data indicate that DYSF overexpression preferentially enhanced STAT1 Ser727 phosphorylation without markedly increasing canonical IFN-γ–dependent STAT1 Tyr701 phosphorylation, whereas *Dysf* knockdown reduced both STAT1 Tyr701 and Ser727 phosphorylation under IFN-γ–stimulated conditions. These findings suggest that DYSF contributes to STAT1 signaling through both regulation of endogenous STAT1 abundance and enhancement of STAT1 Ser727–associated signaling. Importantly, DYSF overexpression did not markedly alter STAT3, STAT6, or NF-κB p65 phosphorylation under the same conditions, supporting a preferential association of DYSF with STAT1-associated signaling in this experimental setting.

Ca^2+^ influx may indirectly modulate STAT1 activity. Calcium-calmodulin–dependent protein kinase II (CaMKII) and Ca^2+^-dependent kinases can increase STAT1 phosphorylation (Jayasree et al, 2002). Consequently, intracellular Ca^2+^ may serve as a fine-tuner of STAT1 strength and duration. In macrophages, STAT1 signaling promotes the expression of proinflammatory cytokines that affect store-operated Ca^2+^ entry (SOCE) and ER–mitochondria Ca^2+^ transfer, thereby supporting inflammatory macrophage activation (Nascimento Da Conceicao et al, 2021). Consistent with this concept, DYSF overexpression enhanced intracellular Ca^2+^ responses, whereas *Dysf *knockdown attenuated Ca^2+^ influx. Moreover, Ca^2+^ entry inhibition by BTP2, SKF96365, or Ca^2+^-free/EGTA conditions reduced DYSF-associated STAT1 Ser727 phosphorylation and STAT1 nuclear localization. These results support the idea that DYSF-associated STAT1 signaling depends, at least in part, on Ca^2+^ influx. Because A23187 is a calcium ionophore, these experiments indicate that increased intracellular Ca^2+^ is sufficient to enhance STAT1-associated signaling in DYSF-overexpressing macrophages, but they do not by themselves establish a physiological receptor-mediated Ca^2+^ pathway. Further studies using receptor-mediated stimulation will be important to define how DYSF regulates physiological Ca^2+^ signaling in macrophages. Furthermore, sustained cytosolic Ca^2+^ signaling is known to activate inflammatory pathways and promote the production of proinflammatory cytokines such as TNF-α and IL-6. DYSF-associated changes in Ca^2+^ signaling may contribute to inflammatory macrophage responses. In support of this model, DYSF overexpression increased the expression of STAT1-responsive inflammatory genes, including *Cxcl10, Irf1, Nos2/iNOS*, *and Socs1*, and this induction was attenuated by Ca^2+^ entry inhibitors or *STAT1* knockdown. These findings link DYSF-associated Ca^2+^ influx and STAT1 signaling to inflammatory gene expression in macrophages.

Macrophages play essential roles in coordinating myogenic progression through dynamic changes in inflammatory signaling. Proinflammatory macrophages support early myoblast activation, whereas a timely transition toward anti-inflammatory or reparative states facilitates differentiation and tissue remodeling. Disruption of this balance results in impaired myogenic differentiation. In this study, we demonstrate that DYSF-enriched inflammatory macrophages suppress myoblast fusion in vitro, indicating that excessive or sustained inflammatory macrophage programming can negatively impact myogenic capacity. These findings suggest that DYSF-dependent regulation of macrophage activation may influence muscle cell behavior through paracrine inflammatory signaling. Because macrophages and myoblasts were physically separated in the Transwell co-culture system, the inhibitory effect of DYSF-overexpressing macrophages on myogenic differentiation is likely mediated by soluble factors rather than direct cell–cell contact. Consistent with this interpretation, neutralization of TNF-α or IL-6 partially restored the expression of myogenic differentiation markers. Furthermore, *Dysf *knockdown in DYSF-overexpressing macrophages restored myogenic marker expression and Myh1-positive myotube formation, providing loss-of-function evidence that macrophage DYSF contributes to the suppression of myogenic differentiation and myotube fusion.

Although DYSF is classically associated with muscle membrane repair, our findings uncover a distinct role for DYSF in macrophage biology. Our findings support a functional link between DYSF, Ca^2+^ signaling, STAT1-associated inflammatory responses, and macrophage–muscle cell communication. However, several limitations should be noted. First, although our aged-mouse tissue analyses demonstrate the accumulation of DYSF-expressing F4/80-positive macrophage-lineage cells in skeletal muscle, they do not establish in vivo causality. Thus, the physiological and causal relevance of macrophage DYSF in skeletal muscle aging remains to be demonstrated. Macrophage-specific *Dysf* loss-of-function models, bone marrow transplantation, and muscle injury or aging models will be required to determine whether macrophage DYSF directly contributes to impaired muscle regeneration in vivo. In addition, some histological analyses were based on a limited number of animals, restricting statistical power. The apparent increase in CSA observed in the aged cohort differs from the reduction in myofiber size commonly associated with aging and may reflect differences in muscle type, fiber-type composition, age-associated tissue heterogeneity, and sampling. Because the CSA analysis was based on a limited number of fields and fibers, the apparent increase in CSA should be interpreted cautiously and may not fully reflect the overall distribution of myofiber size in aged muscle. Furthermore, the cytokine-defined macrophage states used in vitro do not fully recapitulate macrophage heterogeneity in skeletal muscle, and the distribution of DYSF among macrophage subpopulations in vivo remains to be established. Second, although DYSF gain-of-function experiments helped test whether increased DYSF abundance promotes inflammatory macrophage-associated responses, overexpression of a large membrane-associated protein may introduce nonphysiological effects. We therefore integrated *Dysf* loss-of-function experiments across inflammatory gene expression, cytokine secretion, Ca^2+^ responses, STAT1 phosphorylation, migration, and macrophage–myoblast co-culture assays. Nevertheless, validation in primary macrophages, including BMDMs from wild-type and Dysf-deficient mice, will be required to confirm the role of endogenous DYSF under more physiological conditions. Third, our Ca^2+^ imaging experiments primarily used ionophore- and SOCE-based approaches, which do not fully recapitulate physiological receptor-mediated Ca^2+^ signaling. Moreover, although the Ca^2+^ inhibition and STAT1 loss-of-function experiments support a functional relationship between DYSF-dependent Ca^2+^ signaling and STAT1 activation, they do not establish a strictly linear or exclusive DYSF-Ca^2+^-STAT1 pathway. Further studies using primary macrophages and physiological stimuli will be required to define this mechanism. Finally, TNF-α and IL-6 neutralization only partially restored myogenic differentiation, suggesting that additional macrophage-derived factors may contribute. The use of a Transwell system and cell lines also limits conclusions regarding direct cell–cell interactions and physiological relevance, which should be addressed in future studies using primary macrophages and myogenic cells.

## Materials and Methods

### Animals

Mouse experiments were performed under protocols approved by the Yamaguchi University Animal Care and Use Committee (No.80-002 and No.80-021). Male and female were used in this study, and sex-specific features of age were analyzed independently. Mice were group-housed in ventilated cages with a constant temperature of 25°C, 30–70% humidity and a 12-h light/dark cycle. Water and the PicoLab Rodent Diet 20 (LaboDiet) were provided ad libitum.

At the time of bone marrow collection, young mice were 6–9 wk of age and old mice were 18–30 mo of age. After euthanasia, bone marrow samples from limbs were centrifuged (10,000*g*) for 30 s at 4°C and collected bone marrow cells according to a protocol (Gamrekelashvili et al, 2021). To isolate mouse monocytes/macrophages, cells obtained from bone marrow samples were incubated with a mixture of Monocyte Biotin-Antibody Cocktail (130-100-629; Miltenyi Biotec) and separated using the MACS system (Miltenyi Biotec) according to the manufacturerʼs instructions.

Isolated monocytes were cultured in high-glucose Dulbecco’s Modified Eagle Medium (DMEM; 08459-64; Nacalai Tesque) containing 10% fetal bovine serum (FBS; 173012-500ML; Nichirei), 200 mM GlutaMAX (3505006; Invitrogen), 5 ng/ml recombinant mouse GM-CSF (576302; BioLegend), 2.5 ng/ml recombinant mouse IL-4 (574304; BioLegend), 100 U/ml penicillin, and 100 μg/ml streptomycin (P/S; 26253-84; Nacalai Tesque) at a non-treated surface plate in a humidified atmosphere at 37°C in 5% CO_2_. The culture medium was changed every 2 d. To isolated monocyte-derived CD11b-positive macrophages, cultured cells were incubated with a biotin-conjugated antibody against CD11b (558013; BD Biosciences) for 30 min at 4°C and isolated by BD IMag Cell Separation System (BD Biosciences) according to the manufacturerʼs instructions.

### Cell culture

J774A.1 and U937 were kindly provided by the Yamaguchi University Center for Gene Research, and C2C12 (CRL-1772) were purchased from the American Type Culture Collection.

J774A.1, a murine monocyte/macrophage cell line was cultured in high-glucose DMEM containing 10% FBS and 1% P/S. Cell lines were cultured in a humidified atmosphere at 37°C in 5% CO_2_, and the culture medium was changed every 2 d. When cells reached subconfluence, they were rinsed in phosphate-buffered saline (PBS; pH 7.4; Nacalai Tesque) and dislodged the cells using a cell scraper for passage. For macrophage activation, cells were seeded in six-well plates at 6 × 10^5^ cells per well and treated with 100 ng/ml recombinant mouse IFN-γ (575304; BioLegend) or 100 ng/ml recombinant mouse IL-4 for 24 h.

U937, a human pro-monocytic model cell line was cultured in a T75 flask in RPMI 1640 medium without phenol red (RPMI; 18702705; FUJIFILM Wako Pure Chemical) containing 10% FBS and 1% P/S. Cells were passaged for a maximum of 4 wk. For differentiation to macrophage, cells were seeded in six-well plates at 1 × 10^6^ cells per well, then treated with 10 nM phorbol-12-myris-tate-13-acetate (PMA; 162-23591; FUJIFILM Wako Pure Chemical) for 24 or 48 h. For macrophage activation, we add 100 ng/ml recombinant human IFN-γ (570204; BioLegend) or 100 ng/ml recombinant human IL-4 (574004; BioLegend) for 24 h.

C2C12, a mouse myoblast cell line was cultured in high-glucose DMEM containing 10% FBS and 1% P/S. For studies of myogenic differentiation, when cells were reached at full confluency, the culture medium was substituted with differentiated medium contained with DMEM with 2% horse serum (26050088; GIBCO) and 1% P/S. Differentiated medium was changed every day. To neutralize cytokines during myoblast differentiation, we used neutralizing antibodies against mouse TNF-α (17590-1-AP; Proteintech), mouse IL-6 (MAB406-SP; R&D systems).

Cell morphology was analyzed using Fiji/ImageJ. Individual cell boundaries were defined, and cell area, aspect ratio, and circularity were determined using the Shape Descriptors function. Measurements from individual cells within each independent culture were averaged before statistical analysis. Each independent culture was treated as one biological replicate. Image acquisition and quantification were identical acquisition settings and analysis criteria were applied across all experimental groups.

### Cell proliferation assay

J774A.1 cells were seeded in 96-well plates at 10,000 cells per well. After incubated for 24, 48, and 72 h, cell proliferation was measured using CellTiter 96 AQueous One Solution Cell Proliferation Assay (MTS) (G3580; Promega) according to the manufacturerʼs specifications.

### Small interfering RNA

We used Silencer Select Mouse DYSF (Assay ID s77352) and Mouse STAT1 (Assay ID s74444) purchased from Invitrogen. J774A.1 cells were seeded at a density of 1 × 10^5^ cells per well into a six-well plate and incubated with siRNAs (15 nM) and Lipofectamine RNAiMAX transfection reagent (13778075; Invitrogen) according to the manufacturerʼs protocol. The control was Silencer Select Negative Control No. 1 siRNA. siRNA-transfected cells were incubated at 37°C in 5% CO_2_ until the assay. Data are presented as mean from three or four independent experiments, with each dot representing one independent experiment.

### Transient transfection with plasmids

DYSF-3HA was a gift from Steven Vogel (#29767; Addgene plasmid; http://n2t.net/addgene:29767; RRID: Addgene_29767) (Covian-Nares et al, 2010). The control was a pcDNA3.1 vector. For transient transfection of plasmids, J774A.1 cells were seeded at a density of 6 × 10^5^ cells per well into a six-well plate. After incubation for 24 h, the plasmids (10 μg) and Hilymax (H357; Dojindo) in culture medium were added to each well. After 4-h incubation, we changed culture medium and incubated until the experiment is performed.

### In vitro migration assay

1 × 10^5^ of plasmid-transfected J774A.1were seeded on Matrigel (354234; Corning)-coated Transwell insert (pore size, 5.0 μm) in serum-free medium. After 24 h, the cells attached to the lower side were counted. The invading cells in four randomly fields were counted, and the mean of counted cell were reported.

### Intracellular calcium level observation

The Ca^2+^ levels were determined with the use of Calcium Kit II-Fluo 2 (CS33; Dojindo). Control vector or DYSF vector–transfected J774A.1 cells were plated at 4 × 10^4^ cells per well into the 96-well plates for 24 h. After incubation, cells were incubated with 4 μg/ml Fluo-2 AM with 5% Pluronic F-127 and 250 mmol/L Probenecid at 37°C in 5% CO_2_ for 1 h.

To inhibit Ca^2+^ influx, the Ca^2+^ entry inhibitors YM-58483 (BTP2; S8380; Selleckchem) or SKF96365 (S7999; Selleckchem) was added at 5 or 10 μM 3 h before measurement. To evaluate the internal stores of calcium, we added A23187 (C7522; Sigma-Aldrich) at 0.5 or 2 μM as a calcium ionophore, or thapsigargin (209-17281; FUJIFILM Wako Pure Chemical) at 2 μM as a SERCA pump blocker during the measurement. Cells measurement was performed on a Flexstaion 3 (Molecular Devices) with an excitation wavelength of 340/510 nm and an emission wavelength of 380/510 nm. Baseline signals (F0) were recorded 2 min before the addition of each stimulus. Subsequently, continuous fluorescence measurements were performed for 20 min each 25 s. Results are shown as F340/F380 ratios after background subtraction, where F was the fluorescence signal intensity and F0 was the baseline intensity, as calculated by the average of five frames before stimulus application.

### Nuclear and cytoplasmic protein extraction

To separate nuclear and cytoplasmic protein extraction from cells, the cell fractionation was done following a previous protocol with modifications (Schreiber et al, 1989). Briefly, a total of 1 × 10^6^ J774A.1 cells were collected and washed with PBS. To collect the cytosolic fraction, the pellets were resuspended in cold lysis buffer containing with 10 mM HEPES (GB10; Dojindo), 1.5 mM MgCl_2_ (M8266; Sigma-Aldrich), 10 mM KCl (24-4290; Sigma-Aldrich), 0.1 mM EDTA (06894-85; pH 8.0; Nacalai Tesque), 0.1% NP-40 (85124; Thermo Fisher Scientific), a phosphatase inhibitor cocktail (160-24371; FUJIFILM Wako Pure Chemical), and a protease inhibitor (5892791001; Roche). After centrifugation, the nuclear pellets were resuspended in cold buffer containing 20 mM HEPES, 1.5 mM MgCl_2_, 400 nM NaCl (191-01665; FUJIFILM Wako Pure Chemical), 0.1 mM EDTA, and 10% glycerol (075-00616; FUJIFILM Wako Pure Chemical) and homogenate with the 27G needle. After centrifugation, we collected the supernatant as the nuclear extract.

### Immunoblot analysis

The cultured cells in six-well plates were washed with PBS and then lysed with lysis buffer containing M-PER Mammalian Protein Extraction Reagent (78501; Thermo Fisher Scientific) with a phosphatase inhibitor cocktail and a protease inhibitor. The proteins were separated by 10% SDS–PAGE and transferred to polyvinylidene fluoride (PVDF) membranes. The membranes were blocked with Block Ace (non-fat skim milk; UK-B80; BioRad) in distilled water at room temperature for 30 min. The PVDF membranes were incubated with primary antibodies at room temperature for 1 h. The primary antibodies used in this study are listed in Table S1. After washing three times in PBS containing 0.1% Tween-20 (P9416; Sigma-Aldrich), the PVDF membranes were incubated with horseradish peroxidase (HRP)–conjugated anti-rabbit IgG (7074S; Cell Signaling Technology) or anti-mouse IgG (7076S; Cell Signaling Technology) as secondary antibodies at room temperature for 1 h. Signals were developed using an Immobilon Western Chemiluminescent HRP Substrate (RPN2235; Cytiva) and examined using an Amasham imaging system (GE Healthcare Life Science). To analyze the intensity of bands, we used Fiji/ImageJ.

Table S1. List of antibodies in this study.

### Histochemical and immunofluorescence staining

Frozen 8-μm thin sections of the mouse soleus muscle or cultured cells were fixed 4% paraformaldehyde/PBS. For histochemical staining, sections were stained by Hematoxylin and Eosin Stain Kit (H-3502; Vector Laboratories) according to the manufacturerʼs instructions. For immunofluoresence staining, sections or cells washed with PBS containing 0.1% Triton X-100 (9002-93-1; Sigma-Aldrich) to permeabilize samples and incubated with 3% bovine serum albumin (BSA; 01281-26; Nacalai Tesque) at room temperature for 1 h to block nonspecific binding. Samples then incubated at 4°C overnight with the primary antibodies. The primary antibodies used in this study are listed in Table S1. After washing with PBS, the cells and sections were incubated with an Alexa Fluor 488 Goat Anti-Rabbit IgG H&L (ab150077; Abcam), Alexa Fluor 488 Goat Anti-Mouse IgG H&L (ab150113; Abcam), Alexa Fluor 594 Goat Anti-Rabbit IgG H&L (ab150080; Abcam), or an Alexa 594 Anti-Mouse IgG antibody (ab150116; Abcam) as a secondary antibody at room temperature for 1 h. the cells and sections were enclosed using ProLong Glass Antifade Mountant (P36965; Thermo Fisher Scientific) after staining the nuclei with 4′,6-diamidino-2-phenylindole (DAPI; D523; Dojindo) at room temperature for 5 min. The fluorescent images were obtained under a fluorescence microscope (Keyence). The total number of signal dots was counted separately in the nucleus and the cytoplasm using Fiji/ImageJ.

For immunofluorescence quantification of skeletal muscle sections, multiple fields were analyzed from each mouse. Values from fields within the same mouse were averaged before statistical analysis, and each mouse was treated as one biological replicate. Image acquisition and quantification were not performed under blinded conditions; however, identical acquisition settings and analysis criteria were applied across all experimental groups.

### Flow cytometry for the detection of proteins located on the extracellular surface of cells

Collected cells (1 × 10^6^ cells) incubated with PE-conjugated anti-mouse CD80 antibody (104707; BioLegend) or APC-conjugated anti-mouse CD206 antibody (141707; BioLegend) that diluted with DMEM containing 2% FBS at 4°C for 30 min. For quality control tested, we used PE-conjugated Mouse IgG2a, κ isotype control (400213; BioLegend) or APC-conjugated Rat IgG2a, κ isotype control (400511, BioLegend) under the same conditions. After washing with PBS, cells were stained with DAPI to exclude dead cells. All flow cytometric analyses were performed with a NovoCyte Flow Cytometer (Agilent). Obtained data were analyzed using the FlowJo software (Treestar).

### Enzyme-linked immuno-sorbent assay

Concentrations of the proinflammatory cytokines, TNF-α and IL-6 were determined from J774A.1 cells (1 × 10^6^ cells) culture supernatants using ELISA MAX Standard Set Mouse TNF-α (430901; BioLegend), ELISA MAX Standard Set Mouse IL-6 (431301; BioLegend) following the manufacturersʼ instructions. We measured absorbance at 450 nm using a Flexstaion 3 (Molecular Devices) within 30 min after adding TMB Substrate (2332458; ATTO).

### Quantitative RT-PCR (qRT-PCR)

Total RNA was prepared using the ISOSPIN Cell & Tissue RNA (314-08211; Nippon Gene) and was transcribed into cDNA using the ReverTra Ace qPCR RT Master Mix with gRNA remover (FSQ-301; Toyobo). The qRT-PCR was performed using PowerTrack SYBR Green Master Mix for qPCR (A46109; Thermo Fisher Scientific) with specific forward and reverse primers in a QuantStudio3 (Thermo Fisher Scientific). The relative gene expression was calculated with the ΔΔCt method (Livak & Schmittgen, 2001), using GAPDH for normalization. The primers used in this study are listed in Table S2.

Table S2. List of primers used for quantitative RT-PCR (qRT-PCR) in this study.

### Statistics

For all quantitative analyses, the biological replicate and statistical units are specified in the corresponding figure legends. For cell-based experiments, independent cultures or independent experiments were treated as biological replicates. For mouse tissue analyses, each mouse was treated as one biological replicate. When multiple cells, fields, myofibers, or myotubes were quantified within the same culture, dish, or mouse, these values were averaged before statistical analysis to avoid pseudo-replication. Statistical analysis was performed using R software (v4.5.1). We used *t* test to compare differences between two samples. We also used one-way ANOVA and Tukey–Kramer test for differences between the variances of the three groups. Values are presented as means ± SEM. Visualized and analyses were performed using the following packages: clusterProfiler (version 4.16.0), ggplot2 (version 4.0.0), ggpubr (version 0.6.1), org.Mm.eg.db (version 3.21.0), org.Hs.eg.db (version 3.21.0) from Bioconductor (Huber et al, 2015).

## Supplementary Material

Reviewer comments

## Data Availability

All data supporting the findings of this study are included in the article and supplementary materials. Pre-averaged numerical data underlying the image-based quantifications are provided in the accompanying Source Data files. The DYSF-3HA is available from Addgene (plasmid No. 29767). Public gene expression datasets (GSE56045 and GSE107566) are available from Gene Expression Omnibus (GEO). A previous version of this article was published as a preprint on bioRxiv: Tominago and Tominago (2025 *Preprint*).
