# Supplementary material for: Dysferlin promotes inflammatory macrophage responses and modulates Ca2+-dependent STAT1 signaling
Source: Life Sci Alliance. 2026 Oct 7;9(12):e202603669. doi: 10.26508/lsa.202603669 (PMC13646497; doi:10.26508/lsa.202603669)
Supplement: Supplementary file 1 [file LSA-2026-03669_review_history.pdf]

Dysferlin Promotes Inflammatory Macrophage Responses and Modulates Ca^{2+}-dependent STAT1 Signaling

Kana Tominaga and Naoomi Tominaga
DOI: https://doi.org/10.26508/lsa.202603669

Corresponding author(s): Kana Tominaga, Yamaguchi University

Review Timeline:

Submission Date:	2026-02-13
Editorial Decision:	2026-05-01
Revision Received:	2026-07-28
Editorial Decision:	2026-09-14
Revision Received:	2026-09-18
Editorial Decision:	2026-09-22
Revision Received:	2026-09-25
Accepted:	2026-09-28

Scientific Editor: Sarita Hebbar

Transaction Report:

(Note: With the exception of the correction of typographical or spelling errors that could be a source of ambiguity, letters and reports are not edited. The original formatting of letters and referee reports may not be reflected in this compilation.)

April 30, 2026

Re: Life Science Alliance manuscript #LSA-2026-03669-T

Dr. Kana Tominaga
Yamaguchi University Graduate School of Medicine
Department of Neuroscience
1-1-1 Minami-kogushi, Ube
Yamaguchi 755-8505
Japan

Dear Dr. Tominaga,

Thank you for submitting your manuscript entitled "Dysferlin programs inflammatory macrophage differentiation via Ca^{2+}-STAT1 signaling" to Life Science Alliance. We apologise for the delay in communicating our decision due to reviewer availability issues.

Your manuscript was evaluated by three reviewers whose comments are appended below. As you will note, all the reviewers have noted the potential value of this work but have also raised significant concerns. In line with their evaluation, we invite you to submit a revised manuscript addressing the reviewers' concerns.

-We agree with the reviewers that you must validate DYSF expression, characterisation of macrophage polarisation, and effects on muscle fibre formation in an appropriate physiological/cellular model. We bring your attention to the suggestions of Reviewer 1, points 1-2, Reviewer 3, major revisions and points 1, 5 and Reviewer 2, in-vivo relevance) and leave the choice of experimental system upto you.

-We concur with Reviewer 3, point 3, that you must complement your findings in the overexpression system, with the knockdown of DYSF for major phenotypes. Further in line with the comments from Reviewer 2 (STAT1 signalling) and Reviewer 3, point 2, you must either demonstrate that calcium signalling is required for STAT1 activation downstream of DYSF or temper the mechanistic claims made in this work.

-Reviewer 1 (points 3, 5) raises valid concerns on analyses of myofibers and co-lableling with pan macrophage-makers which we agree must be resolved in a revised version

We appreciate the experimental suggestions of Reviewer 3 towards a comprehensive characterisation of macrophage phenotypes and gene expression changes by DYSF (point 3). However, we think that adding such data is not necessary but you must highlight the limitation that your study does not capture the heterogeneity of macrophages.

When submitting the revision, please include a letter addressing the reviewers' comments point by point. While a rebuttal must respond to all points in some form, additional experiments to resolve these points, other than indicated above, will not be required.

I would be happy to discuss the revision in more detail via email or phone/videoconferencing. Please let me know which option you prefer, if any.

To upload the revised version of your manuscript, please log in to your account: https://lsa.msubmit.net/cgi-bin/main.plex

You will be guided to complete the submission of your revised manuscript and to fill in all necessary information. Please get in touch in case you do not know or remember your login name.

The typical timeframe for revisions is three months. Please note that papers are generally considered through only one revision cycle, so strong support from the referees on the revised version is needed for acceptance.

While you are revising your manuscript, please also attend to the below editorial points to help expedite the publication of your manuscript. Please direct any editorial questions to the journal office.

Thank you for this interesting contribution to Life Science Alliance. We hope that the comments below will prove constructive as your work progresses, and we are looking forward to receiving your revised manuscript.

Sincerely,

Sarita Hebbar, PhD

Scientific Editor
Life Science Alliance
http://www.lsajournal.org

A. THESE ITEMS ARE REQUIRED FOR REVISIONS

-- A letter addressing the reviewers' comments point by point.

-- An editable version of the final text (.DOC or .DOCX) is needed for copyediting (no PDFs).

-- High-resolution figure, supplementary figure and video files uploaded as individual files: See our detailed guidelines for preparing your production-ready images, https://www.life-science-alliance.org/authors

-- Summary blurb (enter in submission system): A short text summarizing in a single sentence the study (max. 200 characters including spaces). This text is used in conjunction with the titles of papers, hence should be informative and complementary to the title and running title. It should describe the context and significance of the findings for a general readership; it should be written in the present tense and refer to the work in the third person. Author names should not be mentioned.

-- By submitting a revision, you attest that you are aware of our payment policies found here: https://www.life-science-alliance.org/copyright-license-fee

B. MANUSCRIPT ORGANIZATION AND FORMATTING:

Full guidelines are available on our Instructions for Authors page, https://www.life-science-alliance.org/authors

We encourage our authors to provide original source data, particularly uncropped/-processed electrophoretic blots and spreadsheets for the main figures of the manuscript. If you would like to add source data, we would welcome one PDF/Excel-file per figure for this information. These files will be linked as supplementary "Source Data" files.

IMPORTANT: It is Life Science Alliance policy that if requested, original data images must be made available. Failure to provide original images upon request will result in unavoidable delays in publication. Please ensure that you have access to all original microscopy and blot data images before submitting your revision.

Reviewer #1 (Comments to the Authors (Required)):

In this manuscript, the authors aim at studying the role of Dysferlin (DYSF) expressed by muscle macrophages in the control of their inflammatory status, and how its overexpression can be associated with altered macrophage function and muscle homeostasis during aging. To do so, the authors mainly use gain and loss of functions experiments through Dysf overexpression or knock-down in murine macrophage cell line. Globally, they show that DYSF induces a pro-inflammatory macrophage phenotype in vitro, through the regulation of intracellular calcium and the activation of STAT1. Finally, they observe a correlation between
Macrophages are essential for muscle repair after an injury and the alteration of macrophage inflammatory profile and function is associated with various pathological conditions such as muscular dystrophies, including dysferlinopathies. Therefore, the identification of new molecular effectors regulating macrophage function in muscle is of high importance as it could allow the identification of potential new therapeutic targets to modulate inflammation and improve muscle homeostasis.
Although the direct role of macrophage DYSF in regulating muscle macrophage function would be of interest, there are some conceptual and methodological concerns that would need to be addressed to fully support the authors conclusions.

Major points:

1) The authors investigate the expression of DYSF in the monocyte to macrophage differentiation in vitro, but they do not compare its level of expression between pro- and anti-inflammatory macrophages which would have a better relevance for the potential role of DYSF in muscle macrophages in vivo. For example, measuring Dysf mRNA/protein in polarised macrophage cell line in vitro and confirming its expression on pro- and anti-inflammatory macrophages sorted from muscle after an injury (here the authors may look at single cell RNAseq data available on muscle during regeneration and aging such as PMID:32248064 ; PMID:39541212)

2) All the in vitro analyses regarding macrophage inflammatory status are performed on a cell line (mouse J774A.1) polarised towards a pro-inflammatory or anti-inflammatory profile using IFNg or IL4, respectively. This choice is questionable as these cytokines do not recapitulate the macrophage status observed in vivo for example during muscle regeneration

(PMID:27183604). Therefore, it is important to confirm in vivo the results observed by the authors. For example, is the phenotype observed in vitro through siRNA treatment recapitulated in mice harboring Dysferlin inactivation in macrophages using a Dysffl/fl with CD64-CRE, Cx3cr1-CRE or even LysM-CRE? In this context, it is the Dysfl inactivation in macrophages prevents the phenotype observed in old animals?

3) The authors observe an increase in myofiber cross-sectional area in old males as compared to young males. This is surprising, as aging is more associated with sarcopenia and a reduction of myofiber size (Figure EV6B). However, there is no information about the number of muscles analysed. It looks like the authors quantified 50 myofibers per conditions and consider each myofiber as a biological replicate. The authors should analyse all the myofibers within a whole muscle section (as 50 myofibers are not representative of the muscle), average the CSA for one muscle and consider this as a single biological replicate to make sure that the phenotype they observe is robust. Moreover, it would be of interest to analyse other muscles such as the tibialis anterior, the gastrocnemius or the diaphragm to evaluate the specificity of the phenotype.

4) The authors observe an increase of macrophages expressing DYSF in old animals (both males and females) and an increased myofiber size (only in males), suggesting a decorrelation between high DYSF expression in macrophages and its impact on muscle homeostasis in aging, weakening the authors conclusion. The authors should discuss this point.

5) In Figure EV6C-F, the authors use immunolabeling on muscle sections to discriminate pro-inflammatory (with CD80) and anti-inflammatory macrophages (with CD206). These markers are not specific for macrophages. For example, CD206 can be expressed by myogenic cells and it is important for their fusion (PMID:16864654). Thus, a difference in CD206 levels could be indicative of an impact on myogenic cells and not on macrophages. Therefore, the authors should perform a co-labeling with a pan-macrophage marker such as F4/80 or CD64 and count the number or proportion of macrophages expressing either the pro- or the anti-inflammatory marker.

Minor points:

6) In Figure EV2C, it is not clear how many biological replicates are analyzed. It seems that for the quantification, each cell is considered as a biological replicate, which is not correct. The authors should average the result for all the cells from the same culture and consider this as a single biological replicate

7) In Figure 5, as an in vivo validation, the authors first mesure Dysf mRNA and protein levels in bone marrow-derived monocytes from young or old mice. Then, the authors measure DYSF levels in macrophages on muscle sections (Figure 5D-E). However, it is not clear what the authors quantify as they mention signal intensity in the Figure legend but show a percentage in the graph.

8) In Figure EV6C-FMoreover, it is not clear what the authors consider as a biological replicate as they mention n=3 in the legend but show more individual data points in the graphs. The results obtained from different fields within the same muscle should be averaged and considered as only one biological replicate

9) There are variations between blots for DYSF protein. Sometimes there is only one band (Fig EV1A), sometimes there are 2 bands (FigEV2A). The authors should discuss these differences and explain which bands are considered for their quantification.

10) The authors should not use the M1/M2 classification when they refer to the macrophage inflammatory state in muscle in vivo as it corresponds to a classification established in vitro which does not recapitulate the subpopulations status in vivo.

Reviewer #2 (Comments to the Authors (Required)):

Reviewer Comments:

This manuscript presents compelling evidence for a novel role of DYSF in regulating macrophage polarization, Ca^{2+} signaling, and myoblast fusion. The findings are mechanistically interesting and physiologically relevant, especially in the context of aging. However, key mechanistic links (Ca^{2+} → STAT1), validation in primary cells, in vivo functional relevance, and specificity of STAT1 activation need further investigation. Addressing these points will significantly strengthen the manuscript.

The study shows that DYSF overexpression increases Ca^{2+} influx and promotes STAT1 phosphorylation and nuclear translocation.

Does chelation of intracellular Ca^{2+} or inhibition of Orai channels prevent STAT1 activation in DYSF-overexpressing cells?

STAT1 inhibition (e.g., fludarabine or siSTAT1) → cytokine output?

The analysis of STAT1 signaling is limited and raises concerns:

Canonical STAT1 activation is typically defined by phosphorylation at Tyr701, yet the study emphasizes Ser727 phosphorylation and unphosphorylated STAT1.

Functional evidence of STAT1 transcriptional activity is not provided (e.g., reporter assays or broader target gene analysis).

Can these findings be validated in primary human monocyte-derived macrophages to strengthen translational relevance?

In vivo relevance

The manuscript shows DYSF accumulation in aged mouse macrophages and skeletal muscle. Can the authors demonstrate functional consequences in vivo? Does DYSF drive aging phenotype?

Does DYSF knockdown in aged mice alter macrophage polarization or muscle inflammation? Does overexpression in vivo impact muscle regeneration after injury?

DYSF and M2 polarization

The data indicate DYSF has a weak effect on M2 polarization. Has the effect of DYSF knockdown on M2 polarization under IL-4 stimulation been fully explored? Could DYSF actively suppress M2 differentiation?

Impact on myogenesis

Co-culture experiments suggest DYSF-overexpressing macrophages reduce myoblast fusion via TNF-α and IL-6. Could the authors use neutralizing antibodies or cytokine inhibitors to definitively show that these cytokines mediate the effect?

Most experiments use single overexpression or knockdown conditions. Would a dose-dependent study of DYSF expression strengthen the link between DYSF levels, macrophage polarization, and Ca^{2+} influx?

STAT1 specificity

DYSF may affect multiple signaling pathways. Did the authors test whether other STAT family members (STAT3/STAT6) or NF-κB signaling are influenced by DYSF? This would clarify specificity to STAT1.

Minor Comments

Morphological analysis (cell area, circularity) should not be overinterpreted as evidence of differentiation.

Statistical details (n values, replicates) should be consistently reported across all figures.

Some claims in the Discussion (e.g., direct regulation of STAT1 by Ca^{2+}) are overstated relative to the data.

Clarify whether sex-specific differences were observed in aging experiments.

Improve clarity in figure legends, particularly regarding normalization and quantification methods.

The manuscript presents a comprehensive and novel study linking DYSF to macrophage polarization and myogenesis. The experiments are well designed and mechanistic insights are strong. However, to fully establish causality and translational relevance, additional experiments in primary human macrophages, in vivo functional validation, and more rigorous testing of STAT1 specificity would strengthen the conclusions.

Reviewer #3 (Comments to the Authors (Required)):

The manuscript by Tominaga et al. explores the role of dysferlin (DYSF) in macrophage differentiation and function, proposing that DYSF promotes inflammatory macrophage polarization through a Ca^{2+}-dependent activation of STAT1 signaling. The authors further suggest that DYSF-enriched macrophages impair myogenic differentiation and that DYSF expression increases with aging, potentially linking this pathway to age-associated muscle dysfunction. The topic is interesting and potentially impactful, particularly given the limited understanding of dysferlin function outside muscle membrane repair. However, although the study presents intriguing observations, several conceptual and experimental limitations substantially weaken the strength of the conclusions.

Major Revisions

The central claims of the manuscript, namely that DYSF drives inflammatory macrophage polarization and negatively affects

myogenesis, are supported almost entirely by in vitro systems, including macrophage cell lines and co-culture assays with C2C12 myoblasts. While the authors include in vivo data showing increased DYSF expression in aged macrophages, these observations are purely descriptive and do not establish causality. There is no demonstration that DYSF functionally contributes to macrophage polarization or muscle regeneration in vivo. Given the physiological relevance emphasized throughout the manuscript, this represents a critical gap that must be addressed.

1. The authors should incorporate loss-of-function approaches in vivo to substantiate the proposed role of DYSF in tissue contexts. Macrophage-specific Dysf deletion using conditional knockout strategies would allow direct testing of whether DYSF is required for inflammatory macrophage polarization during muscle injury or aging. Alternatively, bone marrow transplantation experiments using Dysf-deficient donor cells could assess the contribution of macrophage DYSF to muscle regeneration outcomes. Muscle injury models, such as cardiotoxin or freeze injury, would provide physiologically relevant contexts in which to evaluate the impact of macrophage DYSF on tissue repair, inflammation resolution, and myogenic differentiation. Without such in vivo functional validation, the manuscript remains largely descriptive and the mechanistic claims lack the necessary support for publication.

2. While the authors propose a DYSF-Ca^{2+}-STAT1 signaling axis, the evidence remains largely correlative rather than mechanistic. Calcium influx is demonstrated using pharmacological tools and ionophores, but there is no direct demonstration that calcium signaling is required for STAT1 activation downstream of DYSF. Similarly, the necessity of STAT1 for the observed inflammatory phenotype is not tested experimentally. Without such evidence, the proposed signaling cascade remains speculative.

To establish this mechanism rigorously, the authors should perform experiments involving calcium chelation to determine whether blocking intracellular calcium prevents DYSF-induced STAT1 activation and inflammatory marker expression. Additionally, STAT1 inhibition or knockdown experiments should be conducted to test whether STAT1 is required for the DYSF-mediated inflammatory phenotype. Rescue experiments, in which STAT1 activity is restored in the presence of calcium chelation, would further strengthen the proposed linear pathway.

3. Many of the key findings, including changes in macrophage morphology, cytokine production, migration, and STAT1 activation, are derived from DYSF overexpression in J774A.1 cells. Although knockdown experiments are included, they are less comprehensive and not consistently integrated across all assays. Overexpression can introduce artifacts, particularly for a protein involved in membrane dynamics and calcium handling, and may not accurately reflect physiological regulation of DYSF. The authors should place greater emphasis on loss-of-function approaches throughout the study. Knockdown or knockout of DYSF should be performed systematically across all major phenotypic assays, including polarization marker expression, cytokine secretion, migration, and co-culture experiments. Furthermore, validation in primary macrophages, such as bone marrow-derived macrophages (BMDMs) from wild-type and Dysf-deficient mice, would significantly strengthen the conclusions by demonstrating that the observed phenotypes occur under physiologically relevant conditions. The current reliance on overexpression in a cell line limits the interpretability and generalizability of the findings.

4. The manuscript relies heavily on the M1/M2 dichotomy, supported by a limited set of markers such as iNOS, STAT1, CD80, Arg1, and CD206. This simplified classification does not capture the complexity and heterogeneity of macrophage states, especially in the context of aging and muscle tissue, where macrophages exhibit diverse and context-dependent phenotypes that extend beyond classical M1/M2 categories. A more comprehensive characterization of macrophage phenotypes is needed. The authors should consider employing transcriptomic approaches, such as RNA sequencing, to provide an unbiased assessment of the gene expression changes induced by DYSF modulation.

5. The co-culture experiments between macrophages and myoblasts represent an important attempt to connect macrophage programming to functional consequences in muscle cells. However, these experiments are limited to a Transwell system and primarily attribute the observed effects to TNF-α and IL-6. While cytokine neutralization partially rescues myogenic markers, the analysis remains narrow and does not explore additional signaling pathways, secreted factors, or cell-contact-dependent mechanisms that may contribute to the phenotype. The authors should extend these experiments to include broader secretome analysis, such as multiplex cytokine arrays or proteomic profiling of conditioned media, to identify additional factors that may mediate the effects of DYSF-expressing macrophages on myogenesis. Validation in primary myoblasts or satellite cells, rather than relying solely on the C2C12 cell line, would enhance the physiological relevance of these findings. Additionally, direct co-culture experiments, which allow cell-cell contact, could reveal contact-dependent mechanisms that are not captured in the Transwell system. These extensions would provide a more complete understanding of how DYSF-expressing macrophages influence muscle cell behavior.

Minor Revisions

1. The manuscript would benefit from thorough language editing to improve clarity and readability. There are multiple instances of awkward phrasing, grammatical inconsistencies, and unclear sentence structures that detract from the overall presentation. Professional editing or careful revision by a native English speaker would enhance the manuscript's accessibility and professionalism.

2. Statistical reporting is not always sufficiently detailed throughout the manuscript. The definitions of biological versus technical replicates are unclear in several experiments, and exact p-values are not consistently reported. The authors should provide complete statistical information, including sample sizes, the number of independent experiments, definitions of error bars, and exact p-values where appropriate. Additionally, some experimental descriptions lack sufficient detail for reproducibility. For example, transfection efficiency is not reported, cytokine concentrations used in some assays are not specified, and the timing of certain treatments is ambiguous. These details should be clarified in the methods section or figure legends.

3. The calcium imaging experiments rely heavily on non-physiological stimuli such as ionomycin and other ionophores to induce calcium influx. While these tools are useful for demonstrating calcium handling capacity, they may not accurately reflect the physiological regulation of calcium signaling downstream of DYSF. The authors should consider including experiments with

more physiologically relevant stimuli, such as receptor-mediated calcium mobilization or stimuli that mimic inflammatory conditions, to strengthen the biological relevance of the calcium signaling data.
4. The manuscript places considerable emphasis on STAT1 phosphorylation at Ser727, but the rationale for focusing on this site rather than the canonical Tyr701 phosphorylation is not clearly explained. While Ser727 phosphorylation can modulate STAT1 activity, Tyr701 phosphorylation is typically considered the primary activating event. The authors should provide clearer justification for their focus on Ser727, discuss the functional significance of this modification in the context of their findings, and ideally include data on Tyr701 phosphorylation to provide a more complete picture of STAT1 activation.
5. Several figures could be improved in terms of clarity, labeling, and organization. Some panels lack scale bars or have inconsistent labeling conventions. Quantifications are not always clearly linked to their corresponding representative images. The authors should ensure that all figures are clearly labeled, that statistical comparisons are unambiguous, and that figure legends provide sufficient detail to interpret the data without referring extensively to the main text. High-resolution images and consistent formatting across figures would also enhance the overall presentation.

Recommendation
In summary, this study presents a potentially novel role for dysferlin in macrophage biology and suggests an interesting link between calcium signaling, STAT1 activation, and inflammatory polarization. However, the current version of the manuscript relies heavily on in vitro systems, lacks definitive mechanistic evidence, and does not provide sufficient in vivo validation to support its central claims. The major revisions outlined above are essential to establish causality, clarify mechanisms, and demonstrate physiological relevance. Substantial additional work would be required to elevate the study to the level expected for publication. I therefore recommend major revision.

Point-by-point to the comments from the Reviewers

Below, we summarize how we addressed each comment. Revisions made in response to the reviewers' comments are highlighted in blue in the revised manuscript. In addition, minor corrections from the previous version, including typographical and spelling errors, are highlighted in red. To address the reviewers' concerns regarding potential overstatement, we changed the title from "Dysferlin Programs Inflammatory Macrophage Differentiation via Ca^{2+}–STAT1 Signaling" to "**Dysferlin Promotes Inflammatory Macrophage-associated Activation via Ca^{2+}-dependent STAT1 Signaling.**" The revised title avoids implying definitive macrophage differentiation or canonical STAT1 activation and more accurately reflects the scope of our data.

Reviewer #1:

In this manuscript, the authors aim at studying the role of Dysferlin (DYSF) expressed by muscle macrophages in the control of their inflammatory status, and how its overexpression can be associated with altered macrophage function and muscle homeostasis during aging. To do so, the authors mainly use gain and loss of functions experiments through Dysf overexpression or knock-down in murine macrophage cell line. Globally, they show that DYSF induces a pro-inflammatory macrophage phenotype in vitro, through the regulation of intracellular calcium and the activation of STAT1. Finally, they observe a correlation between

Macrophages are essential for muscle repair after an injury and the alteration of macrophage inflammatory profile and function is associated with various pathological conditions such as muscular dystrophies, including dysferlinopathies. Therefore, the identification of new molecular effectors regulating macrophage function in muscle is of high importance as it could allow the identification of potential new therapeutic targets to modulate inflammation and improve muscle homeostasis.

Although the direct role of macrophage DYSF in regulating muscle macrophage function would be of interest, there are some conceptual and methodological concerns that would need to be addressed to fully support the authors conclusions.

Response to Reviewer #1:

We thank the reviewer for the careful assessment of our manuscript and for recognizing the importance of identifying molecular regulators of macrophage function in skeletal muscle. We agree that the direct contribution of macrophage DYSF to macrophage inflammatory status and muscle homeostasis requires careful interpretation.

In response to the reviewer's conceptual and methodological concerns, we have substantially revised the manuscript and added new experiments. First, we revised the terminology throughout the manuscript to avoid relying on a strict M1/M2 dichotomy and now describe the phenotype as "inflammatory macrophage-like activation" or "inflammatory macrophage-associated features." Second, we strengthened the loss-of-function evidence by adding Dysf knockdown experiments, showing that Dysf depletion attenuates inflammatory gene expression, cytokine production, Ca^{2+} responses, STAT1 phosphorylation, and macrophage-mediated suppression of myogenic differentiation. Third, we added Ca^{2+} inhibition and STAT1 knockdown experiments demonstrating that DYSF-associated STAT1 Ser727 phosphorylation, STAT1 nuclear localization, and STAT1-responsive gene expression depend, at least in part, on Ca^{2+} influx and STAT1 signaling. Fourth, we added F4/80/Dysferlin co-immunostaining to show the accumulation of DYSF-expressing macrophage-lineage cells in aged skeletal muscle.

We have also revised the Discussion to clarify that our aged tissue analyses support *in vivo* relevance but do not establish direct in vivo causality. We now explicitly state that macrophage-specific Dysf loss-of-function models, bone marrow transplantation, and injury or aging models will be required in future

studies. We believe that these revisions address the reviewer's concerns and provide a more balanced interpretation of the role of DYSF in macrophage inflammatory activation and myogenic suppression.

Major points:

1) The authors investigate the expression of DYSF in the monocyte to macrophage differentiation in vitro, but they do not compare its level of expression between pro- and anti-inflammatory macrophages which would have a better relevance for the potential role of DYSF in muscle macrophages in vivo. For example, measuring Dysf mRNA/protein in polarised macrophage cell line in vitro and confirming its expression on pro- and anti-inflammatory macrophages sorted from muscle after an injury (here the authors may look at single cell RNAseq data available on muscle during regeneration and aging such as PMID:32248064 ; PMID:39541212)

Response:

We thank the reviewer for this important suggestion. We agree that comparing DYSF expression between inflammatory and alternative macrophage-associated states is important for understanding the potential relevance of DYSF in muscle macrophages *in vivo*.

In response to this comment, we performed additional experiments using cytokine-stimulated macrophage-like cells. Specifically, we examined endogenous DYSF expression in PMA-differentiated U937 macrophage-like cells treated with IFN-γ or IL-4. IFN-γ stimulation increased *DYSF* mRNA expression and DYSF protein levels, whereas IL-4 showed a weaker effect. These data have been added to Fig. 1E–G and the corresponding Results section. We also assessed inflammatory and alternative macrophage-associated markers in J774A.1 cells under IFN-γ- or IL-4-stimulated conditions. DYSF overexpression enhanced IFN-γ-associated inflammatory marker expression, whereas Dysf knockdown reduced IFN-γ-induced inflammatory marker expression and enhanced IL-4-induced alternative macrophage-associated marker expression. These data are shown in Fig. 2 and Fig. EV2.

To strengthen the *in vivo* relevance of DYSF expression in muscle macrophage-lineage cells, we also revised the tissue analysis. We added co-immunostaining for F4/80 and Dysferlin in aged skeletal muscle. This analysis showed that F4/80$^+$Dysferlin$^+$ macrophage-lineage cells were increased in aged skeletal muscle. These data have been added to Fig. 5D-E. We also moved the previous CD11b/Dysferlin staining to the supplementary figures as supporting data (Figure EV6). In addition, we assessed CD80- and CD206-positive cells in aged skeletal muscle to characterize inflammatory and alternative macrophage-associated features in the tissue context.

We agree that analysis of macrophages sorted from injured muscle or reanalysis of public single-cell RNA-seq datasets during muscle regeneration and aging would provide additional information regarding DYSF expression across macrophage subpopulations *in vivo*. However, due to the limited revision timeframe and the absence of newly generated sorted macrophage samples from injury models, we were not able to complete a sufficiently rigorous sorted-cell or single-cell RNA-seq analysis for the current revision. We have therefore revised the manuscript to avoid overinterpreting our tissue data as direct evidence of DYSF expression in defined pro- or anti-inflammatory macrophage subsets in vivo. We have also added this point to the Discussion as an important future direction.

Revised text has been added to the Results and Discussion sections as follows:

Line # 110-117

To further address whether DYSF expression is associated with macrophage activation stimuli, we examined endogenous DYSF expression in PMA-differentiated U937 macrophage-like cells treated with IFN-γ or IL-4. IFN-γ stimulation increased DYSF mRNA expression, whereas IL-4 did not markedly increase DYSF expression (Fig. 1E). Immunoblot analysis further showed increased DYSF protein levels

after IFN-γ stimulation, and STAT1 Tyr701 phosphorylation was confirmed as a marker of IFN-γ responsiveness (Fig. 1F, G). These findings indicate that DYSF is increased during monocyte-to-macrophage differentiation and is further enhanced by inflammatory stimulation in human macrophage-like cells.

Line # 284-292

To more directly identify DYSF-expressing macrophage-lineage cells in skeletal muscle, we performed co-immunostaining for F4/80 and Dysferlin. F4/80-positive cells were rarely detected in young skeletal muscle but were increased in aged skeletal muscle. Importantly, $F4/80^{+}Dysferlin^{+}$ cells were more frequently observed in aged mice than in young mice (Fig. 5D, E). No clear difference was observed between males and females within either age group. Consistent with this finding, co-immunostaining for CD11b and Dysferlin also showed increased $CD11b^{+}Dysferlin^{+}$ cells in aged skeletal muscle (Fig. EV7A, B). These findings indicate that DYSF-expressing macrophage-lineage cells accumulate in aged skeletal muscle and support the association between aging, macrophage-lineage cell accumulation, and increased DYSF expression in skeletal muscle tissue.

Line # 429-432

Moreover, macrophages sorted from injured or aged skeletal muscle, as well as single-cell transcriptomic analyses, will be needed to define DYSF expression across macrophage subpopulations during muscle regeneration and aging.

2) All the in vitro analyses regarding macrophage inflammatory status are performed on a cell line (mouse J774A.1) polarised towards a pro-inflammatory or anti-inflammatory profile using IFNg or IL4, respectively. This choice is questionable as these cytokines do not recapitulate the macrophage status observed in vivo for example during muscle regeneration (PMID:27183604). Therefore, it is important to confirm in vivo the results observed by the authors. For example, is the phenotype observed in vitro through siRNA treatment recapitulated in mice harboring Dysferlin inactivation in macrophages using a Dysffl/fl with CD64-CRE, Cx3cr1-CRE or even LysM-CRE? In this context, it is the Dysfl inactivation in macrophages prevents the phenotype observed in old animals?

Response:

We thank the reviewer for this important and constructive suggestion. We agree that macrophage-specific Dysf knockout models would provide definitive evidence for the *in vivo* role of DYSF.

Although such experiments were not feasible within the timeframe of the present revision, we have strengthened the physiological relevance of our study by further analyzing aged mouse macrophages and discussing the limitations of the current *in vitro* polarization model. We consider *in vivo* experiment an important direction for future studies.

Therefore, we have revised the Results and Discussion to avoid concluding that macrophage DYSF directly causes the aged-muscle phenotype *in vivo*. We now state that macrophage-specific Dysf loss-of-function models, bone marrow transplantation, and muscle injury or aging models will be required in future studies to determine whether Dysf inactivation in macrophages prevents age-associated inflammatory and myogenic defects.

The following sentences have been added or revised in the manuscript:

Line # 356-361

In this study, IFN-γ and IL-4 were used as defined in vitro stimuli to examine DYSF-dependent inflammatory and alternative macrophage-associated responses, respectively. These conditions do not fully recapitulate the dynamic macrophage states observed during muscle regeneration in vivo. Therefore, we refer to DYSF-associated macrophage responses as inflammatory macrophage-like activation or

inflammatory macrophage-associated features, rather than defining them strictly as fixed M1 or M2 macrophage states.

Line # 422-427

However, several limitations should be noted. First, although our aged-mouse tissue analyses demonstrate the accumulation of DYSF-expressing F4/80-positive macrophage-lineage cells in skeletal muscle, they do not establish in vivo causality. Macrophage-specific Dysf loss-of-function models, bone marrow transplantation, and muscle injury or aging models will be required to determine whether macrophage DYSF directly contributes to impaired muscle regeneration *in vivo.*

3) The authors observe an increase in myofiber cross-sectional area in old males as compared to young males. This is surprising, as aging is more associated with sarcopenia and a reduction of myofiber size (Figure EV6B). However, there is no information about the number of muscles analysed. It looks like the authors quantified 50 myofibers per conditions and consider each myofiber as a biological replicate. The authors should analyse all the myofibers within a whole muscle section (as 50 myofibers are not representative of the muscle), average the CSA for one muscle and consider this as a single biological replicate to make sure that the phenotype they observe is robust. Moreover, it would be of interest to analyse other muscles such as the tibialis anterior, the gastrocnemius or the diaphragm to evaluate the specificity of the phenotype.

Response:

We thank the reviewer for this important comment. In response, we reanalyzed the CSA data using the mouse as the biological replicate. Multiple fields were analyzed from each muscle section, and values from all analyzed myofibers within the same mouse were averaged before statistical analysis. We have revised the Methods and Figure EV6 legend accordingly.

After this reanalysis, the apparent increase in CSA in aged male mice remained statistically significant. However, we agree that this finding should be interpreted cautiously, because aging is generally associated with sarcopenia and reduced myofiber size. We therefore revised the Results to avoid interpreting this observation as evidence of age-associated myofiber hypertrophy. Instead, we now describe it as an apparent CSA increase observed in the context of aged skeletal muscle remodeling, including increased interstitial spaces and connective tissue-like changes.

We also agree that whole-section quantification and analysis of additional muscles, such as tibialis anterior, gastrocnemius, and diaphragm, would be valuable. These analyses were not possible within the current revision because additional aged muscle samples were not available. We have added this point to the Discussion as a limitation and future direction.

The revised Results section now states:

Line # 271-277

Next, histological analyses were performed to assess age-associated changes in skeletal muscle tissue (Fig. EV6A). Myofiber cross-sectional area (CSA) was increased in aged skeletal muscle in both male and female mice, with no clear sex-specific difference (Fig. EV6B). Although an apparent increase in CSA was observed with aging, this finding should be interpreted cautiously because aging skeletal muscle also showed increased interstitial spaces, intramuscular fat, and connective tissue-like changes. These histological observations indicate that aged skeletal muscle exhibits structural alterations consistent with age-associated tissue remodeling.

The revised Figure EV6 legend now states:

Figure EV6

B Myofiber CSA was quantified from multiple fields in each muscle section. Values from all analyzed myofibers within the same mouse were averaged before statistical analysis, and each mouse was treated as one biological replicate. Each dot represents one mouse.

We have also added the following statement to the Discussion:

Line # 427-429

In addition, although CSA was analyzed using the mouse as the biological replicate, complete whole-section quantification was not performed. Analyses of whole muscle sections and additional muscles will be needed to confirm the robustness and muscle-type specificity of the apparent CSA changes.

4) The authors observe an increase of macrophages expressing DYSF in old animals (both males and females) and an increased myofiber size (only in males), suggesting a decorrelation between high DYSF expression in macrophages and its impact on muscle homeostasis in aging, weakening the authors conclusion. The authors should discuss this point.

Response:

We thank the reviewer for raising this important point. We agree that, in the originally submitted analysis, the apparent increase in myofiber CSA only in aged male mice appeared to be dissociated from the increase in DYSF-expressing macrophage-lineage cells, which was observed in both males and females.

In response to the reviewer's comments, we reanalyzed the CSA data using the mouse, rather than individual myofibers, as the biological replicate. Multiple fields were analyzed from each muscle section, and values from all analyzed myofibers within the same mouse were averaged before statistical analysis. After this reanalysis, the apparent increase in CSA was observed in both aged male and aged female mice. We have revised Fig. EV6B, the Figure EV6 legend, the Methods, and the Results section accordingly.

Nevertheless, we agree that this observation should be interpreted cautiously. Although both the accumulation of DYSF-expressing F4/80-positive macrophage-lineage cells and the apparent increase in CSA were observed in aged mice of both female and male, these data do not establish a direct causal relationship between macrophage DYSF expression and myofiber size. CSA alone does not fully represent muscle homeostasis, and aged skeletal muscle showed structural alterations, including increased interstitial spaces and connective tissue-like changes, which may influence apparent CSA measurements.

Therefore, we have revised the manuscript to clarify that the aged-muscle data support an association between aging, accumulation of DYSF-expressing macrophage-lineage cells, and skeletal muscle remodeling, but do not establish that macrophage DYSF directly controls myofiber size in vivo. The functional link between macrophage DYSF and myogenic suppression is instead supported by our macrophage–myoblast co-culture experiments, in which DYSF-overexpressing macrophages suppressed myogenic differentiation and Dysf knockdown attenuated this inhibitory effect.

The revised Discussion now states:

Line # 343-348

DYSF-expressing F4/80-positive macrophage-lineage cells accumulated in aged skeletal muscle in both males and females. However, this finding does not establish a direct causal relationship between macrophage DYSF expression and myofiber size. CSA alone does not fully reflect muscle homeostasis, and age-associated interstitial remodeling, connective tissue accumulation, fat infiltration, fiber type composition, and systemic inflammatory cues may influence the apparent CSA phenotype.

We have also added the following limitation:

Line # 422-427

However, several limitations should be noted. First, although our aged-mouse tissue analyses demonstrate the accumulation of DYSF-expressing F4/80-positive macrophage-lineage cells in skeletal muscle, they do not establish in vivo causality. Macrophage-specific Dysf loss-of-function models, bone marrow transplantation, and muscle injury or aging models will be required to determine whether macrophage DYSF directly contributes to impaired muscle regeneration *in vivo.*

5) In Figure EV6C-F, the authors use immunolabeling on muscle sections to discriminate pro-inflammatory (with CD80) and anti-inflammatory macrophages (with CD206). These markers are not specific for macrophages. For example, CD206 can be expressed by myogenic cells and it is important for their fusion (PMID:16864654). Thus, a difference in CD206 levels could be indicative of an impact on myogenic cells and not on macrophages. Therefore, the authors should perform a co-labeling with a pan-macrophage marker such as F4/80 or CD64 and count the number or proportion of macrophages expressing either the pro- or the anti-inflammatory marker.

Response:

We thank the reviewer for this important comment. We agree that CD80 and CD206 are not specific markers for macrophages in skeletal muscle sections and that CD206, in particular, can also be expressed by myogenic cells. Therefore, CD80 or CD206 single immunolabeling should not be interpreted as definitive evidence for pro-inflammatory or anti-inflammatory macrophage populations.

In response to this concern, we revised the text, figure legend, and interpretation of Fig. EV6C–F. We no longer describe CD80-positive and CD206-positive signals as "pro-inflammatory macrophages" or "anti-inflammatory macrophages." Instead, we now refer to them as "CD80-positive cells," "CD206-positive cells," or "inflammatory/alternative macrophage-associated marker immunoreactivity." We also removed statements implying that CD80/CD206 single labeling directly defines macrophage subsets in aged skeletal muscle.

To more directly identify macrophage-lineage cells expressing DYSF in skeletal muscle, we added co-immunostaining for F4/80 and Dysferlin in Fig. 5D and quantified F4/80$^+$Dysferlin$^+$ cells in Fig. 5E. This analysis showed that F4/80$^+$Dysferlin$^+$ macrophage-lineage cells were increased in aged skeletal muscle. We also moved the previous CD11b/Dysferlin staining to Supplementary Fig. EV7 as supporting evidence. These revisions strengthen the identification of DYSF-expressing macrophage-lineage cells in aged muscle.

We agree that co-labeling of F4/80 or CD64 with CD80 and CD206, followed by quantification of the proportion of macrophages expressing these markers, would provide a more precise assessment of macrophage inflammatory states in vivo. Because such additional multi-marker tissue analysis was not completed within the current revision timeframe, we have revised the manuscript to avoid overinterpreting CD80/CD206 single staining.

The revised Results section now states:

Line # 278-283

We then assessed inflammatory and alternative macrophage-associated markers in skeletal muscle sections by immunofluorescence. Immunostaining for CD80 revealed increased accumulation of CD80-positive cells in aged skeletal muscle (Fig. EV6C, D). Conversely, there were fewer differences in CD206-positive cells between young and aged skeletal muscle (Fig. EV6E, F). Because CD80 and CD206 are not specific

to macrophages in skeletal muscle sections, these data were interpreted as tissue-level marker immunoreactivity rather than definitive identification of macrophage subsets.

Minor points:

6) In Figure EV2C, it is not clear how many biological replicates are analyzed. It seems that for the quantification, each cell is considered as a biological replicate, which is not correct. The authors should average the result for all the cells from the same culture and consider this as a single biological replicate.

Response:

We thank the reviewer for pointing this out. We agree that individual cells should not be treated as independent biological replicates in the cell morphology analysis.

In response to this comment, we reanalyzed the data in Fig. EV2C using the independent culture, rather than individual cells, as the biological replicate. Specifically, multiple cells were quantified from each independent culture, and values from all cells within the same culture were averaged to generate one value per culture. Each culture was then treated as one biological replicate for statistical analysis. We have revised Fig. EV2C, the Figure EV2 legend, and the Methods section accordingly.

The revised Figure EV2C legend now states:

C Quantification of cell morphology parameters, including cell area, circularity, and aspect ratio, in siDysf- and siControl-transfected J774A.1 cells under untreated or IFN-γ-stimulated conditions. IFN-γ was used at 100 ng/mL. Multiple cells were quantified from each independent culture, and values from cells within the same culture were averaged before statistical analysis. Each dot represents one independent culture ($n = 4$).

The revised Methods section now states:

Line # 499-501

For cell morphology analysis, multiple cells were measured from each independent culture. Values from cells within the same culture were averaged before statistical analysis, and each independent culture was treated as one biological replicate.

7) In Figure 5, as an in vivo validation, the authors first mesure Dysf mRNA and protein levels in bone marrow-derived monocytes from young or old mice. Then, the authors measure DYSF levels in macrophages on muscle sections (Figure 5D-E). However, it is not clear what the authors quantify as they mention signal intensity in the Figure legend but show a percentage in the graph.

Response:

We thank the reviewer for pointing out this ambiguity. We agree that the previous description was unclear because the Figure legend referred to signal intensity, whereas the graph showed a percentage-based quantification.

In the revised manuscript, we have corrected the Figure 5 legend and the y-axis label to clearly indicate what was quantified. In Fig. 5D–E, we did not quantify total Dysferlin signal intensity. Instead, we quantified the percentage of F4/80^{+}Dysferlin^{+} macrophage-lineage cells among total DAPI-positive nuclei in skeletal muscle sections.

Specifically, F4/80^{+}Dysferlin^{+} cells and total DAPI-positive nuclei were counted in multiple fields from each muscle section. The percentage of F4/80^{+}Dysferlin^{+} cells among total DAPI-positive nuclei was calculated for each mouse, and each mouse was treated as one biological replicate for statistical analysis.

We have revised the Figure 5 legend as follows:

D Representative immunofluorescence images of frozen soleus muscle sections from young and aged mice stained for F4/80 and Dysferlin; nuclei were counterstained with DAPI. Scale bar, 50 µm.

E Quantification of the percentage of F4/80^{+}Dysferlin^{+} macrophage-lineage cells among total DAPI-positive nuclei in skeletal muscle sections. Values from multiple fields within the same muscle section were averaged before statistical analysis, and each mouse was treated as one biological replicate. Each dot represents one mouse.

We also revised the Results text to avoid describing this analysis as a measurement of DYSF signal intensity.

8) In Figure EV6C-FMoreover, it is not clear what the authors consider as a biological replicate as they mention n=3 in the legend but show more individual data points in the graphs. The results obtained from different fields within the same muscle should be averaged and considered as only one biological replicate.

Response:

We thank the reviewer for pointing out this important issue. We agree that different fields from the same muscle section should not be treated as independent biological replicates.

In response to this comment, we reanalyzed the quantification in Fig. EV6C–F using the mouse, rather than individual fields, as the biological replicate. Specifically, multiple fields were analyzed from each muscle section, and values from fields within the same mouse were averaged to generate one value per mouse. Each mouse was then treated as one biological replicate for statistical analysis. We have revised Fig. EV6C–F so that each dot represents one mouse, consistent with the stated sample size.

We have also revised the Figure EV6 legend and Methods section to clarify the statistical unit used for immunofluorescence quantification.

The revised Figure EV6 legend now states:

B Quantification of CD80-positive and CD206-positive cells or immunofluorescence-positive area in skeletal muscle sections. Multiple fields were analyzed from each muscle section, and values from fields within the same mouse were averaged before statistical analysis. Each dot represents one mouse, which was treated as one biological replicate. Each dot represents one mouse.

The revised Methods section now states:

Line # 573-575

For immunofluorescence quantification of skeletal muscle sections, multiple fields were analyzed from each mouse. Values from fields within the same mouse were averaged before statistical analysis, and each mouse was treated as one biological replicate.

9) There are variations between blots for DYSF protein. Sometimes there is only one band (Fig EV1A), sometimes there are 2 bands (FigEV2A). The authors should discuss these differences and explain which bands are considered for their quantification.

Response:

We thank the reviewer for pointing out this important issue. We agree that the DYSF immunoblot pattern was not sufficiently explained in the original manuscript.

DYSF is a large membrane-associated protein, and the anti-DYSF antibody detected either a single major band or two closely migrating DYSF-immunoreactive bands in the expected high-molecular-weight range, depending on the experimental condition and sample preparation. Because both bands appeared within the expected DYSF-positive region and changed in the same direction after DYSF overexpression

or Dysf knockdown, we quantified the total intensity of these DYSF-immunoreactive bands when two bands were detected.

We have added the following clarification to the relevant Figure EV1A legends:

A Immunoblot analysis of DYSF protein expression in J774A.1 macrophages transiently transfected with a DYSF-3HA expression vector (DYSF) or pcDNA3.1 empty vector (Control). β-actin (Actin) was used as a loading control. DYSF protein abundance was quantified from the DYSF-immunoreactive band(s) in the expected high-molecular-weight range and normalized to β-actin. Each dot represents one independent culture (n = 3).

10) The authors should not use the M1/M2 classification when they refer to the macrophage inflammatory state in muscle in vivo as it corresponds to a classification established in vitro which does not recapitulate the subpopulations status in vivo.

Response:

We thank the reviewer for this important comment. We agree that the M1/M2 classification is an in vitro framework and should not be used to define macrophage states in skeletal muscle in vivo. In response, we revised the terminology throughout the manuscript. We now describe the in vitro phenotypes as "inflammatory macrophage-like activation," "inflammatory macrophage-associated genes," and "alternative macrophage-associated markers," and we describe the in vivo staining data as "CD80-positive cells," "CD206-positive cells," or "macrophage-associated marker immunoreactivity," rather than M1- or M2-type macrophages.

We also revised the interpretation of Fig. EV6C–F to clarify that CD80 and CD206 single staining does not definitively identify macrophage subsets in skeletal muscle sections. In addition, we added F4/80/Dysferlin co-immunostaining in Fig. 5D–E to more directly identify DYSF-expressing macrophage-lineage cells in aged skeletal muscle. The Discussion has been revised to state that macrophage activation states in vivo are heterogeneous and cannot be fully represented by a simple M1/M2 dichotomy.

Reviewer #2:

Reviewer Comments:

This manuscript presents compelling evidence for a novel role of DYSF in regulating macrophage polarization, Ca^{2+} signaling, and myoblast fusion. The findings are mechanistically interesting and physiologically relevant, especially in the context of aging. However, key mechanistic links (Ca^{2+} → STAT1), validation in primary cells, in vivo functional relevance, and specificity of STAT1 activation need further investigation. Addressing these points will significantly strengthen the manuscript.

Response to Reviewer #2:

We thank the reviewer for the positive evaluation of our manuscript and for the constructive comments. We agree that the Ca^{2+}–STAT1 mechanistic link, specificity of STAT1 activation, loss-of-function evidence, and in vivo relevance required further clarification.

In response, we added Ca^{2+} inhibition experiments using BTP2, SKF96365, and Ca^{2+}-free/EGTA conditions, which reduced DYSF-associated STAT1 Ser727 phosphorylation and STAT1 nuclear localization. We also added STAT1 knockdown experiments showing that DYSF-induced STAT1-responsive inflammatory gene expression was attenuated by siSTAT1. To address signaling specificity, we examined STAT3, STAT6, and NF-κB p65 phosphorylation and found no marked changes under the same conditions.

We further strengthened the loss-of-function evidence by adding Dysf knockdown experiments, showing reduced inflammatory gene expression, cytokine production, Ca^{2+} responses, STAT1 phosphorylation, and macrophage migration. In the macrophage–myoblast co-culture system, Dysf knockdown in DYSF-overexpressing macrophages restored myogenic marker expression and Myh1-positive myotube formation.

To improve *in vivo* relevance, we added F4/80/Dysferlin co-immunostaining and showed that F4/80⁺Dysferlin⁺ macrophage-lineage cells accumulate in aged skeletal muscle. We also revised the manuscript to avoid overinterpreting *in vivo* macrophage states using a strict M1/M2 classification.

We agree that validation in primary macrophages and macrophage-specific Dysf loss-of-function models would further strengthen the study. Because these experiments require additional sample acquisition, breeding, and long-term aging or injury studies, we have added this point to the Discussion as a limitation and future direction.

1) STAT1 inhibition (e.g., fludarabine or siSTAT1) → cytokine output?

Response:

We thank the reviewer for this important suggestion. We agree that demonstrating the effect of STAT1 inhibition on cytokine output is important to strengthen the functional link between DYSF-associated STAT1 signaling and inflammatory cytokine production.

In response, we performed STAT1 knockdown experiments using siRNA targeting Stat1 in DYSF-overexpressing J774A.1 macrophages. We chose siSTAT1 rather than pharmacological inhibition with fludarabine to minimize potential off-target or cytotoxic effects of chemical inhibition. Efficient STAT1 knockdown was confirmed by immunoblotting (Fig EV4H). We then measured TNF-α and IL-6 levels in the conditioned medium by ELISA.

STAT1 knockdown reduced DYSF-induced TNF-α and IL-6 secretion, indicating that STAT1 contributes to the cytokine output induced by DYSF overexpression. These data have been added to Fig. EV4I and the corresponding Results section.

The revised Results section now states:

Line # 250-254

To further assess whether STAT1 contributes to DYSF-induced cytokine output, we measured TNF-α and IL-6 levels in conditioned media after STAT1 knockdown. STAT1 knockdown attenuated TNF-α and IL-6 secretion from DYSF-overexpressing macrophages (Fig. EV4I). These results suggest that DYSF promotes inflammatory macrophage-associated gene expression through Ca^{2+}-dependent STAT1 Ser727-associated signaling.

Together, these results support the functional involvement of STAT1 in DYSF-associated inflammatory macrophage activation and cytokine production.

2) The analysis of STAT1 signaling is limited and raises concerns:

Canonical STAT1 activation is typically defined by phosphorylation at Tyr701, yet the study emphasizes Ser727 phosphorylation and unphosphorylated STAT1.

Functional evidence of STAT1 transcriptional activity is not provided (e.g., reporter assays or broader target gene analysis).

Response:

We thank the reviewer for this important comment. We agree that canonical STAT1 activation is primarily defined by phosphorylation at Tyr701, whereas Ser727 phosphorylation and unphosphorylated STAT1 represent additional regulatory modes of STAT1 signaling. In the revised manuscript, we have therefore modified our terminology and no longer describe the observed changes simply as "STAT1 activation." Instead, we refer to "STAT1 Ser727-associated signaling," "STAT1 nuclear accumulation," and "STAT1-responsive gene expression."

To address this point experimentally, we examined both STAT1 Tyr701 and Ser727 phosphorylation. DYSF overexpression preferentially increased STAT1 Ser727 phosphorylation and STAT1 nuclear localization, whereas STAT1 Tyr701 phosphorylation was not markedly enhanced compared with control cells. These findings indicate that DYSF does not simply activate canonical IFN-γ–STAT1 signaling but rather enhances a Ser727-associated regulatory component of STAT1 signaling.

To provide functional evidence of STAT1 transcriptional output, we expanded the downstream analysis by examining STAT1-responsive genes, including *Cxcl10, Irf1, Nos2/iNOS, and Socs1*. DYSF overexpression increased the expression of these genes, whereas Ca^{2+} entry inhibition with BTP2 or SKF96365 attenuated their induction. Furthermore, STAT1 knockdown reduced DYSF-induced STAT1-responsive inflammatory gene expression, supporting the functional involvement of STAT1 in this pathway.

Although we did not perform a STAT1 reporter assay in the current revision, the combination of STAT1 phosphorylation analysis, nuclear localization, broader STAT1-responsive gene analysis, and STAT1 knockdown provide functional evidence that DYSF enhances STAT1-associated inflammatory signaling. We have revised the Results and Discussion to clarify this interpretation and to avoid overstatement.

The revised Results section now states:

Line # 206-214

Immunoblot analysis revealed that DYSF overexpression affected the levels of unphosphorylated STAT1 (U-STAT1) and the phosphorylation of STAT1 at Ser727, whereas phosphorylation of STAT1 at Tyr701 was not markedly enhanced by DYSF overexpression compared with control cells (Fig. 4A, B). To further explore the involvement of DYSF in the STAT1 signaling pathway, we assessed U-STAT1 and STAT1 phosphorylation in *DYSF* siRNA-transfected J774A.1 cells. Compared with mock and control siRNA transfected cells, *DYSF*-knockdown cells exhibited reduced U-STAT1 expression and decreased STAT1 Ser727 and Tyr 701 phosphorylation under IFN-γ-stimulated conditions. (Fig. 4C, D). These results suggest that DYSF preferentially modulates a Ser727-associated regulatory component of STAT1 signaling rather than simply enhancing canonical Tyr701-dependent STAT1 activation.

The revised Discussion section now states:

Line # 372-379

Our data indicate that DYSF overexpression preferentially enhanced STAT1 Ser727 phosphorylation without markedly increasing canonical IFN-γ-dependent STAT1 Tyr701 phosphorylation, whereas Dysf knockdown reduced both STAT1 Tyr701 and Ser727 phosphorylation under IFN-γ-stimulated conditions. These findings suggest that DYSF contributes to STAT1 signaling through both regulation of endogenous STAT1 abundance and enhancement of STAT1 Ser727-associated signaling. Importantly, DYSF overexpression did not markedly alter STAT3, STAT6, or NF-κB p65 phosphorylation under the same conditions, supporting a preferential association of DYSF with STAT1-associated signaling in this experimental setting.

3) Can these findings be validated in primary human monocyte-derived macrophages to strengthen translational relevance?

Response:

We thank the reviewer for this important suggestion. We agree that validation in primary human monocyte-derived macrophages would further strengthen the translational relevance of our findings.

Although we were not able to complete experiments using primary human monocyte-derived macrophages within the current revision timeframe, we have strengthened the human relevance of the study in several ways. First, we examined DYSF expression in human U937 monocytic cells during PMA-induced macrophage-like differentiation and after cytokine stimulation. These experiments showed that DYSF expression increases during human macrophage-like differentiation and is further enhanced by IFN-γ stimulation. Second, we analyzed human CD14-positive cell data and found that DYSF expression increases with aging, supporting the relevance of DYSF expression in human monocyte/macrophage-lineage cells.

We have revised the Discussion to explicitly acknowledge the lack of primary human macrophage validation as a limitation.

The revised text now states:

Line # 452-456

Finally, although our findings were supported by human U937 macrophage-like cells and human CD14-positive cell data, validation using primary human monocyte-derived macrophages will be required to further establish the translational relevance of DYSF-dependent Ca^{2+}–STAT1-associated inflammatory signaling. Further studies will also be required to determine whether this pathway contributes to tissue dysfunction under age-associated or disease-related conditions.

We believe these revisions provide a more balanced interpretation while identifying primary human macrophage validation as an important future direction.

In vivo relevance

4) The manuscript shows DYSF accumulation in aged mouse macrophages and skeletal muscle. Can the authors demonstrate functional consequences in vivo? Does DYSF drive aging phenotype?

Response:

We thank the reviewer for this important comment. We agree that demonstrating the *in vivo* functional consequence of macrophage DYSF accumulation would be important to determine whether DYSF directly drives age-associated muscle phenotypes.

In the current study, our aged-mouse data show that DYSF expression is increased in monocyte/macrophage-lineage cells and that F4/80^{+}Dysferlin^{+} macrophage-lineage cells accumulate in aged skeletal muscle. However, we agree that these observations are associative and do not establish that macrophage DYSF directly drives the aging phenotype *in vivo*. We have therefore revised the manuscript to avoid overinterpreting the aged-muscle data as direct evidence of causality.

To strengthen the functional aspect of the study, we added loss-of-function experiments in the macrophage–myoblast co-culture system. Dysf knockdown in DYSF-overexpressing macrophages restored myogenic marker expression and Myh1-positive myotube formation, supporting a functional role of macrophage DYSF in suppressing myogenic differentiation *in vitro*. Nevertheless, we agree that macrophage-specific *in vivo* loss-of-function models will be required to determine whether DYSF contributes directly to age-associated muscle dysfunction.

The revised Discussion now states:

Line # 422-429

However, several limitations should be noted. First, although our aged-mouse tissue analyses demonstrate the accumulation of DYSF-expressing F4/80-positive macrophage-lineage cells in skeletal muscle, they do not establish in vivo causality. Macrophage-specific Dysf loss-of-function models, bone marrow transplantation, and muscle injury or aging models will be required to determine whether macrophage DYSF directly contributes to impaired muscle regeneration *in vivo.* In addition, although CSA was analyzed using the mouse as the biological replicate, complete whole-section quantification was not performed. Analyses of whole muscle sections and additional muscles will be needed to confirm the robustness and muscle-type specificity of the apparent CSA changes.

We have also revised the Results and Abstract to state that DYSF-expressing macrophage-lineage cells are associated with aged skeletal muscle remodeling, rather than concluding that DYSF drives the aging phenotype *in vivo*.

5) Does DYSF knockdown in aged mice alter macrophage polarization or muscle inflammation? Does overexpression in vivo impact muscle regeneration after injury?

Response:

We thank the reviewer for this important comment. We agree that macrophage-specific DYSF knockdown in aged mice and *in vivo* DYSF overexpression followed by muscle injury would provide definitive evidence for the functional role of DYSF in muscle inflammation and regeneration.

These experiments were not completed in the current revision because they require additional in vivo delivery or genetic models, aging or injury protocols, and longitudinal functional analyses beyond the revision timeframe. Therefore, we have revised the manuscript to avoid concluding that DYSF directly drives aging-related muscle inflammation or regeneration defects in vivo.

To strengthen the functional evidence, we added Dysf knockdown experiments in the macrophage–myoblast co-culture system. Dysf knockdown in DYSF-overexpressing macrophages restored myogenic marker expression and Myh1-positive myotube formation. We also added F4/80/Dysferlin co-immunostaining showing accumulation of DYSF-expressing macrophage-lineage cells in aged skeletal muscle.

We now state in the Discussion that future studies using macrophage-specific Dysf loss-of-function models in aged mice and in vivo DYSF gain-of-function approaches combined with muscle injury models will be required to determine whether macrophage DYSF causally regulates muscle inflammation and regeneration *in vivo*.

6) DYSF and M2 polarization

The data indicate DYSF has a weak effect on M2 polarization. Has the effect of DYSF knockdown on M2 polarization under IL-4 stimulation been fully explored? Could DYSF actively suppress M2 differentiation?

Response:

We thank the reviewer for this important comment. We agree that DYSF overexpression did not strongly enhance IL-4-induced alternative macrophage-associated markers, suggesting that DYSF does not broadly promote an alternative macrophage-like phenotype.

To address whether endogenous DYSF may instead suppress IL-4-induced alternative macrophage-associated responses, we examined the effect of Dysf knockdown under IL-4-stimulated conditions. Dysf knockdown enhanced the expression of IL-4-induced alternative macrophage-associated markers, *Arg1,*

IL-10, and CD206, **compared with control siRNA-transfected cells. These data have been added to Fig. EV2D and the corresponding Results section.**

Based on these findings, we revised the text to state that DYSF preferentially promotes inflammatory macrophage-associated responses and may limit the acquisition of IL-4-induced alternative macrophage-associated features. However, we have avoided concluding that DYSF definitively suppresses M2 differentiation, because our analysis was based on representative marker expression rather than a comprehensive assessment of alternative macrophage function.

The revised Results section now states:

Line # 145-149

In contrast, *Dysf* knockdown enhanced IL-4-induced alternative macrophage-associated gene expression, suggesting that DYSF may limit the acquisition of an IL-4-induced alternative macrophage-associated phenotype (Fig. EV2D). Together, these gain- and loss-of-function experiments indicate that DYSF modulates macrophage activation states by promoting inflammatory macrophage-associated responses.

7) Impact on myogenesis

Co-culture experiments suggest DYSF-overexpressing macrophages reduce myoblast fusion via TNF-α and IL-6. Could the authors use neutralizing antibodies or cytokine inhibitors to definitively show that these cytokines mediate the effect?

Response:

We thank the reviewer for this important suggestion. We agree that functional inhibition of TNF-α and IL-6 is necessary to determine whether these cytokines mediate the suppressive effect of DYSF-overexpressing macrophages on myogenic differentiation.

In response, we performed neutralization experiments using anti-TNF-α or anti-IL-6 antibodies in the macrophage–myoblast co-culture system. C2C12 myoblasts were co-cultured with DYSF-overexpressing or control J774A.1 macrophages using a Boyden chamber insert system, and neutralizing antibodies were added during myogenic differentiation. Neutralization of either TNF-α or IL-6 partially restored the expression of myogenic differentiation markers, including ***Ckm, Myhc, and Myog*****, which were otherwise suppressed by co-culture with DYSF-overexpressing macrophages. These data have been added to Fig. 6B and the corresponding Results section.**

We have revised the text to state that TNF-α and IL-6 contribute, at least in part, to the suppression of myogenic differentiation by DYSF-overexpressing macrophages. However, because neutralization produced partial rescue, we have avoided concluding that these cytokines are the sole mediators of the effect.

The revised Results section now states:

Line # 309-312

Notably, neutralization of TNF-α or IL-6 restored the expression of all three genes in the co-cultured C2C12 cells. These findings suggest that proinflammatory cytokines released from DYSF-overexpressing macrophages contribute to the suppression of myogenic differentiation.

The revised Discussion now states:

Line # 412-416

Consistent with this interpretation, neutralization of TNF-α or IL-6 partially restored the expression of myogenic differentiation markers.

8) Most experiments use single overexpression or knockdown conditions. Would a dose-dependent study of DYSF expression strengthen the link between DYSF levels, macrophage polarization, and Ca^{2+} influx?

Response:

We thank the reviewer for this valuable suggestion. We agree that a dose-dependent analysis of DYSF expression would further strengthen the relationship between DYSF abundance, macrophage inflammatory activation, and Ca^{2+} influx.

In the current revision, we did not perform a systematic dose-response experiment using graded levels of DYSF expression, because establishing stable and reproducible intermediate DYSF expression levels would require additional optimization beyond the revision timeframe. However, we strengthened the causal link by analyzing both gain- and loss-of-function conditions. DYSF overexpression enhanced inflammatory macrophage-associated gene expression, cytokine production, Ca^{2+} influx, STAT1 Ser727-associated signaling, and suppression of myogenic differentiation. Conversely, Dysf knockdown reduced inflammatory gene expression, cytokine secretion, Ca^{2+} responses, STAT1 phosphorylation, and macrophage migration, and restored myogenic differentiation in the co-culture system.

Thus, although a formal dose-response study was not included, the bidirectional effects of DYSF overexpression and Dysf knockdown support a relationship between DYSF levels and macrophage inflammatory function.

9) STAT1 specificity

DYSF may affect multiple signaling pathways. Did the authors test whether other STAT family members (STAT3/STAT6) or NF-κB signaling are influenced by DYSF? This would clarify specificity to STAT1.

Response:

We thank the reviewer for this important comment. We agree that DYSF may influence multiple inflammatory signaling pathways and that examining additional pathways is necessary to clarify the relative specificity of STAT1-associated signaling.

In response, we examined the phosphorylation levels of STAT3, STAT6, and NF-κB p65 in DYSF-overexpressing and control J774A.1 macrophages, with or without cytokine stimulation. Under these experimental conditions, DYSF overexpression did not markedly alter STAT3, STAT6, or NF-κB p65 phosphorylation compared with control cells. These data have been added to Supplementary Fig. S4A, B and the corresponding Results section.

The revised Results section now states:

Line # 215-219

To address whether DYSF broadly affects other macrophage-associated signaling pathways, we also examined STAT3, STAT6, and NF-κB p65 phosphorylation. Under these conditions, DYSF overexpression did not markedly alter the phosphorylation levels of STAT3, STAT6, or NF-κB p65 compared with control cells, with or without IFN-γ or IL-4 stimulation (Fig. S4A, B). These results further support the preferential association of DYSF with STAT1 Ser727-associated signaling in this experimental setting.

The revised Dissucission section now states:

Line # 362-375

The balance among STAT1-, STAT3-, and STAT6-associated signaling pathways plays an important role in regulating macrophage activation programs (Sica & Mantovani, 2012). Inflammatory macrophage-associated activation is generally linked to STAT1 and NF-κB signaling, whereas STAT3 and STAT6 are more closely associated with anti-inflammatory or alternative macrophage-associated responses. In our

experimental setting, DYSF preferentially enhanced STAT1 Ser727-associated signaling, whereas STAT3, STAT6, and NF-κB p65 phosphorylation were not markedly altered. These findings suggest that DYSF is preferentially associated with STAT1 Ser727-related inflammatory signaling under the conditions tested.

In our experimental setting, DYSF preferentially enhanced STAT1 Ser727-associated signaling, whereas STAT3, STAT6, and NF-κB p65 phosphorylation were not markedly altered. These findings suggest that DYSF is preferentially associated with STAT1 Ser727-related inflammatory signaling under the conditions tested.In our experimental setting, DYSF preferentially enhanced STAT1 Ser727-associated signaling, whereas STAT3, STAT6, and NF-κB p65 phosphorylation were not markedly altered. These findings suggest that DYSF is preferentially associated with STAT1 Ser727-related inflammatory signaling under the conditions tested.

We have also revised the Discussion to avoid overstatement. We now state that DYSF preferentially enhances STAT1 Ser727-associated signaling under the conditions tested, rather than concluding that DYSF is exclusively specific to STAT1.

Comments

10) Morphological analysis (cell area, circularity) should not be overinterpreted as evidence of differentiation.

Response:

We thank the reviewer for this important comment. We agree that changes in cell area, circularity, and aspect ratio alone do not provide definitive evidence of macrophage differentiation or activation state.

In response, we revised the manuscript to avoid overinterpreting the morphological analysis. We now describe these data as supportive evidence of activation-associated morphological changes, rather than as evidence of macrophage differentiation. The conclusions regarding DYSF-dependent inflammatory macrophage-associated activation are instead based primarily on inflammatory gene expression, cytokine production, surface marker analysis, Ca^{2+} responses, STAT1-associated signaling, and loss-of-function experiments.

The revised Results section now states:

Line # 122-132

We then measured cell proliferation, cell area, aspect ratio and circularity to assess the characterization of DYSF-overexpressing cells. Compared with control cells, DYSF-overexpressing cells showed slight reduced cell proliferation but exhibited morphological features associated with macrophage activation and differentiation (Fig. EV1C). Cell area and aspect ratio increased with differentiation, whereas circularity decreases (Moss *et al*, 2024). Consistent with these features, DYSF-overexpressing cells were larger in size, elongated and less circular than control cells (Fig. 2A, B). We next performed knockdown experiments in J774A.1 using small interfering RNA (siRNA) targeting DYSF (Fig. EV2A). Under IFN-γ-stimulated conditions, Dysf knockdown reduced cell area and aspect ratio, whereas circularity was conversely increased (Fig. EV2B, C). These findings indicate that DYSF is associated with morphological remodeling of macrophage-like cells, although cell morphology alone cannot define macrophage differentiation or activation state.

We believe these revisions address the reviewer's concern and provide a more cautious interpretation of the morphological data.

11) Statistical details (n values, replicates) should be consistently reported across all figures.

Response:

We thank the reviewer for this important comment. We agree that the definition of n values and biological replicates was not consistently described across the original figure legends.

In response, we carefully revised all figure legends and the Methods section to consistently report the number of independent biological replicates, the statistical unit used for analysis, and how technical measurements were handled. For cell-based assays, each dot now represents one independent culture or independent experiment. For tissue analyses, each dot represents one mouse. For image-based quantification, including cell morphology, immunofluorescence intensity, myofiber CSA, and myotube formation, values from multiple cells, fields, or fibers within the same independent culture, dish, or mouse were averaged before statistical analysis. These averaged values were then used as biological replicates.

The revised Methods section now states:

Line # 598-602

For all quantitative analyses, the biological replicate and statistical unit are specified in the corresponding figure legends. For cell-based experiments, independent cultures or independent experiments were treated as biological replicates. For mouse tissue analyses, each mouse was treated as one biological replicate. When multiple cells, fields, myofibers, or myotubes were quantified within the same culture, dish, or mouse, these values were averaged before statistical analysis to avoid pseudo-replication.

We have also revised the figure legends to indicate the exact n values and to clarify what each dot represents in each graph. These revisions improve the transparency and consistency of the statistical reporting throughout the manuscript.

12) Some claims in the Discussion (e.g., direct regulation of STAT1 by Ca^{2+}) are overstated relative to the data.

Response:

We thank the reviewer for this important comment. We agree that our previous wording overstated the mechanistic relationship between Ca^{2+} signaling and STAT1. Although our data show that Ca^{2+} entry inhibition reduces DYSF-associated STAT1 Ser727 phosphorylation and STAT1 nuclear localization, they do not demonstrate direct biochemical regulation of STAT1 by Ca^{2+}.

In response, we revised the Discussion and Results to use more cautious language. We now state that Ca^{2+} influx contributes to DYSF-associated STAT1 Ser727 phosphorylation and nuclear localization, rather than directly regulating STAT1. We also revised the manuscript to avoid implying that Ca^{2+} acts directly on STAT1 or that this pathway is the only mechanism linking DYSF to inflammatory macrophage-associated gene expression.

We also revised the manuscript title of this section to avoid overstatement and to better reflect the scope of our data.

Before:

Ca^{2+} directly regulates STAT1 activation downstream of DYSF.

After:

DYSF Enhances Ca^{2+}-dependent STAT1 Ser727 Phosphorylation and STAT1-responsive Gene Expression.

The revised Discussion now states:

Line # 385-393

Consistent with this concept, DYSF overexpression enhanced intracellular Ca^{2+} responses, whereas Dysf knockdown attenuated Ca^{2+} influx. Moreover, Ca^{2+} entry inhibition by BTP2, SKF96365, or Ca^{2+}-free/EGTA conditions reduced DYSF-associated STAT1 Ser727 phosphorylation and STAT1 nuclear localization. These results support the idea that DYSF-associated STAT1 signaling depends, at least in part, on Ca^{2+} influx. Because A23187 is a Ca^{2+} ionophore, these experiments indicate that increased intracellular Ca^{2+} is sufficient to enhance STAT1-associated signaling in DYSF-overexpressing macrophages, but they do not by themselves establish a physiological receptor-mediated Ca^{2+} pathway. Further studies using receptor-mediated stimulation will be important to define how DYSF regulates physiological Ca^{2+} signaling in macrophages.

We believe these revisions provide a more balanced interpretation of the mechanistic data.

13) Clarify whether sex-specific differences were observed in aging experiments.

Response:

We thank the reviewer for this important comment. In the revised manuscript, we clarified the analysis of sex-specific differences in the aging experiments.

We analyzed male and female mice separately and also assessed the interaction between age and sex using two-way ANOVA. Dysf mRNA expression and DYSF protein levels in bone marrow-derived CD11b-positive monocyte/macrophage-lineage cells were increased in aged mice compared with young mice in both males and females. Similarly, F4/80⁺Dysferlin⁺ macrophage-lineage cells accumulated in aged skeletal muscle in both sexes. Two-way ANOVA did not reveal a significant age × sex interaction for these parameters, indicating that the age-associated increase in macrophage-lineage DYSF expression was observed in both sexes without clear evidence of sex-specific regulation.

After reanalysis using the mouse as the biological replicate, an apparent increase in myofiber CSA was also observed in aged mice of both sexes, and no significant age × sex interaction was detected. We have revised the Results to state that aging-associated DYSF accumulation and skeletal muscle remodeling were observed in both males and females, while avoiding overinterpretation of sex-specific effects.

The revised Results section now states:

Line # 262-271

To validate these findings, we isolated monocytes from the bone marrow of young (6-8-week-old, 5 females/5 males) and aged (18-30-month-old, 5 females/5 males) mice and cultured monocyte-derived CD11b-positive macrophage-lineage cells. We observed that compared with that in young mice, *DYSF* mRNA expression in CD11b-positive cells was upregulated in both male and female aged mice (Fig. 5A). Furthermore, DYSF protein levels were significantly increased in CD11b-positive cells from aged mice in both males and females (Fig. 5B, C), suggesting that aging is associated with increased DYSF expression in monocyte/macrophage-lineage cells. However, Two-way ANOVA did not reveal a significant age × sex interaction, indicating that the age-associated increase in DYSF expression was observed in both sexes, suggest that macrophage-lineage DYSF accumulation is associated with aging in both sexes, although larger cohorts will be required to evaluate subtle sex-dependent differences.

13) Improve clarity in figure legends, particularly regarding normalization and quantification methods.

Response:

We thank the reviewer for this important comment. We agree that several figure legends in the original manuscript did not provide sufficient detail regarding normalization procedures and quantification methods.

In response, we carefully revised the figure legends throughout the manuscript to clarify how each quantitative value was obtained. For immunoblot analyses, we now specify the loading control used for normalization and, where applicable, which DYSF-immunoreactive band(s) were quantified. For qPCR analyses, we indicate that expression levels were normalized to the indicated housekeeping gene and presented relative to the control condition. For immunofluorescence and image-based analyses, we now define whether signal intensity, positive cell number, positive area, or percentage of positive cells was quantified. We also clarified the denominator used for percentage-based analyses, such as the percentage of F4/80^{+}Dysferlin^{+} cells among total DAPI-positive nuclei.

We further revised all figure legends to define the biological replicate and statistical unit. For cell-based experiments, each dot represents one independent culture or experiment. For tissue analyses, each dot represents one mouse. Values from multiple cells, fields, fibers, or myotubes within the same biological replicate were averaged before statistical analysis.

We believe these revisions improve the transparency and reproducibility of the quantitative analyses.

14) The manuscript presents a comprehensive and novel study linking DYSF to macrophage polarization and myogenesis. The experiments are well designed and mechanistic insights are strong. However, to fully establish causality and translational relevance, additional experiments in primary human macrophages, in vivo functional validation, and more rigorous testing of STAT1 specificity would strengthen the conclusions.

Response:

We thank the reviewer for the positive assessment of our study and for recognizing the novelty and mechanistic significance of linking DYSF to macrophage inflammatory activation and myogenesis. We also agree that primary human macrophage validation, *in vivo* functional studies, and further testing of STAT1 specificity would strengthen the conclusions.

In response, we added new experiments to strengthen the mechanistic evidence. We examined Ca^{2+} entry inhibition, STAT1 knockdown, STAT1-responsive inflammatory gene expression, and cytokine output. We also tested STAT3, STAT6, and NF-κB p65 phosphorylation and found no marked changes under the conditions examined, supporting a preferential association of DYSF with STAT1 Ser727-associated signaling.

To improve *in vivo* relevance, we added F4/80/Dysferlin co-immunostaining and clarified that DYSF-expressing macrophage-lineage cells accumulate in aged skeletal muscle. However, we agree that these data do not establish in vivo causality. We have therefore revised the manuscript to avoid overstatement and added primary human macrophage validation, macrophage-specific Dysf loss-of-function models, and injury/aging models as important future directions.

We believe that the revised manuscript now presents a more balanced conclusion, with strengthened mechanistic data and clearer acknowledgement of the remaining limitations.

Reviewer #3:

The manuscript by Tominaga et al. explores the role of dysferlin (DYSF) in macrophage differentiation and function, proposing that DYSF promotes inflammatory macrophage polarization through a Ca^{2+}-dependent activation of STAT1 signaling. The authors further suggest that DYSF-enriched macrophages impair myogenic differentiation and that DYSF expression increases with aging, potentially linking this pathway to age-associated muscle dysfunction. The topic is interesting and potentially impactful, particularly given the limited understanding of dysferlin function outside muscle membrane repair. However, although the study presents intriguing observations, several conceptual and experimental limitations substantially weaken the strength of the conclusions.

Response to Reviewer #3:

We thank the reviewer for the careful evaluation of our manuscript and for recognizing the potential importance of DYSF function in macrophages beyond muscle membrane repair. We also appreciate the reviewer's critical comments regarding the conceptual and experimental limitations.

In response, we substantially revised the manuscript to avoid overstatement and to strengthen the mechanistic and functional evidence. We revised the terminology throughout the manuscript and no longer interpret macrophage phenotypes using a strict M1/M2 framework, particularly *in vivo*. We also modified the description of the Ca^{2+}–STAT1 pathway from "STAT1 activation" to the more cautious term "STAT1 Ser727-associated signaling."

We added Ca^{2+} inhibition experiments, STAT1 knockdown experiments, STAT1-responsive gene analyses, and signaling specificity analyses including STAT3, STAT6, and NF-κB p65. We also strengthened loss-of-function evidence using Dysf knockdown, including macrophage–myoblast co-culture rescue experiments. To improve in vivo relevance, we added F4/80/Dysferlin co-immunostaining in aged skeletal muscle.

Finally, we revised the Discussion to clearly state remaining limitations, including the need for primary human macrophage validation and macrophage-specific in vivo Dysf loss-of-function models. These revisions provide a more balanced interpretation of the role of DYSF in macrophage inflammatory activation and myogenic suppression.

Major Revisions

The central claims of the manuscript, namely that DYSF drives inflammatory macrophage polarization and negatively affects myogenesis, are supported almost entirely by in vitro systems, including macrophage cell lines and co-culture assays with C2C12 myoblasts. While the authors include in vivo data showing increased DYSF expression in aged macrophages, these observations are purely descriptive and do not establish causality. There is no demonstration that DYSF functionally contributes to macrophage polarization or muscle regeneration in vivo. Given the physiological relevance emphasized throughout the manuscript, this represents a critical gap that must be addressed.

1. The authors should incorporate loss-of-function approaches in vivo to substantiate the proposed role of DYSF in tissue contexts. Macrophage-specific Dysf deletion using conditional knockout strategies would allow direct testing of whether DYSF is required for inflammatory macrophage polarization during muscle injury or aging. Alternatively, bone marrow transplantation experiments using Dysf-deficient donor cells could assess the contribution of macrophage DYSF to muscle regeneration outcomes. Muscle injury models, such as cardiotoxin or freeze injury, would provide physiologically relevant contexts in which to evaluate the impact of macrophage DYSF on tissue repair, inflammation resolution, and myogenic differentiation. Without such in vivo functional validation, the manuscript remains largely descriptive and the mechanistic claims lack the necessary support for publication.

Response:

We thank the reviewer for this important and constructive comment. We fully agree that macrophage-specific in vivo loss-of-function experiments, bone marrow transplantation, and muscle injury models would provide the most definitive evidence to determine whether macrophage DYSF causally regulates muscle inflammation, macrophage activation states, and muscle regeneration *in vivo*.

In the current revision, we were not able to generate macrophage-specific Dysf conditional knockout mice or perform bone marrow transplantation followed by cardiotoxin or freeze injury, because these experiments require new animal cohorts, breeding or transplantation procedures, injury models, and

longitudinal regeneration analyses that extend beyond the timeframe of the revision. We therefore agree that our aged-mouse data should be interpreted as associative rather than causal.

In response to this concern, we substantially revised the manuscript to avoid overstatement. We no longer conclude that DYSF drives age-associated muscle dysfunction in vivo. Instead, we state that DYSF expression increases in monocyte/macrophage-lineage cells with aging and that DYSF-expressing F4/80-positive macrophage-lineage cells accumulate in aged skeletal muscle. The functional role of DYSF is supported by gain- and loss-of-function experiments in macrophage cell systems and macrophage–myoblast co-culture assays, including rescue of myogenic differentiation after Dysf knockdown in DYSF-overexpressing macrophages.

We have revised the Abstract, Results, and Discussion accordingly.

The revised Discussion now states:

Line # 422-427

However, several limitations should be noted. First, although our aged-mouse tissue analyses demonstrate the accumulation of DYSF-expressing F4/80-positive macrophage-lineage cells in skeletal muscle, they do not directly establish *in vivo* causality. Macrophage-specific Dysf loss-of-function models, bone marrow transplantation, and muscle injury or aging models will be required to determine whether macrophage DYSF directly contributes to impaired muscle regeneration *in vivo*.

We also revised the overall interpretation of the study to define the present work as identifying a DYSF-associated macrophage mechanism in vitro and an age-associated accumulation of DYSF-expressing macrophage-lineage cells in vivo, rather than establishing direct in vivo causality.

2. While the authors propose a DYSF-Ca^{2+}-STAT1 signaling axis, the evidence remains largely correlative rather than mechanistic. Calcium influx is demonstrated using pharmacological tools and ionophores, but there is no direct demonstration that calcium signaling is required for STAT1 activation downstream of DYSF. Similarly, the necessity of STAT1 for the observed inflammatory phenotype is not tested experimentally. Without such evidence, the proposed signaling cascade remains speculative.

To establish this mechanism rigorously, the authors should perform experiments involving calcium chelation to determine whether blocking intracellular calcium prevents DYSF-induced STAT1 activation and inflammatory marker expression. Additionally, STAT1 inhibition or knockdown experiments should be conducted to test whether STAT1 is required for the DYSF-mediated inflammatory phenotype. Rescue experiments, in which STAT1 activity is restored in the presence of calcium chelation, would further strengthen the proposed linear pathway.

Response:

We thank the reviewer for this important comment. We agree that our original data did not directly establish whether calcium signaling is required for STAT1 activation downstream of DYSF or whether STAT1 is necessary for the DYSF-induced inflammatory phenotype. To address this point, we performed additional mechanistic experiments. First, we inhibited calcium entry using BTP2/SKF96365 or calcium-free conditions and found that DYSF-induced STAT1 Ser727 phosphorylation was attenuated. These results indicate that calcium influx contributes to STAT1 activation downstream of DYSF. Second, we performed STAT1 knockdown experiments and observed that suppression of STAT1 reduced the induction of inflammatory genes and cytokines, including Il6 and Tnf, in DYSF-overexpressing macrophages. These findings support a mechanistic link between DYSF-dependent Ca^{2+} signaling, STAT1 activation, and inflammatory macrophage-like responses.

3. Many of the key findings, including changes in macrophage morphology, cytokine production, migration, and STAT1 activation, are derived from DYSF overexpression in J774A.1 cells. Although knockdown experiments are included, they are less comprehensive and not consistently integrated across all assays. Overexpression can introduce artifacts, particularly for a protein involved in membrane dynamics and calcium handling, and may not accurately reflect physiological regulation of DYSF. The authors should place greater emphasis on loss-of-function approaches throughout the study. Knockdown or knockout of DYSF should be performed systematically across all major phenotypic assays, including polarization marker expression, cytokine secretion, migration, and co-culture experiments. Furthermore, validation in primary macrophages, such as bone marrow-derived macrophages (BMDMs) from wild-type and Dysf-deficient mice, would significantly strengthen the conclusions by demonstrating that the observed phenotypes occur under physiologically relevant conditions. The current reliance on overexpression in a cell line limits the interpretability and generalizability of the findings.

Response:

We thank the reviewer for this important and constructive comment. We agree that reliance on DYSF overexpression in J774A.1 cells is a limitation, particularly because DYSF is a large membrane-associated protein involved in membrane dynamics and Ca^{2+} handling. We also agree that loss-of-function approaches provide a more physiologically relevant way to test the requirement for endogenous Dysf.

In response, we revised the manuscript to place greater emphasis on Dysf loss-of-function experiments. We added and integrated Dysf knockdown data across multiple major assays. Specifically, Dysf knockdown reduced IFN-γ-induced inflammatory macrophage-associated gene expression, including *Nos2/iNOS* and *Stat1*, and reduced TNF-α and IL-6 secretion. Dysf knockdown also attenuated macrophage migration, reduced Ca^{2+} responses, and decreased STAT1 phosphorylation under IFN-γ-stimulated conditions. In addition, in the macrophage–myoblast co-culture system, Dysf knockdown in DYSF-overexpressing macrophages restored myogenic marker expression and Myh1-positive myotube formation. These data are now incorporated into the Results and figures to better balance gain- and loss-of-function evidence

We have also revised the wording throughout the manuscript to avoid interpreting overexpression data alone as evidence of physiological DYSF function. Instead, we now frame the conclusions around bidirectional gain- and loss-of-function evidence, with particular emphasis on the requirement for endogenous Dysf in inflammatory macrophage-associated responses.

We agree that validation in primary macrophages, such as BMDMs from wild-type and Dysf-deficient mice, would further strengthen the physiological relevance of the study. However, these experiments were not completed within the current revision timeframe because they require additional primary cell preparation and validation from appropriate mouse cohorts. We have therefore added this point as a limitation and future direction.

The revised Discussion now states:

Line # 433-439

Second, although DYSF gain-of-function experiments helped test whether increased DYSF abundance promotes inflammatory macrophage-associated responses, overexpression of a large membrane-associated protein may introduce nonphysiological effects. We therefore integrated Dysf loss-of-function experiments across inflammatory gene expression, cytokine secretion, Ca^{2+} responses, STAT1 phosphorylation, migration, and macrophage–myoblast co-culture assays. Nevertheless, validation in primary macrophages, including BMDMs from wild-type and Dysf-deficient mice, will be required to confirm the role of endogenous DYSF under more physiological conditions.

4. The manuscript relies heavily on the M1/M2 dichotomy, supported by a limited set of markers such as iNOS, STAT1, CD80, Arg1, and CD206. This simplified classification does not capture the complexity and heterogeneity of macrophage states, especially in the context of aging and muscle tissue, where macrophages exhibit diverse and context-dependent phenotypes that extend beyond classical M1/M2 categories. A more comprehensive characterization of macrophage phenotypes is needed. The authors should consider employing transcriptomic approaches, such as RNA sequencing, to provide an unbiased assessment of the gene expression changes induced by DYSF modulation.

Response:

We thank the reviewer for this important comment. We agree that the M1/M2 classification is an oversimplified framework and does not adequately capture the complexity, heterogeneity, and context-dependent nature of macrophage states, particularly in aging skeletal muscle. In response, we revised the terminology throughout the manuscript to avoid relying on a strict M1/M2 dichotomy.

We now describe the observed phenotypes using more cautious terms, such as "inflammatory macrophage-like activation," "inflammatory macrophage-associated genes," and "IL-4-induced alternative macrophage-associated markers." In the in vivo tissue context, we no longer define CD80-positive or CD206-positive cells as M1 or M2 macrophages. Instead, we describe these data as CD80-positive cells, CD206-positive cells, or tissue-level macrophage-associated marker immunoreactivity.

We also revised the interpretation of our marker analyses. The expression of Nos2/iNOS, Stat1, Arg1, CD80, CD206, and cytokine production is now presented as evidence of selected inflammatory or alternative macrophage-associated features, rather than as comprehensive definition of macrophage polarization states. In addition, we added STAT1-responsive genes, including *Cxcl10, Irf1, Nos2/iNOS, and Socs1*, to broaden the downstream analysis of DYSF-associated inflammatory signaling.

5. The co-culture experiments between macrophages and myoblasts represent an important attempt to connect macrophage programming to functional consequences in muscle cells. However, these experiments are limited to a Transwell system and primarily attribute the observed effects to TNF-α and IL-6. While cytokine neutralization partially rescues myogenic markers, the analysis remains narrow and does not explore additional signaling pathways, secreted factors, or cell-contact-dependent mechanisms that may contribute to the phenotype. The authors should extend these experiments to include broader secretome analysis, such as multiplex cytokine arrays or proteomic profiling of conditioned media, to identify additional factors that may mediate the effects of DYSF-expressing macrophages on myogenesis. Validation in primary myoblasts or satellite cells, rather than relying solely on the C2C12 cell line, would enhance the physiological relevance of these findings. Additionally, direct co-culture experiments, which allow cell-cell contact, could reveal contact-dependent mechanisms that are not captured in the Transwell system. These extensions would provide a more complete understanding of how DYSF-expressing macrophages influence muscle cell behavior.

Response:

We thank the reviewer for this important and constructive comment. We agree that the Transwell co-culture system specifically evaluates the effects of soluble factors and does not address cell-contact-dependent mechanisms. We also agree that broader secretome analysis and validation using primary myoblasts or satellite cells would provide a more comprehensive understanding of how DYSF-expressing macrophages influence myogenesis.

In the revised manuscript, we have clarified that the Transwell experiments were designed to test paracrine effects of macrophage-derived soluble mediators on myogenic differentiation. We have also revised our interpretation of the TNF-α and IL-6 neutralization experiments. Because neutralization of

either cytokine produced only partial rescue of myogenic marker expression, we no longer state that TNF-α and IL-6 fully mediate the effect. Instead, we now state that TNF-α and IL-6 contribute, at least in part, to the suppression of myogenic differentiation by DYSF-overexpressing macrophages.

To strengthen the functional link between macrophage DYSF and myogenic suppression, we also added Dysf knockdown experiments in the co-culture system. Dysf knockdown in DYSF-overexpressing macrophages restored myogenic marker expression and Myh1-positive myotube formation, supporting the involvement of macrophage DYSF in the suppressive effect on myogenesis.

We agree that multiplex cytokine arrays, proteomic profiling of conditioned media, direct co-culture experiments, and validation using primary myoblasts or satellite cells would further strengthen the physiological relevance of this part of the study. These experiments were not completed within the current revision timeframe. We have therefore revised the Discussion to clearly state these limitations and future directions.

The revised Discussion now states:

Line # 445-451

Fourth, although TNF-α and IL-6 neutralization partially restored myogenic marker expression, the rescue was incomplete, indicating that additional macrophage-derived soluble factors may contribute to the suppression of myogenic differentiation. Because the present co-culture experiments used a Transwell system, they assessed paracrine effects but not cell-contact-dependent mechanisms. Future studies using multiplex cytokine arrays, proteomic profiling, direct macrophage–myoblast co-culture systems, and primary myoblasts or satellite cells will be required to define the full spectrum of DYSF-dependent macrophage-derived signals that regulate myogenesis.

We have also revised the Results section to state that TNF-α and IL-6 are partial contributors rather than sole mediators of the phenotype.

Minor Revisions

1. The manuscript would benefit from thorough language editing to improve clarity and readability. There are multiple instances of awkward phrasing, grammatical inconsistencies, and unclear sentence structures that detract from the overall presentation. Professional editing or careful revision by a native English speaker would enhance the manuscript's accessibility and professionalism.

Response:

We thank the reviewer for this helpful suggestion. The manuscript has been thoroughly edited for clarity, grammar, and readability. We have revised awkward or unclear sentences, corrected grammatical inconsistencies, and standardized terminology throughout the text. We believe these revisions have improved the overall presentation and accessibility of the manuscript.

2. Statistical reporting is not always sufficiently detailed throughout the manuscript. The definitions of biological versus technical replicates are unclear in several experiments, and exact p-values are not consistently reported. The authors should provide complete statistical information, including sample sizes, the number of independent experiments, definitions of error bars, and exact p-values where appropriate. Additionally, some experimental descriptions lack sufficient detail for reproducibility. For example, transfection efficiency is not reported, cytokine concentrations used in some assays are not specified, and the timing of certain treatments is ambiguous. These details should be clarified in the methods section or figure legends.

Response:

We thank the reviewer for this important comment. We have revised the manuscript to provide more detailed statistical and methodological information throughout the Results, Methods, and figure legends. In the revised figures, we clearly define biological and technical replicates. For cell-based assays, values from multiple cells within the same independent culture were averaged, and each independent culture was treated as one biological replicate. For muscle section analyses, values from multiple fields within the same muscle were averaged, and each mouse/muscle was treated as one biological replicate. We have also added sample sizes, the number of independent experiments, definitions of error bars, and exact p-values where appropriate. In addition, we have clarified transfection conditions, cytokine concentrations, treatment durations, and co-culture conditions in the Methods and figure legends.

3. The calcium imaging experiments rely heavily on non-physiological stimuli such as ionomycin and other ionophores to induce calcium influx. While these tools are useful for demonstrating calcium handling capacity, they may not accurately reflect the physiological regulation of calcium signaling downstream of DYSF. The authors should consider including experiments with more physiologically relevant stimuli, such as receptor-mediated calcium mobilization or stimuli that mimic inflammatory conditions, to strengthen the biological relevance of the calcium signaling data.

Response:

We thank the reviewer for this important comment. We agree that ionophores such as A23187 are non-physiological tools and primarily assess calcium handling capacity rather than physiological receptor-mediated calcium signaling.

In response, we revised the manuscript to avoid overinterpreting the ionophore-based experiments. We now describe the A23187 experiments as showing that DYSF modulates intracellular Ca^{2+} handling capacity in macrophages. To further support the involvement of Ca^{2+} entry, we also analyzed thapsigargin-induced store-operated Ca^{2+} entry and showed that DYSF-overexpressing cells exhibited enhanced Ca^{2+} entry after extracellular Ca^{2+} re-addition, which was attenuated by the Ca^{2+} entry inhibitors BTP2 and SKF96365. In addition, we examined Ca^{2+} responses under IFN-γ-stimulated inflammatory conditions and observed that DYSF-overexpressing macrophages showed enhanced Ca^{2+} responses.

However, we agree that these approaches still do not fully reproduce physiological receptor-mediated Ca^{2+} mobilization. Therefore, we have revised the Discussion to clearly state this limitation.

The revised Discussion now states:

Line # 440-444

Third, our Ca^{2+} imaging experiments primarily used ionophore- and SOCE-based approaches. Although A23187 and thapsigargin are useful tools for assessing Ca^{2+} handling capacity and store-operated Ca^{2+} entry, they do not fully recapitulate physiological receptor-mediated Ca^{2+} signaling in macrophages. Future studies using receptor-mediated or inflammatory stimuli in primary macrophages will be required to determine whether and how DYSF regulates physiological Ca^{2+} signaling during macrophage activation.

We have also revised the Results to clarify that our Ca^{2+} imaging data support a role for DYSF in Ca^{2+} handling and Ca^{2+} entry capacity, rather than proving a physiological receptor-mediated Ca^{2+} signaling mechanism.

4. The manuscript places considerable emphasis on STAT1 phosphorylation at Ser727, but the rationale for focusing on this site rather than the canonical Tyr701 phosphorylation is not clearly explained. While Ser727 phosphorylation can modulate STAT1 activity, Tyr701 phosphorylation is typically considered the primary activating event. The authors should provide clearer justification for their focus on Ser727, discuss

the functional significance of this modification in the context of their findings, and ideally include data on Tyr701 phosphorylation to provide a more complete picture of STAT1 activation.

Response:

We thank the reviewer for this important comment. We agree that canonical STAT1 activation is primarily defined by Tyr701 phosphorylation and that our rationale for focusing on Ser727 phosphorylation was not sufficiently explained in the original manuscript.

In response, we revised the manuscript to provide a clearer explanation. We now state that Tyr701 phosphorylation represents the canonical IFN-γ-associated activation step, whereas Ser727 phosphorylation functions as an additional regulatory modification that can modulate STAT1 transcriptional activity and inflammatory gene expression. Because DYSF regulates Ca^{2+} influx and Ca^{2+}-responsive signaling, we examined Ser727 phosphorylation as a potential noncanonical regulatory component of STAT1 signaling downstream of DYSF-associated Ca^{2+} handling.

We also added and clarified data on STAT1 Tyr701 phosphorylation. DYSF overexpression increased STAT1 Ser727 phosphorylation and STAT1 nuclear accumulation, whereas Tyr701 phosphorylation was not markedly enhanced compared with control cells. Conversely, Dysf knockdown reduced STAT1 Ser727 phosphorylation under IFN-γ-stimulated conditions. These results suggest that DYSF preferentially modulates STAT1 Ser727-associated signaling rather than simply enhancing canonical Tyr701-dependent STAT1 activation.

To further support the functional relevance of this pathway, we analyzed STAT1-responsive genes, including *Cxcl10, Irf1, Nos2/iNOS,* and *Socs1*. DYSF-induced expression of these genes was attenuated by Ca^{2+} entry inhibition and by STAT1 knockdown. We have also revised the terminology throughout the manuscript and now refer to "STAT1 Ser727-associated signaling" rather than broadly stating "STAT1 activation."

We also revised the manuscript title of this section to avoid overstatement and to better reflect the scope of our data.

Before:

Ca^{2+} directly regulates STAT1 activation downstream of DYSF.

After:

DYSF Enhances Ca^{2+}-dependent STAT1 Ser727 Phosphorylation and STAT1-responsive Gene Expression.

The revised Discussion now states:

Line # 372-379

Our data indicate that DYSF overexpression preferentially enhanced STAT1 Ser727 phosphorylation without markedly increasing canonical IFN-γ-dependent STAT1 Tyr701 phosphorylation, whereas Dysf knockdown reduced both STAT1 Tyr701 and Ser727 phosphorylation under IFN-γ-stimulated conditions. These findings suggest that DYSF contributes to STAT1 signaling through both regulation of endogenous STAT1 abundance and enhancement of STAT1 Ser727-associated signaling. Importantly, DYSF overexpression did not markedly alter STAT3, STAT6, or NF-κB p65 phosphorylation under the same conditions, supporting a preferential association of DYSF with STAT1-associated signaling in this experimental setting.

We believe these revisions provide a more complete and balanced interpretation of STAT1 signaling in the context of DYSF-dependent macrophage activation.

5. Several figures could be improved in terms of clarity, labeling, and organization. Some panels lack scale bars or have inconsistent labeling conventions. Quantifications are not always clearly linked to their corresponding representative images. The authors should ensure that all figures are clearly labeled, that statistical comparisons are unambiguous, and that figure legends provide sufficient detail to interpret the data without referring extensively to the main text. High-resolution images and consistent formatting across figures would also enhance the overall presentation.

Response: We thank the reviewer for this helpful comment. We have revised the figures to improve clarity, labeling, and consistency throughout the manuscript. Scale bars have been added to all representative microscopy images, and labeling conventions have been standardized across the figures. We have also reorganized several panels so that representative images and their corresponding quantifications are directly linked. Statistical comparisons, sample sizes, biological replicate definitions, and error bars are now clearly indicated in the figure legends. Only biologically relevant statistical comparisons are shown in the graph. Exact P values are provided in the graph or Source Data file. In addition, high-resolution versions of all figure files have been prepared for the revised submission.

Recommendation

In summary, this study presents a potentially novel role for dysferlin in macrophage biology and suggests an interesting link between calcium signaling, STAT1 activation, and inflammatory polarization. However, the current version of the manuscript relies heavily on in vitro systems, lacks definitive mechanistic evidence, and does not provide sufficient in vivo validation to support its central claims. The major revisions outlined above are essential to establish causality, clarify mechanisms, and demonstrate physiological relevance. Substantial additional work would be required to elevate the study to the level expected for publication. I therefore recommend major revision.

Response:

We thank the reviewer for the careful evaluation of our manuscript and for recognizing the potential novelty of DYSF function in macrophage biology. We also appreciate the constructive criticism regarding *in vitro* reliance, mechanistic interpretation, and *in vivo* validation.

In response, we have substantially revised the manuscript to strengthen the mechanistic evidence and to avoid overstatement. We added Ca^{2+} inhibition experiments, STAT1 knockdown experiments, STAT1-responsive gene analyses, and signaling specificity analyses including STAT3, STAT6, and NF-κB p65. We also placed greater emphasis on Dysf loss-of-function experiments across inflammatory gene expression, cytokine secretion, Ca^{2+} responses, STAT1 phosphorylation, migration, and macrophage–myoblast co-culture assays.

To improve *in vivo* relevance, we added F4/80/Dysferlin co-immunostaining in aged skeletal muscle and clarified that the aged-mouse findings are associative rather than causal. We also revised the terminology throughout the manuscript to avoid a strict M1/M2 framework and now describe the phenotype as inflammatory macrophage-associated activation.

We agree that macrophage-specific Dysf loss-of-function models, bone marrow transplantation, injury/aging models, primary macrophage validation, and broader transcriptomic or secretome analyses will be required to fully establish physiological causality. These points are now explicitly discussed as major limitations and future directions.

We believe that the revised manuscript now presents a more balanced and better-supported mechanistic framework for DYSF-associated macrophage inflammatory activation and myogenic suppression.

September 14 2026

Re: Life Science Alliance manuscript #LSA-2026-03669-TR

Dr. Kana Tominaga
Yamaguchi University
Department of Neuroscience
1-1-1 Minami-kogushi, Ube
Yamaguchi 755-8505
Japan

Dear Dr. Tominaga,

Thank you for submitting your manuscript entitled "Dysferlin Promotes Inflammatory Macrophage-associated Activation via Ca^{2+}-dependent STAT1 Signaling" to Life Science Alliance. We apologise for the delay in communication a decision due to reviewer and editor availability issues.

Your revised manuscript was sent back to the original reviewers whose comments are appended below. As you will read, all the reviewers are consistent in their evaluation that the revised version only partly addressed their concerns around causality. We are grateful to Reviewer 3 who has acknowledged the challenge of these in-vivo experiments at this point, and suggested instead a stronger emphasis on this caveat in your manuscript. In addition, Reviewers 2 and 3 have also commented on a lack of clarity in methodological and sample details.

Our general policy is that papers are considered through only one revision cycle. However we are open to one additional short round of revision and we will look for strong reviewer support for a final decision.

In line with the major revisions suggested by the reviewers, we invite you to submit a revised manuscript addressing their concerns. Whilst we do not need any new experimental data, we agree that you must
-explicitly acknowledge the lack of in-vivo evidence upfront in the discussion section (Reviewer 3) and accordingly temper your claims in the abstract on causality.

-expand on your image qualification approach and replicate information as suggested by Reviewers 1 and 3. For mouse work, wherever appropriate, please acknowledge limited statistical power and accordingly temper your conclusions.

-Include the raw quantification data (see Reviewer 3's suggestions) as a supplemental file and callout to this file in your ms text to indicate pre-averaged values.

Please submit the final revision within one month, along with a letter that includes a point by point response to the remaining reviewer comments. Kindly include a revised document with changes highlighted.

To upload the revised version of your manuscript, please log in to your account: https://lsa.msubmit.net/cgi-bin/main.plex

You will be guided to complete the submission of your revised manuscript and to fill in all necessary information. Please get in touch in case you do not know or remember your login name.

The typical timeframe for revisions is three months. Please note that papers are generally considered through only one revision cycle, so strong support from the referees on the revised version is needed for acceptance.

While you are revising your manuscript, please also attend to the below editorial points to help expedite the publication of your manuscript. Please direct any editorial questions to the journal office. When submitting the revision, please include a letter addressing the reviewers' comments point by point.

Thank you for this interesting contribution to Life Science Alliance. We hope that the comments below will prove constructive as your work progresses, and we are looking forward to receiving your revised manuscript.

Sincerely,

Sarita Hebbar, PhD
Scientific Editor
Life Science Alliance
http://www.lsajournal.org

A. THESE ITEMS ARE REQUIRED FOR REVISIONS

-- A letter addressing the reviewers' comments point by point.

-- An editable version of the final text (.DOC or .DOCX) is needed for copyediting (no PDFs).

-- High-resolution figure, supplementary figure and video files uploaded as individual files: See our detailed guidelines for preparing your production-ready images, https://www.life-science-alliance.org/authors

-- Summary blurb (enter in submission system): A short text summarizing in a single sentence the study (max. 200 characters including spaces). This text is used in conjunction with the titles of papers, hence should be informative and complementary to the title and running title. It should describe the context and significance of the findings for a general readership; it should be written in the present tense and refer to the work in the third person. Author names should not be mentioned.

-- By submitting a revision, you attest that you are aware of our payment policies found here: https://www.life-science-alliance.org/copyright-license-fee

B. MANUSCRIPT ORGANIZATION AND FORMATTING:

Full guidelines are available on our Instructions for Authors page, https://www.life-science-alliance.org/authors

We encourage our authors to provide original source data, particularly uncropped/-processed electrophoretic blots and spreadsheets for the main figures of the manuscript. If you would like to add source data, we would welcome one PDF/Excel-file per figure for this information. These files will be linked as supplementary "Source Data" files.

IMPORTANT: It is Life Science Alliance policy that if requested, original data images must be made available. Failure to provide original images upon request will result in unavoidable delays in publication. Please ensure that you have access to all original microscopy and blot data images before submitting your revision.

Reviewer #1 (Comments to the Authors (Required)):

In this revised version of their manuscript, the authors partly addressed the previous comments. They provide new sets of experiments that strengthen the robustness of the mechanism by which DYSF regulates STAT1 to modulate macrophage cell line function in vitro and how this impact fusion of a muscle cell line. They also reanalysed some of their data such as their CSA data more appropriately. Although they globally improved the quality of their manuscript, there are still some important concerns regarding the functional relevance of macrophage Dysf that remains.

Major
1) In order to compare the expression level of Dysf in pro- and anti-inflammatory macrophage populations, the authors used a macrophage cell line polarised using IFNg or IL4 cytokines where they observe a high Dysf expression in pro-inflammatory macrophages and a low expression in anti-inflammatory population in vitro. However, as mentioned in a previous comment, these cytokine treatments do not recapitulate the macrophage status observed in vivo in muscle. Therefore, it is necessary to validate the physiological relevance of this observation in vivo on macrophage subsets isolated from muscle or through the interrogation of already published datasets. Failing to provide evidence of an in vivo difference in Dysf expression between macrophage subsets would significantly lower the impact of the study.

2) The main concern still remains a lack of functional evidence of the role of Dysf in macrophages in vivo, which is a crucial aspect. The increased expression of Dysf in macrophages could be a consequence of muscle aging and not a driver. Unless the authors providence some in vivo evidence that decreasing or removing Dysf expression in macrophages in vivo restores, at least partially, the aging phenotype observed, the data presented remain correlative. If it is too complicated or time consuming to generate aging cohorts, the authors could show that removing/decreasing Dysf expression in muscle macrophages in vivo impacts their inflammatory status and/or function for example in a context of an injury.
In this revised version of the manuscript, the authors chose to dampen their conclusion, but this reduces the potential impact of the study.

Minor
3) The authors now acknowledge that aging is generally associated with sarcopenia and reduced myofiber size and state that their results should be interpreted cautiously. They need to give some elements to try to explain the difference between their observation and the literature.

4) The authors reanalysed their CSA data on several fields of view. They need to mention the number of fields analysed and the mean number of myofibers per sample.

Reviewer #2 (Comments to the Authors (Required)):

The authors have substantially revised the manuscript and made an pivotal effort to address the concerns raised in the previous review. The addition of Dysf loss of function experiments, Ca^{2+} inhibition, STAT1 knockdown, and further co-culture experiments has substantially strengthened the study. The authors have also befittingly moderated the terminology and conclusions, avoiding a rigid M1/M2 classification and reducing some of the previous overstatements. The modern loss of function data provide stronger support for the role of DYSF in pro-inflammatory macrophage associated responses and help address the previous concern regarding the reliance on overexpression. The additional experiments also provide better support for the proposed link between DYSF, Ca^{2+} signaling, STAT1 activation, and the pro-inflammatory phenotype. Overall, the better concerns raised in the previous review have been satisfactorily addressed. The independent odd limitation is the lack of validation in primary macrophages and decisive in vivo evidence for a physiologic role of macrophage DYSF. While such experiments would further strengthen the study, the authors have now befittingly acknowledged these limitations and moderated their conclusions accordingly. For example, confirmation of the DYSF dependent effects on inflammatory activation and/or Ca^{2+}-STAT1 signaling in simple macrophages would provide valuable support for the proposed mechanism. Although the modern data powerfully support a functional link between DYSF dependent Ca^{2+} signaling and STAT1 activation, they do not fully establish a strictly linear or exclusive DYSF-Ca^{2+}-STAT1 pathway. Therefore, the mechanistic conclusions should remain befittingly cautious unless further rescue experiments are performed.

Reviewer #3 (Comments to the Authors (Required)):

I thank the authors for their detailed rebuttal and for the significant effort invested in revising the manuscript. The revised version represents a meaningful improvement in several respects: the terminological shift away from the strict M1/M2 dichotomy is appropriate and well-executed, the addition of calcium entry inhibition experiments and STAT1 knockdown data strengthens the mechanistic argument for the DYSF-Ca^{2+}-STAT1 axis, and the integration of loss-of-function evidence across multiple assays partly addresses my concern about the over-reliance on overexpression. I also appreciate the more cautious framing of the in vivo data as associative rather than causal. Nevertheless, I remain concerned that several of my original points have been addressed only in part, and new issues regarding the quantification of image-based data have emerged from the authors' response that require clarification before the manuscript can be accepted.

My most substantive concern regarding the absence of in vivo loss-of-function evidence - macrophage-specific Dysf deletion, bone marrow transplantation, or muscle injury models - has been acknowledged by the authors, who agree it represents a critical gap, but not experimentally addressed. While I understand that these experiments cannot realistically be completed within the current revision cycle, I want to be explicit that this remains a major limitation of the study as presented. The authors' decision to reframe the aged-mouse data as associative is the correct response given the constraints, but this means the functional claims about DYSF's causal role in age-associated muscle dysfunction rest entirely on cell-based systems. I would strongly encourage the authors to articulate this limitation with even greater prominence in the Discussion - not merely as a passing acknowledgement of future directions, but as an explicit statement that the in vivo relevance of the DYSF-macrophage axis in ageing remains to be demonstrated. The current framing, while improved, still risks implying a degree of in vivo causality that the data do not support.

The authors' rebuttal, for the first time, provides an explanation of how image-based data were handled statistically: for both in vitro and in vivo experiments, multiple cells or fields were measured from each biological replicate (independent culture or mouse), their values were averaged to produce a single value per replicate, and these averaged values were then used as biological replicates for statistical analysis. While this approach is a legitimate strategy for avoiding pseudoreplication, it raises several questions that are not answered in the manuscript and that are essential for the reader to assess the reliability and reproducibility of the quantitative data.

First, the manuscript and its legends do not state whether image quantification was performed manually, semi-automatically, or with automated software. This is not a minor methodological detail: manual quantification of fluorescence intensity, cell morphology parameters, or myotube area is susceptible to observer bias, particularly when the analyst is not blinded to the experimental condition. The authors should state explicitly, for each quantified parameter, whether the analysis was blinded and what software or workflow was used. If quantification was performed manually, the expected inter-observer variability should be acknowledged.

Second, the number of cells, myotubes, or fields that were averaged to produce each dot in the figures is not consistently reported. If each dot represents the mean of, for example, three fields from one mouse or thirty cells from one independent culture, this is information the reader needs to evaluate the biological meaning of the data. A dot that is the average of three

fields and a dot that is the average of thirty fields carry very different weights of evidence, and they will have very different within-replicate variance that is entirely invisible in the current figures. I suggest the authors to report, in each figure legend, the exact number of cells, fields, or myotubes that were quantified per biological replicate.

Third, and most importantly, because each dot represents a mean of multiple measurements rather than a single raw observation, the standard deviation shown in the graphs reflects only between-replicate variance - that is, the variability between independent cultures or between animals - while entirely concealing the within-replicate variance, which is the variability between individual cells, fields, or myofibers within the same replicate. In experiments where the number of biological replicates is small (n = 3 for most in vivo experiments, n = 3-4 for most in vitro assays), this means the reader cannot assess the spread of the underlying data. For this reason, I strongly recommend that the authors provide the complete raw quantification data - the individual measurements before averaging - as a supplementary Excel spreadsheet (Source Data file). This is now standard practice at many journals, and it would allow readers and future investigators to independently assess the within-replicate variability, check for outliers, and reproduce the statistical analysis. If a Source Data file is already provided, the authors should draw explicit attention to it and ensure that it contains the pre-averaged individual measurements and not only the replicate-level means that are plotted in the figures.

For the skeletal muscle histological analyses, several key comparisons are based on n = 3 mice per group, which is a small sample for experiments involving aged animals, where biological variability is known to be substantially higher than in young adult cohorts. While I recognise that aged mouse cohorts are resource-intensive and difficult to expand within a revision window, n = 3 is at the lower boundary of what most journals and biostatistical guidelines would consider sufficient for drawing robust conclusions from histological measurements. In particular, the quantification of CD80-positive and CD206-positive cells in aged versus young muscle, and the F4/80+Dysferlin+ co-staining data, would be considerably more persuasive with at least n = 5 animals per group. If increasing the sample size is not feasible at this stage, the authors should explicitly acknowledge the limited statistical power of the n = 3 comparisons and refrain from drawing strong conclusions from these specific datasets in the absence of supportive power analyses.

Conclusion. The revised manuscript is substantially improved relative to the original submission, and the authors have responded to my concerns constructively and in good faith. However, I cannot recommend acceptance until the image quantification methodology is described with full transparency - including the blinding procedure, the software used, and the number of cells or fields contributing to each data point - and until a Source Data file containing pre-averaged raw measurements is made available. The low animal numbers in the in vivo histological analyses should also be explicitly discussed as a limitation.

Point-by-point to the comments from the Reviewers

Reviewer #1 (Comments to the Authors (Required)):

In this revised version of their manuscript, the authors partly addressed the previous comments. They provide new sets of experiments that strengthen the robustness of the mechanism by which DYSF regulates STAT1 to modulate macrophage cell line function in vitro and how this impact fusion of a muscle cell line. They also reanalysed some of their data such as their CSA data more appropriately. Although they globally improved the quality of their manuscript, there are still some important concerns regarding the functional relevance of macrophage Dysf that remains.

Major

1) In order to compare the expression level of Dysf in pro- and anti-inflammatory macrophage populations, the authors used a macrophage cell line polarised using IFNg or IL4 cytokines where they observe a high Dysf expression in pro-inflammatory macrophages and a low expression in anti-inflammatory population in vitro. However, as mentioned in a previous comment, these cytokine treatments do not recapitulate the macrophage status observed in vivo in muscle. Therefore, it is necessary to validate the physiological relevance of this observation in vivo on macrophage subsets isolated from muscle or through the interrogation of already published datasets. Failing to provide evidence of an in vivo difference in Dysf expression between macrophage subsets would significantly lower the impact of the study.

Response:

We thank the reviewer for this important comment. We agree that macrophage states induced by IFN-γ or IL-4 *in vitro* do not recapitulate the full phenotypic diversity of macrophage populations present in skeletal muscle *in vivo*. Our intention in using these cytokine treatments was to examine DYSF expression under defined inflammatory and alternative activation conditions, rather than to model discrete macrophage subsets found in muscle.

In line with the Editor's guidance that no additional experimental data are required for this revision, we have not added new *in vivo* macrophage-subset analyses. Instead, we have further revised the manuscript to avoid extrapolating the IFN-γ or IL-4 experiments to specific macrophage populations in skeletal muscle. We now describe these experiments as defined *in vitro* macrophage activation conditions and explicitly acknowledge that whether DYSF expression differs among macrophage states in skeletal muscle *in vivo* remains to be established.

We have also added this limitation to the Discussion and now state that future studies using sorted muscle macrophage populations and single-cell transcriptomic approaches will be required to determine the relationship between DYSF expression and macrophage heterogeneity *in vivo*.

The following statement has been added to the Discussion (Lines 410-412): Furthermore, the cytokine-defined macrophage states used in vitro do not fully recapitulate macrophage heterogeneity in skeletal muscle, and the distribution of DYSF among macrophage subpopulations *in vivo* remains to be established.

2) The main concern still remains a lack of functional evidence of the role of Dysf in macrophages in vivo, which is a crucial aspect. The increased expression of Dysf in macrophages could be a consequence of muscle aging and not a driver. Unless the authors providence some in vivo evidence that decreasing or removing Dysf expression in macrophages in vivo restores, at least partially, the aging phenotype observed, the data presented remain correlative. If it is too complicated or time consuming to generate aging cohorts, the authors could show that removing/decreasing Dysf expression in muscle macrophages in vivo impacts their inflammatory status and/or function for example in a context of an injury.

In this revised version of the manuscript, the authors chose to dampen their conclusion, but this reduces the potential impact of the study.

Response:

We thank the reviewer for reiterating this important concern. We fully agree that the present study does not establish that increased macrophage DYSF is a causal driver of age-associated skeletal muscle alterations *in vivo*. As the reviewer correctly points out, the increased abundance of DYSF-expressing macrophage-lineage cells in aged skeletal muscle could represent a consequence of the altered tissue environment associated with aging rather than a causal mechanism.

In line with the Editor's guidance, we have not added new *in vivo* experimental data in this revision. Instead, we have substantially strengthened the distinction between the functional evidence obtained in cell-based systems and the associative observations obtained in aged mouse muscle.

Specifically, we now state at the beginning of the Discussion that, although our gain- and loss-of-function experiments support a functional role for DYSF in macrophage inflammatory responses and suppression of myogenic differentiation *in vitro*, the aged-mouse data are associative and do not establish that macrophage DYSF causally contributes to age-associated muscle dysfunction. We further state that macrophage-specific Dysf loss-of-function, bone marrow transplantation, or muscle injury models will be required to establish the physiological and causal relevance of macrophage DYSF *in vivo*.

We have also further tempered the Abstract and the Discussion so that the manuscript no longer implies that macrophage DYSF drives age-associated muscle dysfunction *in vivo*.

The concluding sentence of the Abstract has been revised as follows (Line 21-23): Together, these findings support a role for DYSF in Ca^{2+}-dependent STAT1-associated inflammatory responses in macrophages and in the suppression of myogenesis in cell-based systems.

The following statement has been added to the Discussion (Lines 401-402): Thus, the physiological and causal relevance of macrophage DYSF in skeletal muscle aging remains to be demonstrated.

Minor

3) The authors now acknowledge that aging is generally associated with sarcopenia and reduced myofiber size and state that their results should be interpreted cautiously. They need to give some elements to try to explain the difference between their observation and the literature.

Response:

We thank the reviewer for this suggestion. We agree that the apparent increase in myofiber cross-sectional area observed in our aged cohort differs from the commonly reported reduction in myofiber size associated with aging and sarcopenia.

We have therefore expanded the Discussion to address possible explanations for this discrepancy. These include differences in muscle type and fiber-type composition, the marked structural heterogeneity of aged skeletal muscle, and sampling-related effects. Selective loss or atrophy of particular fiber populations, together with age-associated changes in muscle architecture, may also contribute to differences in the fibers represented within the analyzed fields. Because our study was not designed to investigate fiber-type-specific aging responses, these possibilities cannot be distinguished with the present dataset.

Accordingly, we now explicitly state that the observed CSA difference should be interpreted cautiously and may not fully reflect the overall distribution of myofiber size in aged muscle.

The following statement has been added to the Discussion (Lines 404-410): In addition, some histological analyses were based on a limited number of animals, restricting statistical power. The apparent increase in CSA observed in the aged cohort differs from the reduction in myofiber size commonly associated with aging and may reflect differences in muscle type, fiber-type composition, age-associated tissue heterogeneity, and sampling. Because the CSA analysis was based on a limited number of fields and fibers, the apparent increase in CSA should be interpreted cautiously and may not fully reflect the overall distribution of myofiber size in aged muscle.

4) The authors reanalysed their CSA data on several fields of view. They need to mention the number of fields analysed and the mean number of myofibers per sample.

Response:

We thank the reviewer for this comment. CSA was included as a supplementary histological analysis. We have now clarified that two microscopic fields were acquired per mouse and that a total of 10 myofibers across these fields were quantified per mouse, with five mice analyzed per group. The individual fiber measurements were averaged to obtain one value per mouse. We have also prepared a Source Data file containing the pre-averaged individual myofiber CSA measurements underlying this analysis, allowing the reader to examine the within-mouse variability as well as the replicate-level values shown in the figure.

The following statement has been added to the legend of Supplementary figure 6B: Myofiber CSA was quantified from two microscopic fields in each muscle section, with a total of 10 myofibers analyzed per mouse. Values from all analyzed myofibers within the same mouse were averaged before statistical analysis, and each mouse was treated as one biological replicate. Each dot represents one mouse (n = 5 mice per group). Pre-averaged individual myofiber measurements are provided in the corresponding Source Data file.

Reviewer #2 (Comments to the Authors (Required)):

The authors have substantially revised the manuscript and made an pivotal effort to address the concerns raised in the previous review. The addition of Dysf loss of function experiments, Ca^{2+} inhibition, STAT1 knockdown, and further co-culture experiments has substantially strengthened the study. The authors have also befittingly moderated the terminology and conclusions, avoiding a rigid M1/M2 classification and reducing some of the previous overstatements. The modern loss of function data provide stronger support for the role of DYSF in pro-inflammatory macrophage associated responses and help address the previous concern regarding the reliance on overexpression. The additional experiments also provide better support for the proposed link between DYSF, Ca^{2+} signaling, STAT1 activation, and the pro-inflammatory phenotype. Overall, the better concerns raised in the previous review have been satisfactorily addressed. The independent odd limitation is the lack of validation in primary macrophages and decisive in vivo evidence for a physiologic role of macrophage DYSF. While such experiments would further strengthen the study, the authors have now befittingly acknowledged these limitations and moderated their conclusions accordingly. For example, confirmation of the DYSF dependent effects on inflammatory activation and/or Ca^{2+}-STAT1 signaling in simple macrophages would provide valuable support for the proposed mechanism. Although the modern data powerfully support a functional link between DYSF dependent Ca^{2+} signaling and STAT1 activation, they do not fully establish a strictly linear or exclusive DYSF-Ca^{2+}-STAT1 pathway. Therefore, the mechanistic conclusions should remain befittingly cautious unless further rescue experiments are performed.

Response to Reviewer #2:

We sincerely thank the reviewer for the careful re-evaluation of our manuscript and for recognizing the substantial improvements made in the revised version. We greatly appreciate the reviewer's positive

assessment of the newly added *Dysf* loss-of-function experiments, Ca^{2+} inhibition studies, STAT1 knockdown experiments, and additional co-culture analyses. We are also grateful that the reviewer considers our revised terminology and more cautious interpretation of macrophage activation to be appropriate.

In this revision, we have further refined the manuscript to ensure that the conclusions remain fully supported by the experimental evidence and to clearly distinguish the functional findings obtained in cell-based systems from the associative observations obtained in aged skeletal muscle.

We also agree that validation in primary macrophages and direct functional evidence *in vivo* would provide important additional support for the physiological relevance of macrophage DYSF. In line with the Editor's guidance that no additional experimental data are required for this revision, we have not added new primary macrophage or *in vivo* experiments. Instead, we have further strengthened the discussion of this limitation and more clearly separated the mechanistic conclusions derived from our cell-based experiments from the associative findings in aged skeletal muscle.

We fully agree with the reviewer that our data support a functional relationship between DYSF, Ca^{2+} signaling and STAT1 activation, but do not establish directly a strictly linear or exclusive DYSF–Ca^{2+}–STAT1 signaling pathway. We have therefore further revised the manuscript to avoid implying such a linear or exclusive mechanism. We have also clarified in the Discussion that additional signaling pathways may contribute to the inflammatory phenotype associated with DYSF expression and that further mechanistic studies will be required to determine the precise molecular relationship between DYSF, Ca^{2+} signaling and STAT1 activation.

The title has been revised as follows: Dysferlin Promotes Inflammatory Macrophage Responses and Modulates Ca^{2+}-dependent STAT1 Signaling

The concluding sentence of the Abstract has been revised as follows (Line 21-23): Together, these findings support a role for DYSF in Ca^{2+}-dependent STAT1-associated inflammatory responses in macrophages and in the suppression of myogenesis in cell-based systems.

The following statement has been added to the Discussion (Lines 333): Our findings support a role for DYSF in regulating macrophage activation in association with Ca^{2+} signaling.

The following statement has been added to the Discussion (Lines 376-377): DYSF-associated changes in Ca^{2+} signaling may contribute to inflammatory macrophage responses.

The following statement has been added to the Discussion (Lines 397-398): Our findings support a functional link between DYSF, Ca^{2+} signaling, STAT1-associated inflammatory responses, and macrophage-muscle cell communication.

The following statement has been added to the Discussion (Lines 401-403): Thus, the physiological and causal relevance of macrophage DYSF in skeletal muscle aging remains to be demonstrated.

The following statement has been added to the Discussion (Lines 417-419): Nevertheless, validation in primary macrophages, including BMDMs from wild-type and Dysf-deficient mice, will be required to confirm the role of endogenous DYSF under more physiological conditions.

The following statement has been added to the Discussion (Lines 420-424): Moreover, although the Ca^{2+} inhibition and STAT1 loss-of-function experiments support a functional relationship between DYSF-dependent Ca^{2+} signaling and STAT1 activation, they do not establish a strictly linear or exclusive DYSF–Ca^{2+}–STAT1 pathway. Further studies using primary macrophages and physiological stimuli will be required to define this mechanism.

Reviewer #3 (Comments to the Authors (Required)):

I thank the authors for their detailed rebuttal and for the significant effort invested in revising the manuscript. The revised version represents a meaningful improvement in several respects: the terminological shift away from the strict M1/M2 dichotomy is appropriate and well-executed, the addition of calcium entry inhibition experiments and STAT1 knockdown data strengthens the mechanistic argument for the DYSF-Ca^{2+}-STAT1 axis, and the integration of loss-of-function evidence across multiple assays partly addresses my concern about the over-reliance on overexpression. I also appreciate the more cautious framing of the in vivo data as associative rather than causal. Nevertheless, I remain concerned that several of my original points have been addressed only in part, and new issues regarding the quantification of image-based data have emerged from the authors' response that require clarification before the manuscript can be accepted.

My most substantive concern regarding the absence of in vivo loss-of-function evidence - macrophage-specific Dysf deletion, bone marrow transplantation, or muscle injury models - has been acknowledged by the authors, who agree it represents a critical gap, but not experimentally addressed. While I understand that these experiments cannot realistically be completed within the current revision cycle, I want to be explicit that this remains a major limitation of the study as presented. The authors' decision to reframe the aged-mouse data as associative is the correct response given the constraints, but this means the functional claims about DYSF's causal role in age-associated muscle dysfunction rest entirely on cell-based systems. I would strongly encourage the authors to articulate this limitation with even greater prominence in the Discussion - not merely as a passing acknowledgement of future directions, but as an explicit statement that the in vivo relevance of the DYSF-macrophage axis in ageing remains to be demonstrated. The current framing, while improved, still risks implying a degree of in vivo causality that the data do not support.

The authors' rebuttal, for the first time, provides an explanation of how image-based data were handled statistically: for both in vitro and in vivo experiments, multiple cells or fields were measured from each biological replicate (independent culture or mouse), their values were averaged to produce a single value per replicate, and these averaged values were then used as biological replicates for statistical analysis. While this approach is a legitimate strategy for avoiding pseudoreplication, it raises several questions that are not answered in the manuscript and that are essential for the reader to assess the reliability and reproducibility of the quantitative data.

First, the manuscript and its legends do not state whether image quantification was performed manually, semi-automatically, or with automated software. This is not a minor methodological detail: manual quantification of fluorescence intensity, cell morphology parameters, or myotube area is susceptible to observer bias, particularly when the analyst is not blinded to the experimental condition. The authors should state explicitly, for each quantified parameter, whether the analysis was blinded and what software or workflow was used. If quantification was performed manually, the expected inter-observer variability should be acknowledged.

Second, the number of cells, myotubes, or fields that were averaged to produce each dot in the figures is not consistently reported. If each dot represents the mean of, for example, three fields from one mouse or thirty cells from one independent culture, this is information the reader needs to evaluate the biological meaning of the data. A dot that is the average of three fields and a dot that is the average of thirty fields carry very different weights of evidence, and they will have very different within-replicate variance that is entirely invisible in the current figures. I suggest the authors to report, in each figure legend, the exact number of cells, fields, or myotubes that were quantified per biological replicate.

Third, and most importantly, because each dot represents a mean of multiple measurements rather than a single raw observation, the standard deviation shown in the graphs reflects only between-replicate variance - that is, the variability between independent cultures or between animals - while entirely

concealing the within-replicate variance, which is the variability between individual cells, fields, or myofibers within the same replicate. In experiments where the number of biological replicates is small (n = 3 for most in vivo experiments, n = 3-4 for most in vitro assays), this means the reader cannot assess the spread of the underlying data. For this reason, I strongly recommend that the authors provide the complete raw quantification data - the individual measurements before averaging - as a supplementary Excel spreadsheet (Source Data file). This is now standard practice at many journals, and it would allow readers and future investigators to independently assess the within-replicate variability, check for outliers, and reproduce the statistical analysis. If a Source Data file is already provided, the authors should draw explicit attention to it and ensure that it contains the pre-averaged individual measurements and not only the replicate-level means that are plotted in the figures.

For the skeletal muscle histological analyses, several key comparisons are based on n = 3 mice per group, which is a small sample for experiments involving aged animals, where biological variability is known to be substantially higher than in young adult cohorts. While I recognise that aged mouse cohorts are resource-intensive and difficult to expand within a revision window, n = 3 is at the lower boundary of what most journals and biostatistical guidelines would consider sufficient for drawing robust conclusions from histological measurements. In particular, the quantification of CD80-positive and CD206-positive cells in aged versus young muscle, and the F4/80+Dysferlin+ co-staining data, would be considerably more persuasive with at least n = 5 animals per group. If increasing the sample size is not feasible at this stage, the authors should explicitly acknowledge the limited statistical power of the n = 3 comparisons and refrain from drawing strong conclusions from these specific datasets in the absence of supportive power analyses.

Conclusion. The revised manuscript is substantially improved relative to the original submission, and the authors have responded to my concerns constructively and in good faith. However, I cannot recommend acceptance until the image quantification methodology is described with full transparency - including the blinding procedure, the software used, and the number of cells or fields contributing to each data point - and until a Source Data file containing pre-averaged raw measurements is made available. The low animal numbers in the in vivo histological analyses should also be explicitly discussed as a limitation.

Response to Reviewer #3:

We sincerely thank the reviewer for the careful re-evaluation of our manuscript and for recognizing the substantial improvements made in the revised version. We have carefully addressed the remaining concerns regarding in vivo causality, image quantification, Source Data, and the limited statistical power of some mouse experiments.

First, we agree that the present study does not establish a causal role for macrophage DYSF in age-associated muscle dysfunction in vivo. In line with the Editor's guidance, we have not added new in vivo experiments, but have further tempered the Abstract and Discussion. We now explicitly state near the beginning of the Discussion that the aged-mouse findings are associative and that the physiological and causal relevance of macrophage DYSF in skeletal muscle aging remains to be demonstrated.

Second, we have expanded the Methods and figure legends to clarify the image quantification procedures. We now specify the software and analysis workflow used and the number of cells, fields, myotubes, or myofibers contributing to each biological replicate. Image acquisition and quantification were not performed under blinded conditions; however, identical acquisition settings and analysis criteria were applied across all experimental groups. We also clarify that measurements obtained within the same independent culture or mouse were averaged before statistical analysis and that each independent culture or mouse was treated as one biological replicate.

Third, we have prepared Source Data files containing the pre-averaged individual measurements underlying the image-based quantifications, allowing assessment of within-replicate variability and

direct comparison with the replicate-level values shown in the figures. These files are now explicitly referenced in the corresponding figure legends.

Finally, we acknowledge that some histological analyses were based on a limited number of animals (n = 3 per group), resulting in limited statistical power. We have added this limitation to the Discussion and further tempered the interpretation of these findings.

We thank the reviewer for these suggestions, which have substantially improved the transparency and reproducibility of the manuscript.We thank the reviewer for these suggestions, which have substantially improved the transparency and reproducibility of the manuscript.

The following statements have been added to the Discussion (Lines 327–329): However, these aged-mouse findings are associative and do not establish that macrophage DYSF causally contributes to age-associated muscle alterations *in vivo*.

The following statements have been added to the Discussion (Lines 401–402): Thus, the physiological and causal relevance of macrophage DYSF in skeletal muscle aging remains to be demonstrated.

The following statements have been added to the Discussion (Lines 404–410): In addition, some histological analyses were based on a limited number of animals, restricting statistical power. The apparent increase in CSA observed in the aged cohort differs from the reduction in myofiber size commonly associated with aging and may reflect differences in muscle type, fiber-type composition, age-associated tissue heterogeneity, and sampling. Because the CSA analysis was based on a limited number of fields and fibers, the apparent increase in CSA should be interpreted cautiously and may not fully reflect the overall distribution of myofiber size in aged muscle.

The following statement has been added to the Methods of Histochemical and Immunofluorescence staining: Image acquisition and quantification were not performed under blinded conditions; however, identical acquisition settings and analysis criteria were applied across all experimental groups.

The image quantification procedures, replicate definitions, and numbers of cells, fields, myotubes, or myofibers analyzed have also been clarified in the Methods and corresponding figure legends.

September 22, 2026

RE: Life Science Alliance Manuscript #LSA-2026-03669-TRR

Dr. Kana Tominaga
Yamaguchi University
Department of Neuroscience
1-1-1 Minami-kogushi, Ube
Yamaguchi 755-8505
Japan

Dear Dr. Tominaga,

Thank you for submitting your revised manuscript entitled "Dysferlin Promotes Inflammatory Macrophage Responses and Modulates Ca^{2+}-dependent STAT1 Signaling", and addressing the reviewers' concerns in expanding your discussion, providing clarity to the methods, and including requested source data.

We would be happy to publish your paper in Life Science Alliance pending final revisions necessary to meet our formatting guidelines.

MANUSCRIPT ORGANIZATION AND FORMATTING:

To avoid unnecessary delays in the acceptance and publication of your paper, please read the following information carefully. Full guidelines are available on our Instructions for Authors page, https://www.life-science-alliance.org/authors

Submission of a paper that does not conform to Life Science Alliance guidelines will delay the acceptance of your manuscript.

-We offer the following change (for clarity) to the last sentence in the abstract, "Together, these findings support a role for DYSF in Ca^{2+}-dependent STAT1-associated inflammatory responses in macrophages and in the suppression of myogenesis in cellular models".
-Please add the X and Bluesky handles of your host institute/organization, as well as your own, and/or one of the authors, in our system.
-Please upload a clean file without the colored text. The version that highlights the changes as "Related manuscript file".
-Please rename "Competing interests" to "Conflict of Interests".
-Panel B is not labeled in Figure S4. Please correct.
-Thank you for providing a statement on data availability. Please clarify the first sentence by stating if all data described in the manuscript are provided as source data or included in the manuscript.
-Thank you for providing the requested source data; please confirm and correct the figure ID in row 1 for the tab titled, 'FigureS6D_CD80'. It is stated as 'Figure S6F' in Row 1.
-Please be sure that the authorship listing and order is correct.

We encourage our authors to provide original source data, particularly uncropped/-processed electrophoretic blots and spreadsheets for the main figures of the manuscript. If you would like to add source data, we would welcome one PDF/Excel-file per figure for this information. These files will be linked as supplementary "Source Data" files.

It is Life Science Alliance policy that if requested, original data images must be made available to the editors. Failure to provide original images upon request will result in unavoidable delays in publication. Please ensure that you have access to all original data images prior to final submission.

If you are planning a press release on your work, please inform us immediately to allow informing our production team and scheduling a release date.

We welcome submissions of potential cover images for the issue of LSA in which your work would appear. If you have high quality images associated with this work, please feel free to email these, with a caption, to the journal office.

LSA encourages authors to provide a 30-60 second video where the study is briefly explained. These videos will be appear embedded with the manuscript online at Life Science Alliance, and on social media to promote the published paper and authors (for examples, see https://docs.google.com/document/d/1-UWCfbE4pGcDdcgzcmiuJl2XMBJnxKYeqRvLLrLSo8s/edit?usp=sharing). Corresponding or first-authors are welcome to submit the video. Please submit only one video per manuscript. The video can be emailed to contact@life-science-alliance.org

FINAL FILES:

To upload the final version of your manuscript, please log in to your account: https://lsa.msubmit.net/cgi-bin/main.plex
You will be guided to complete the submission of your revised manuscript and to fill in all necessary information. Please get in touch in case you do not know or remember your login name.

The following items are required for acceptance.

-- An editable version of the final text (.DOC or .DOCX) is needed for copyediting (no PDFs).

-- High-resolution figure, supplementary figure and video files uploaded as individual files: See our detailed guidelines for preparing your production-ready images, https://www.life-science-alliance.org/authors

-- Summary blurb (enter in submission system): A short text summarizing in a single sentence the study (max. 200 characters including spaces). This text is used in conjunction with the titles of papers, hence should be informative and complementary to the title. It should describe the context and significance of the findings for a general readership; it should be written in the present tense and refer to the work in the third person. Author names should not be mentioned.

The license to publish form must be signed before your manuscript can be sent to production. A link to the license to publish form will be available to the corresponding author only. Please take a moment to check your funder requirements.

Reviews, decision letters, and point-by-point responses associated with peer-review at Life Science Alliance will be published, alongside the manuscript. If you do want to opt out of having the reviewer reports and your point-by-point responses displayed, please let us know immediately.

Thank you for your attention to these final processing requirements. Please revise and format the manuscript and upload materials as soon as you are able.

Thank you for this interesting contribution to the literature. We look forward to publishing your paper in Life Science Alliance.

Sincerely,

Sarita Hebbar, PhD
Scientific Editor
Life Science Alliance
http://www.lsajournal.org

September 26, 2026

RE: Life Science Alliance Manuscript #LSA-2026-03669-TRRR

Dr. Kana Tominaga
Yamaguchi University
Department of Neuroscience
1-1-1 Minami-kogushi, Ube
Yamaguchi 755-8505
Japan

Dear Dr. Tominaga,

Thank you for submitting your Research Article entitled "Dysferlin Promotes Inflammatory Macrophage Responses and Modulates Ca^{2+}-dependent STAT1 Signaling". It is a pleasure to let you know that your manuscript is now accepted for publication in Life Science Alliance. Congratulations on this interesting work.

The final published version of your manuscript will be deposited by us to PubMed Central upon publication.

Your manuscript will now progress through copyediting and proofing. At this stage, please correct age of young mice in the legend of Figure 5A (line 829) so it accurately reflects the information in the methods (line 437).

It is journal policy that authors provide original data upon request.

Your article will publish open access upon publication under a CC-BY license.

Reviews, decision letters, and point-by-point responses associated with peer-review at Life Science Alliance will be published, alongside the manuscript. If you do want to opt out of having the reviewer reports and your point-by-point responses displayed, please let us know immediately.

IMPORTANT: If you will be unreachable at any time, please provide us with the email address of an alternate author. Failure to respond to routine queries may lead to unavoidable delays in publication.

Scheduling details will be available from our production department. You will receive proofs shortly before the publication date. Only essential corrections can be made at the proof stage so if there are any minor final changes you wish to make to the manuscript, please let the journal office know now.

DISTRIBUTION OF MATERIALS:
Authors are required to distribute freely any materials used in experiments published in Life Science Alliance. Authors are encouraged to deposit materials used in their studies to the appropriate repositories for distribution to researchers.

You can contact the journal office with any questions, contact@life-science-alliance.org

Again, congratulations on a very nice paper. I hope you found the review process to be constructive and are pleased with how the manuscript was handled editorially. We look forward to future exciting submissions from your lab.

Sincerely,

Sarita Hebbar, PhD
Scientific Editor
Life Science Alliance
http://www.lsajournal.org
